# Chemical Profiling and Anti-Inflammatory Mechanisms of *Heracleum millefolium Diels* Revealed by Molecular Networking, Network Pharmacology, and Cellular Validation

**DOI:** 10.3390/metabo16080573

**Published:** 2026-08-13

**Authors:** Genhua Zhu, Xueqin Yin, Ciren Dunzhu, Xiang Zhou, Meijuan Shao, En Yuan, Zhihong Yan, Xiaoyu Xie

**Affiliations:** 1 Key Laboratory of Modern Preparation of Traditional Chinese Medicine, Ministry of Education, Jiangxi University of Chinese Medicine, Nanchang 330100, China; 20020688@jxutcm.edu.cn (G.Z.); yinxueqin@jxutcm.edu.cn (X.Y.); 20121011@jxutcm.edu.cn (X.Z.); 2 Department of Traditional Chinese Medicine, University of Tibetan Medicine, Lhasa 850000, China; 2003001@ttmc.edu.cn; 3 Department of Pharmacy, Jiangxi University of Chinese Medicine, Nanchang 330100, China; shaomeijuan@jxutcm.edu.cn (M.S.); 20144031@jxutcm.edu.cn (E.Y.)

**Keywords:** anti-inflammatory activity, cellular validation, *Heracleum millefolium Diels*, molecular networking, network pharmacology, UPLC-HRMS

## Abstract

**Background/Objectives**: *Heracleum millefolium Diels* (HMD) is a traditional Tibetan medicinal herb that has been reported to possess analgesic, anti-edematous, and anti-inflammatory activities. However, its chemical composition and anti-inflammatory mechanisms remain unclear. **Methods**: In this study, ultra-performance liquid chromatography coupled with high-resolution mass spectrometry (UPLC-HRMS), combined with database searching and “seed”-based molecular networking, was used to systematically characterize the chemical constituents of HMD. Furthermore, an integrated strategy combining network pharmacology, molecular docking, and cellular validation was applied to explore potential anti-inflammatory mechanisms associated with HMD. **Results**: A total of 472 compounds were identified or tentatively identified from HMD. Network pharmacology analysis predicted five key targets, namely TNF, IL-6, IL-1β, GAPDH, and AKT1, together with four potential bioactive constituents, including velutin, artemitin, kaempferol, and naringenin. Pathway enrichment analysis indicated that the potential anti-inflammatory effects of HMD were mainly associated with lipid and atherosclerosis and the AGE-RAGE signaling pathway. Molecular docking suggested potential favorable interactions between the selected constituents and key targets. Moreover, cellular experiments demonstrated that the four compounds significantly inhibited inflammatory responses in LPS-stimulated RAW264.7 macrophages. **Conclusions**: This study systematically characterized the chemical profile of HMD and preliminarily explored its potential anti-inflammatory mechanisms, providing a scientific basis for further investigation of its bioactive constituents and pharmacological properties.

## 1. Introduction

*Heracleum millefolium Diels* (HMD) is the dried whole plant of *Heracleum millefolium* belonging to the family Apiaceae and is traditionally used in Tibetan medicine for the treatment of inflammatory disorders, pain, and edema [1]. Inflammation is a critical pathological process involved in various diseases, and natural products with multi-target and low-toxicity properties have attracted increasing attention as potential anti-inflammatory agents [2,3]. Phytochemical studies have revealed that HMD contains diverse classes of natural products, such as coumarins, flavonoids, and organic acids, which may contribute to its reported biological activities [4]. However, these investigations have primarily relied on targeted analysis or qualitative screening of selected compound classes. Previous pharmacological studies have provided preliminary evidence supporting the biological potential of HMD. For example, the essential oil extracted from HMD protected HaCaT keratinocytes against radiation-induced oxidative damage, while its total flavonoid fraction exhibited antioxidant activity, suggesting contains constituents with potential biological activities [5,6]. Moreover, an acute toxicity study in mice demonstrated a high LD_5_ value (61.30 g/kg) for the total extract, suggesting a preliminary safety profile [7]. However, these studies mainly focused on general biological activities or specific fractions of HMD, and the chemical basis underlying its anti-inflammatory effects remains poorly understood. Therefore, a comprehensive characterization of the chemical composition of HMD, together with the systematic identification of bioactive constituents associated with its anti-inflammatory activity, has not yet been established.

To address this knowledge gap, advanced analytical approaches are required to comprehensively characterize the complex chemical composition of HMD and elucidate the relationship between its chemical constituents and biological activities. The intrinsic chemical complexity and structural diversity of traditional Tibetan medicines greatly hinder the comprehensive characterization of HMD constituents [8]. Ultra-performance liquid chromatography–high-resolution mass spectrometry (UPLC-HRMS) has become a powerful tool for natural product research due to its high sensitivity, accurate mass measurement, and broad chemical coverage [9,10,11]. However, conventional LC-MS annotation strategies largely rely on database matching, which may provide limited confidence for structurally related compounds and fail to fully reveal the chemical diversity of complex herbal medicines [12]. Molecular networking complements conventional annotation strategies by organizing MS/MS spectra according to fragmentation similarity, enabling the visualization of chemical relationships among structurally related compounds and facilitating the annotation of structurally related or previously unannotated constituents [13]. Therefore, integrating LC-HRMS with molecular networking offers advantages over conventional database-based annotation alone, particularly for chemically complex natural products. Recent studies have demonstrated the effectiveness of LC-HRMS combined with molecular networking for comprehensive chemical characterization of natural medicines. For example, researchers successfully expanded the annotation coverage of *Alstonia scholaris* and *Scrophularia ningpoensis* by integrating molecular networking with HRMS analysis [14,15]. These studies highlight the potential of this strategy for systematic chemical profiling of complex medicinal plants.

However, comprehensive chemical characterization alone is insufficient to elucidate the pharmacological basis of HMD. Because traditional medicines usually exert their effects through multiple constituents and targets, network pharmacology provides a systems-level strategy to explore potential compound-target-pathway relationships, while molecular docking offers structural insights into compound-protein interactions [16,17,18]. Nevertheless, computational predictions alone cannot fully reflect biological activity; therefore, experimental validation using appropriate cellular models is essential to evaluate the biological relevance of predicted compounds and pathways. The integration of network pharmacology, molecular docking, and cellular validation provides a complementary strategy for linking chemical constituents with their potential pharmacological mechanisms.

**Figure 1:**
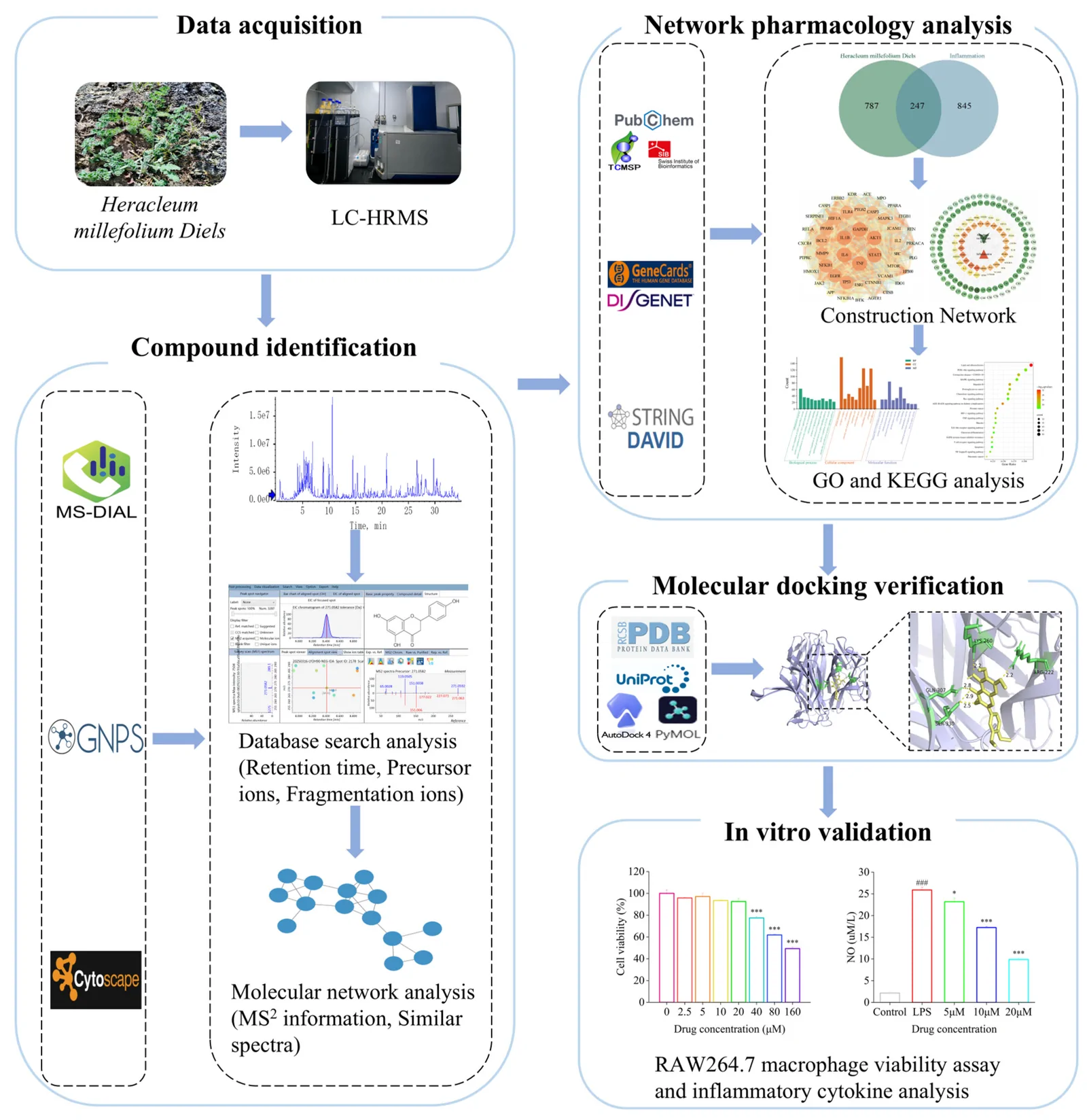
Overall flowchart of this study.

Based on the above considerations, we hypothesized that the combination of comprehensive HRMS-based chemical profiling and computational-experimental validation could facilitate the discovery of bioactive constituents and provide insights into the anti-inflammatory effects of HMD. Accordingly, an integrated workflow combining UPLC-HRMS, “seed”-based molecular networking, network pharmacology, molecular docking, and cellular validation was established in this study. First, the chemical constituents of HMD were comprehensively annotated using UPLC-HRMS coupled with database searching and molecular networking. Subsequently, network pharmacology was applied to prioritize potential bioactive compounds, core targets, and related signaling pathways. Molecular docking was performed to evaluate potential interactions between representative compounds and target proteins. Finally, selected compounds were experimentally validated using in LPS-stimulated RAW264.7 macrophages. The overall workflow is illustrated in Figure 1.

## 2. Materials and Methods

### 2.1. Samples and Reagents

The Tibetan medicinal material HMD was collected from Mozhugongka County, Lhasa, Tibet, China, and authenticated by Professor Qianfeng Gong from Jiangxi University of Chinese Medicine based on morphological characteristics and previously reported literature [19]. A voucher number (No. JXUCM-HMD-2024-001) is currently preserved at the College of Pharmacy, Jiangxi University of Chinese Medicine. Prior to analysis, the samples were ground, passed through a sieve, and stored at room temperature. A representative QC sample was prepared by mixing equal volumes of HMD extracts from six batches to obtain a representative sample for chemical profiling and analytical evaluation. The QC sample was subjected to UPLC-HRMS/MS analysis in triplicate to evaluate compound annotation and analytical reproducibility. For sample preparation, 0.6 g of dried HMD powder was accurately weighed and transferred into a capped centrifuge tube, followed by the addition of 6.0 mL of methanol/water (9:1, *v*/*v*). The mixture was extracted ultrasonically at 40 °C for 1 h and subsequently centrifuged at 13,000 rpm for 10 min. The supernatant was collected for UPLC-HRMS analysis.

Methanol and acetonitrile were purchased from Merck (Darmstadt, Germany) and were of HPLC grade. Ultrapure water was generated using the Milli-Q Synthesis ultrapure water purification system (Millipore Corporation, Burlington, MA, USA). Formic acid and ammonium acetate were purchased from Aladdin (Shanghai, China). Artemitin, velutin, diosmetin-7-O-rutinoside, naringenin, and kaempferol were purchased from Yuan Ye Bio-Technology Co., Ltd. (Shanghai, China). Homoorientin and kaempferol-3-O-glucoside were purchased from Tauto Biotech Co., Ltd. (Shanghai, China), Isorhamnetin-3-O-rutinoside and Citropen were purchased from CATO Research Chemicals Inc. (Guangzhou, China). Kaempferol-3-O-rutinoside was purchased from TanMo Reference Materials Co., Ltd. (Nanjing, China). All compounds had a purity of ≥98.0%. Dulbecco’s Modified Eagle Medium (DMEM), Phosphate-Buffered Saline (PBS), Dimethyl Sulfoxide (DMSO), and Lipopolysaccharide (LPS) were purchased from Solarbio Technology Co., Ltd. (Beijing, China); RAW264.7 cells (ATCC TIB-71; ECACC 91062702) and specific culture medium were purchased from Procell Life Science Technology Co., Ltd. (Wuhan, China); CCK-8 reagent was purchased from Meilun Biotechnology Co., Ltd. (Dalian, China); the NO detection kit was purchased from Beyotime Biotechnology Co., Ltd. (Shanghai, China); and the Tumour Necrosis Factor-α(TNF-α), Interleukin-6(IL-6), and Interleukin-1β(IL-1β) Enzyme Linked Immunosorbent Assay (ELISA) kits were purchased from MultiSciences (Lianke) Biotechnology Co., Ltd. (Hangzhou, China).

### 2.2. UPLC-HRMS Analysis

Sequential windowed acquisition of all theoretical fragment ions (SWATH) isolates precursor ions within predefined mass windows and subsequently acquires MS/MS spectra for all ions within each window and subsequently acquires mixed MS/MS spectra. theoretically enables the acquisition of MS/MS information for all detectable precursor ions [20]. In contrast, information-dependent acquisition (IDA) selects the top N precursor ions from each MS1 scan for sequential fragmentation and MS/MS acquisition [21]. This study compared two data collection methods and selected the optimal strategy for subsequent chemical profiling.

Chromatographic analysis was performed using a Shimadzu 40A LC system (Shimadzu Corporation, Kyoto, Japan) equipped with a ZORBAX RRHD Eclipse Plus C18 column (2.1 × 100 mm, 1.8 μm, Agilent, Santa Clara, CA, USA). The column oven temperature was maintained at 40 °C, the sample tray temperature at 6 °C, with an injection volume of 2 μL and a flow rate of 0.3 mL/min. In positive ion mode, mobile phase A consisted of water containing 0.1% formic acid, and mobile phase B was acetonitrile. The gradient elution programme was as follows: 0–1 min, 5–5% B; 1–5 min, 5–30% B; 5–30 min, 30–95% B; 30–33 min, 95–95% B; 33–33.1 min, 95–5% B; 33.1–35 min, 5–5% B. In negative ion mode, mobile phase A was water containing 5 mM ammonium acetate, and mobile phase B was acetonitrile. The gradient elution programme was as follows: 0–1 min, 2–2% B; 1–18 min, 2–100% B; 18–22 min, 100–100% B; 22–22.1 min, 100–2% B; 22.1–25 min, 2–2% B.

Mass spectrometric analysis was conducted using a Zeno TOF 7600 MS system (AB SCIEX, Marlborough, MA, USA) in both positive and negative ion modes. Information-dependent acquisition (IDA) mode was employed to acquire MS/MS spectra, with nebulizer gas and heater gas pressures both set to 50 psi, and a heater gas temperature of 500 °C. The SWATH acquisition method used the same TOF MS and TOF MS/MS parameters as the IDA method, with the SWATH window configured using 10 variable windows. Specific parameters are detailed in Table S1.

### 2.3. Chemical Composition Identification of HMD

First, MS-DIAL software (version 4.9.221218) was used for peak alignment, feature filtering, and compound annotation. The databases used for MS-DIAL identification included Authentic Standards, MassBank, ReSpect, GNPS, Fiehn HILIC, RIKEN PlaSMA, RIKEN PlaSMA bio-MS/MS, the Fiehn/Vaniya natural product library, and BMDMS-NP. Subsequently, the feature quantification table (CSV file) and MS/MS spectral summary (MSP file) were exported using MS-DIAL and uploaded to the Global Natural Products Social Molecular Networking Platform (GNPS, https://gnps.ucsd.edu (accessed on 24 March 2025)) via the file transfer client WinSCP (https://winscp.net (accessed on 24 March 2025)) for feature-based molecular networking (FBMN) analysis. The GNPS parameter settings were as follows: precursor ion mass tolerance, 0.02 Da; fragment ion mass tolerance, 0.02 Da; minimum pairs cosine, 0.7; minimum matched fragment ions, 6; maximum shift between precursors, 500 Da; network TopK, 10; and maximum connected component Size, 100. These parameters were set based on the instrument’s mass accuracy (≤5 ppm) for tolerance values, while the cosine threshold, fragment ion count, and TopK settings followed GNPS default recommendations to balance spectral matching confidence and network interpretability. The molecular network results were visualised using Cytoscape (version 3.10.0). Finally, using the database search results as seed compounds, the networked compounds were tentatively identified based on the constructed network.

### 2.4. Network Pharmacology

#### 2.4.1. Screening Active Ingredients and Inflammatory Targets

The chemical components of HMD were screened using the TCMSP (https://www.tcmsp-e.com/index.php (accessed on 1 June 2025)) and the SwissADME platform (http://www.swissadme.ch (accessed on 3 June 2025)). The active components were subsequently submitted to the SwissTargetPrediction platform [22] (http://www.swisstargetprediction.ch (accessed on 5 June 2025)) to predict their potential protein targets. Disease targets associated with the keyword “Inflammation” were identified through the DisGeNET (https://disgenet.com/ (accessed on 5 June 2025)), GeneCards (https://www.genecards.org/ (accessed on 5 June 2025)), and OMIM (https://www.omim.org/ (accessed on 5 June 2025)) databases [23]. The active component targets and disease targets obtained were then imported into the Venny 2.1.0 platform (https://bioinfogp.cnb.csic.es/tools/venny/ (accessed on 5 June 2025)) to identify the intersection between HMD active component targets and inflammation-related targets, thereby determining the potential targets for HMD’s anti-inflammatory effects.

#### 2.4.2. Core Target and Component Screening

The identified potential targets were imported into the STRING platform (https://cn.string-db.org/ (accessed on 5 June 2025)) to construct a protein–protein interaction (PPI) network. The PPI network data were subsequently analyzed using Cytoscape software (version 3.10.0), and core targets were screened based on degree, betweenness centrality (BC), and closeness centrality (CC) values. A “drug-component-hub target-disease” network was subsequently constructed in Cytoscape to visualize the relationships among candidate compounds, hub targets, and inflammation. Candidate bioactive compounds associated with the anti-inflammatory activity of HMD were prioritized according to degree values in the compound-target network, and the corresponding network was visualized.

#### 2.4.3. GO and KEGG Enrichment Analysis

The identified targets were imported into the DAVID platform (https://davidbioinformatics.nih.gov/ (accessed on 18 June 2025)) for Gene Ontology (GO) and Kyoto Encyclopedia of Genes and Genomes (KEGG) enrichment analyses, using OFFICIAL_GENE_SYMBOL as the identifier and Homo sapiens as the species. For GO analysis, the top 10 enriched terms in each category, including biological process (BP), cellular component (CC), and molecular function (MF), were selected for visualization. For KEGG analysis, the top 20 enriched pathways were selected for visualization. Visualization is performed using an online bioinformatics analysis and visualization platform (https://www.bioinformatics.com.cn/ (accessed on 20 June 2025)).

### 2.5. Molecular Docking

The top 5 core targets identified above were selected as protein receptors, and the top 4 core components in the “drug-component-core target-disease” network were used as ligands for molecular docking analysis. For receptor preparation, the 3D structures of target proteins were retrieved from the RCSB PDB database. Protein structures were prepared using PyMOL (version 3.1.6.1) by removing water molecules, heteroatoms, and redundant chains. Subsequently, using AutoDockTools-1.5.7, polar hydrogens were added. Chem3D 15.0 was employed to optimize the core component ligands to achieve the lowest energy and optimal structure using the MM2 force field during docking, all rotatable bonds of the ligands were set as flexible, whereas the receptor proteins were treated as rigid. For grid box definition, a blind docking strategy was adopted with the grid box encompassing the entire protein receptor. Grid spacing was set to 1.00 Å. The specific grid box center coordinates and dimensions for each target protein are provided in Table S4. Subsequently, the core component ligands and protein receptors were preprocessed, and pre-file preparation was carried out using AutoDockTools-1.5.7 software. Finally, molecular docking was conducted using AutoDock Vina software, and the results were visualized using PyMOL software.

### 2.6. In Vitro Anti-Inflammatory Evaluation

#### 2.6.1. Cell Culture and Compound Preparation

RAW264.7 cells were cultured in Dulbecco’s Modified Eagle Medium (DMEM) supplemented with 10% fetal bovine serum and 1% penicillin-streptomycin and maintained in a humidified incubator at 37 °C with 5% CO_2_. Kaempferol, naringenin, artemitin, and velutin were dissolved in dimethyl sulfoxide (DMSO) to prepare 20 mM stock solutions and stored for subsequent use. LPS was dissolved in phosphate-buffered saline (PBS) to prepare a 1 mg/mL stock solution.

#### 2.6.2. Cytotoxicity Assay

The 20 mM stock solutions of kaempferol, naringenin, artemitin, and velutin were diluted with Dulbecco’s Modified Eagle Medium (DMEM) to final concentrations of 2.5, 5, 10, 20, 40, 80, and 160 μM. RAW264.7 cells were seeded into 96-well plates at a density of 1 × 10^4^ cells per well and cultured for 24 h. The cells were then treated with the indicated concentrations of each compound for an additional 24 h. Subsequently, 10% (*v*/*v*) Cell Counting Kit-8 (CCK-8) reagent was added to each well, followed by incubation for 1 h. Cell viability was determined by measuring the absorbance at 450 nm using a microplate reader.

#### 2.6.3. NO and Inflammatory Marker Assays

Control, model, and treatment groups were established. Treatment concentrations were selected based on the results of the cytotoxicity assay to ensure non-cytotoxic conditions. RAW264.7 cells were seeded into 96-well plates at a density of 1 × 10^4^ cells per well and cultured for 24 h. The cells were then treated with the indicated compounds according to the experimental groups. After 2 h of drug treatment, LPS was added to induce inflammation at a final concentration of 1 μg/mL. Following 24 h of incubation, cell culture supernatants were collected. The levels of nitric oxide (NO), tumor necrosis factor-α (TNF-α), interleukin-1β (IL-1β), and interleukin-6 (IL-6) were quantified using commercially available NO assay kits and enzyme-linked immunosorbent assay (ELISA) kits according to the manufacturers’ instructions.

#### 2.6.4. Statistical Analysis

All experiments were performed independently at least three times. Data are presented as mean ± standard deviation (SD). Statistical differences among groups were analyzed using one-way analysis of variance (ANOVA), followed by Dunnett’s multiple comparisons test. Statistical analyses were performed using IBM SPSS Statistics 27.0 software. Figures and graphs were generated using Origin 2024 software. A *p*-value < 0.05 was considered statistically significant.

## 3. Results

### 3.1. Chemical Composition of HMD

#### 3.1.1. Comparison of IDA and SWATH Acquisition Modes

To determine the most appropriate MS/MS acquisition strategy, we analyzed HMD samples using both IDA and SWATH modes. As shown in Figure S1, the number of features in the GNPS network was 11,344 in IDA mode and 10,649 in SWATH mode. Moreover, a total of 472 compounds were identified or tentatively identified through database searching and GNPS analysis in the IDA mode, whereas only 283 compounds were identified or tentatively identified in the SWATH mode. or tentatively identified.

#### 3.1.2. Identification of the Chemical Composition of HMD

By integrating MS1 and MSMS database searching with GNPS molecular networking analysis, a total of 472 chemical constituents were identified and putatively identified from HMD (Table S2). The identified constituents mainly comprised flavonoids and their derivatives (127), coumarins and their derivatives (56), benzene and substituted derivatives (38), carbohydrates and carbohydrate conjugates (32), lignans and their derivatives (8), glycerophospholipids (34), glycerolipids (14), carboxylic acids and their derivatives (74), steroids and their derivatives (6), cinnamic acid derivatives (4), terpenoids and their derivatives (47), and others (32). Among these 472 compounds, 10 were positively identified (Level 1), 376 were tentatively identified based on database searching (Level 2), and 86 were tentatively identified based on molecular networking (Level 3).

##### Putative Identification Through Database Search

To further illustrate the compound identification process, representative compounds from major chemical classes were selected for detailed analysis.

Flavonoids and Their Derivatives.

**Figure 2:**
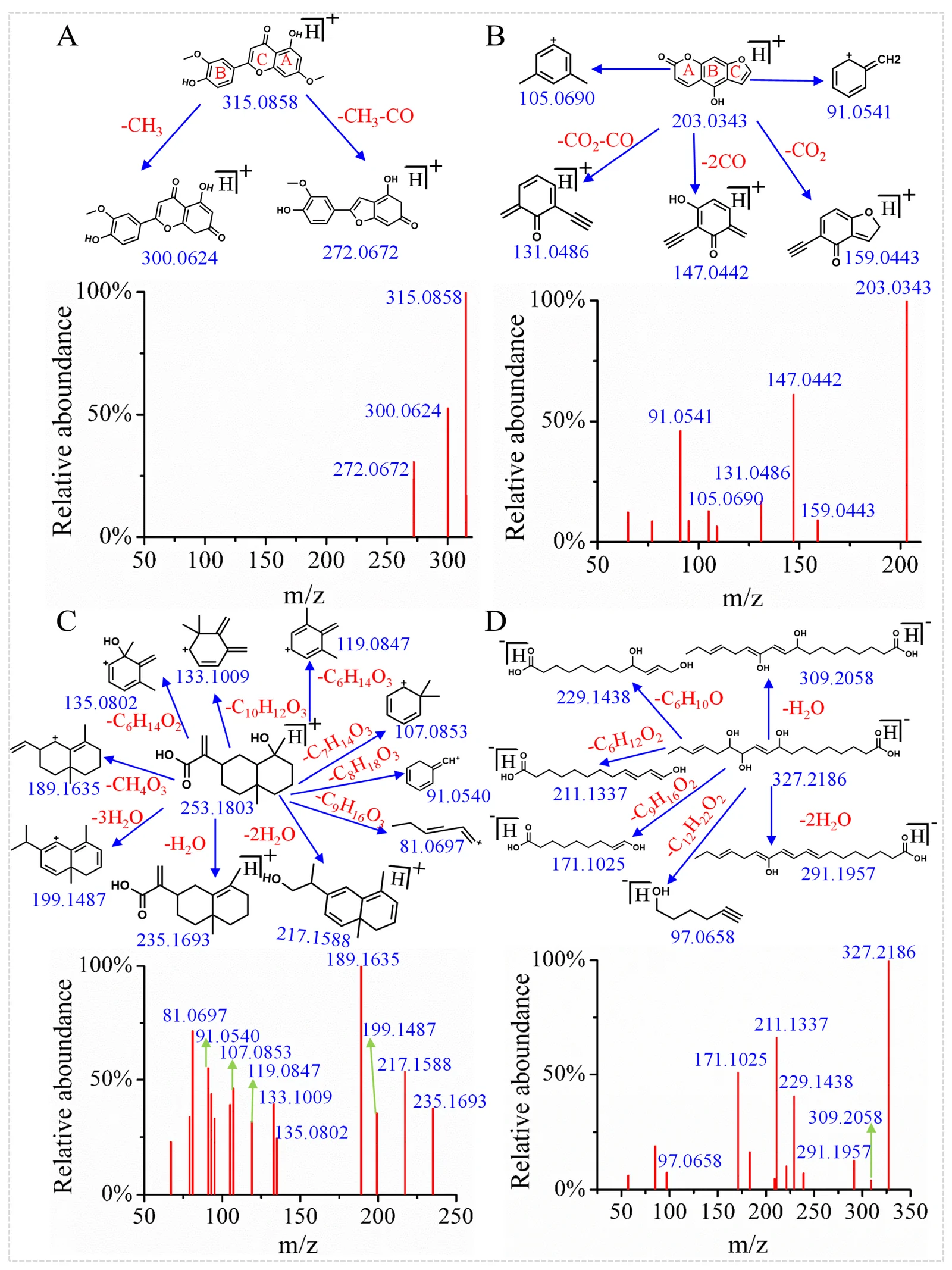
Fragment structures and MS/MS of the compounds. (**A**) Velutin. (**B**) Bergaptol. (**C**) 2-(8-hydroxy-4A,8-dimethyl-1,2,3,4,5,6,7,8A-octahydronaphthalen-2-Yl) prop-2-enoic acid. (**D**) 9,12,13-trihydroxyoctadeca-10,15-dienoic acid.

In this study, a total of 127 flavonoids and their derivatives were identified. Flavonoids primarily undergo retro-Diels-Alder (RDA) cleavage, accompanied by neutral losses of H_2_O, CO, and CH_3_. As illustrated in Figure 2A, compound 37 (tR = 12.42 min) generated a protonated molecular ion at *m*/*z* 315.0858, corresponding to the molecular formula C_17_H_14_O_6_. The fragment ion at *m*/*z* 300.0624 ([M+H-CH_3_]^+^) was produced by the loss of a CH_3_, whereas the fragment ion at *m*/*z* 272.0672 resulted from the sequential loss of CH_3_ and CO ([M+H-CH_3_-CO]^+^). Based on fragmentation pattern analysis and comparison with the MassBank database, compound 37 was tentatively identified as velutin and subsequently confirmed using an authentic reference standard.

Coumarins and Their Derivatives.

In this study, a total of 56 coumarins and their derivatives were identified. Their major fragmentation pathways mainly involved the neutral loss of CO and CO_2_. Compound 276 (tR = 17.40 min) generated a protonated molecular ion [M+H]^+^ at *m*/*z* 203.0343, corresponding to the molecular formula C_11_H_6_O_4_. As shown in Figure 2B, the loss of CO_2_ produced a fragment ion at *m*/*z* 159.0443, whereas the sequential loss of two CO molecules generated a fragment ion at *m*/*z* 147.0442. The characteristic fragment ion at *m*/*z* 131.0486 was attributed to the combined loss of CO and CO_2_. In addition, fragment ions at *m*/*z* 105.0690 and *m*/*z* 91.0541 were observed. Based on database searches and fragmentation behavior analysis, compound 276 was preliminarily identified as bergaptol.

Terpenoids and Their Derivatives.

In this study, a total of 47 terpenoids and their derivatives were identified from HMD. Compound 416 (tR = 11.54 min) generated a protonated molecular ion at *m*/*z* 253.1803, corresponding to the molecular formula C_15_H_24_O_3_, and was preliminarily identified as 2-(8-hydroxy-4a,8-dimethyl-1,2,3,4,5,6,7,8a-octahydronaphthalen-2-yl) prop-2-enoic acid. As shown in Figure 2C, the sequential loss of H2O molecules produced fragment ions at 235.1693 ([M+H-H_2_O]^+^), 217.1588 ([M+H-2H_2_O]^+^), and 199.1487 ([M+H-3H_2_O]^+^). In addition, fragment ions at *m*/*z* 135.0802 ([M+H-C_6_H_14_O_2_]^+^) and 119.0847 ([M+H-C_6_H_14_O_3_]^+^) were generated through C-C bond cleavage of the saturated six-membered ring. Furthermore, fragment ions at *m*/*z* 33.1009 ([M+H-C_10_H_12_O_3_]^+^), 107.0853 ([M+H-C_7_H_14_O_3_]^+^), 91.0540 ([M+H-C_8_H_18_O_3_]^+^), and 81.0697 ([M+H-C_9_H_16_O_3_]^+^) were also observed.

Carboxylic Acids and Their Derivatives.

A total of 74 carboxylic acids and their derivatives were identified, including fatty acids, benzoic acid, and amino acids. Fatty acids, particularly hydroxy fatty acids, are prone to losing H_2_O and fatty chains during fragmentation. In the negative ion mode, compound 231 (tR = 7.16 min) generated a deprotonated molecular ion at *m*/*z* 327.2186, corresponding to the molecular formula C_18_H_32_O_5_, and was preliminarily identified as 9,12,13-trihydroxyoctadeca-10,15-dienoic acid through database searching. As shown in Figure 2D, the sequential loss of H_2_O generated fragment ions at *m*/*z* 309.2058 ([M-H-H_2_O]^−^) and 291.1957 ([M-H-2H_2_O]^−^). In addition, fragment ions at *m*/*z* 229.1438 ([M-H-C_6_H_10_O]^−^) and 211.1337 ([M-H-C_6_H_12_O_2_]^−^) were formed through the loss of neutral fragments. Fragment ions at m/z 171.1025 ([M-H-C_9_H_16_O_2_]^−^) and 97.0658 ([M-H-C_12_H_22_O_2_]^−^) were also detected.

##### Putative Identification Through “Seed”-Based Molecular Networking

To facilitate propagation-based identification, a molecular network was constructed according to MS/MS spectral similarity. The resulting molecular network is presented in Figure S2. Molecular network analysis revealed that coumarins and their derivatives, flavonoids and their derivatives, glycerophospholipids, terpenoids and their derivatives, glycerides, carboxylic acids and their derivatives, and nitrogen-containing organic compounds each formed distinct molecular clusters. The positive ion dataset contained 6277 precursor ions, which organized into 263 clusters containing two or more nodes, together with 4196 singleton nodes (Figure S2A). Meanwhile, the negative ion dataset comprised 5481 precursor ions, forming 185 clusters containing two or more nodes and 4234 singleton nodes (Figure S2B).

**Figure 3:**
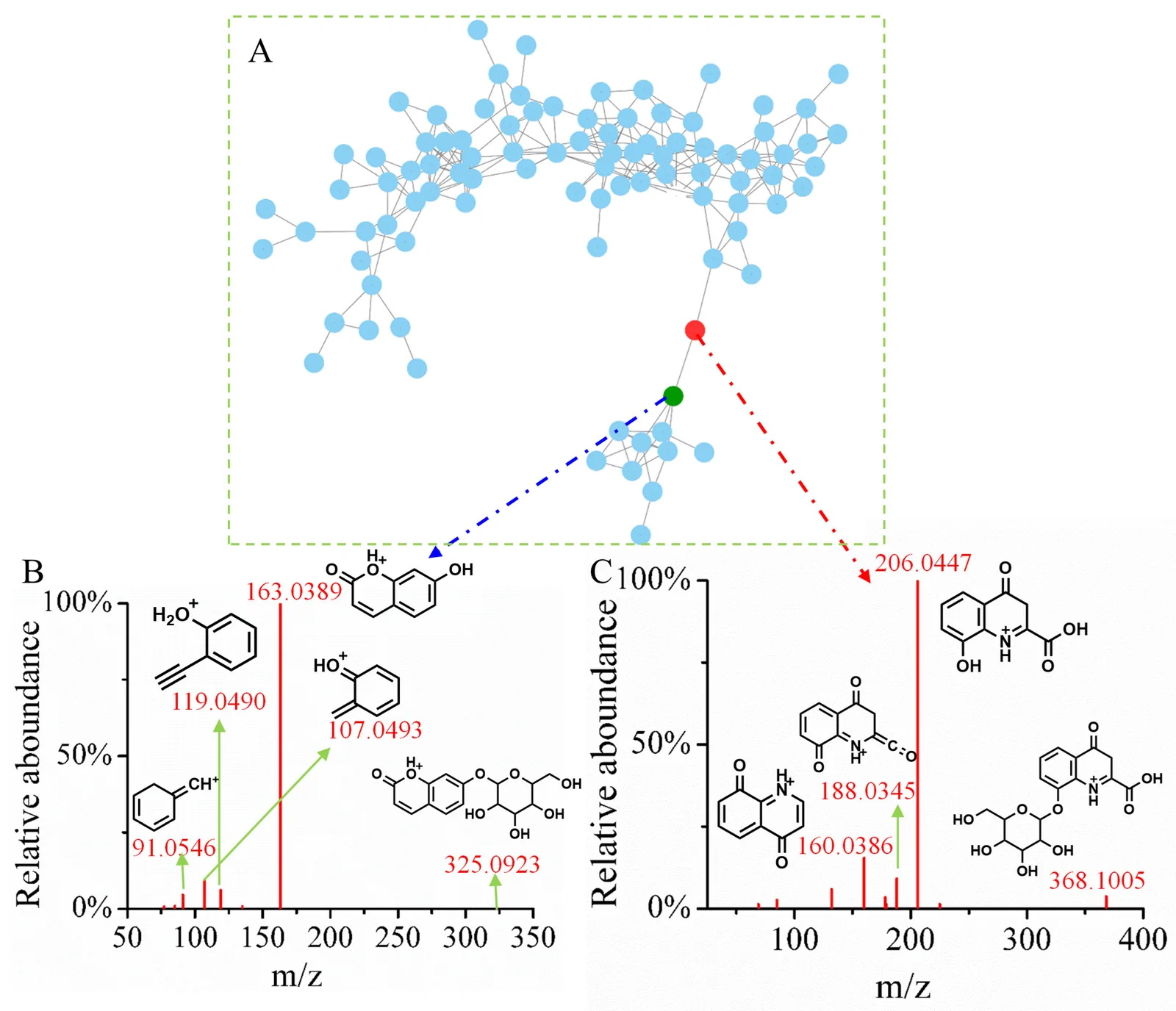
Example of compound structure identification by “seed”-based molecular networking. (**A**) Coumarin-Related Clusters. (**B**) “Seed” compound skimmin (**C**) The inferred compound 4-oxo-8-[3,4,5-trihydroxy-6-(hydroxymethyl)oxan-2-yl]oxy-3H-quinoline-2-carboxylic acid.

Because GNPS automatic identification relies primarily on its built-in spectral libraries, compounds without direct library matches require further characterization using structurally related known compounds (“seed” compounds). In this study, seed compounds were obtained through MS-DIAL and GNPS database searches. To illustrate the workflow of “seed”-based molecular networking, a representative coumarin-related cluster was selected as an example for structural identification (Figure 3A). Based on database searching results, the green-labeled node (tR = 3.85 min, *m*/*z* = 325.0923) was preliminarily identified as skimmin, and its corresponding fragmentation pattern is shown in Figure 3B. Since this compound belonged to the coumarin class, the structurally connected nodes within the same cluster were also considered likely to be coumarin derivatives. Therefore, for unidentified compounds (e.g., the red-labeled node), candidate structures were first retrieved from the COCONUT database based on MS1 information, followed by class-directed structural filtering according to the classification of the seed compound. Using this strategy, the red-labeled node was tentatively identified 4-oxo-8-[3,4,5-trihydroxy-6-(hydroxymethyl)oxan-2-yl]oxy-3H-quinoline-2-carboxylic acid, and its proposed fragmentation pathway is illustrated in Figure 3C.

### 3.2. Network Pharmacology Analysis

#### 3.2.1. Prediction of Potential Targets Associated with the Anti-Inflammatory Effects of HMD

Based on the chemical profiling results of HMD, potential active constituents were screened according to the criteria described in Section 2.4.1. A total of 94 active constituents were retained after screening (Table S3). The SMILES strings of these compounds were subsequently imported into the SwissTargetPrediction platform to predict their potential target proteins. After removing duplicate entries, a total of 1034 unique target proteins were obtained.

#### 3.2.2. Protein–Protein Interaction (PPI) Network Construction and Core Target Screening

**Figure 4:**
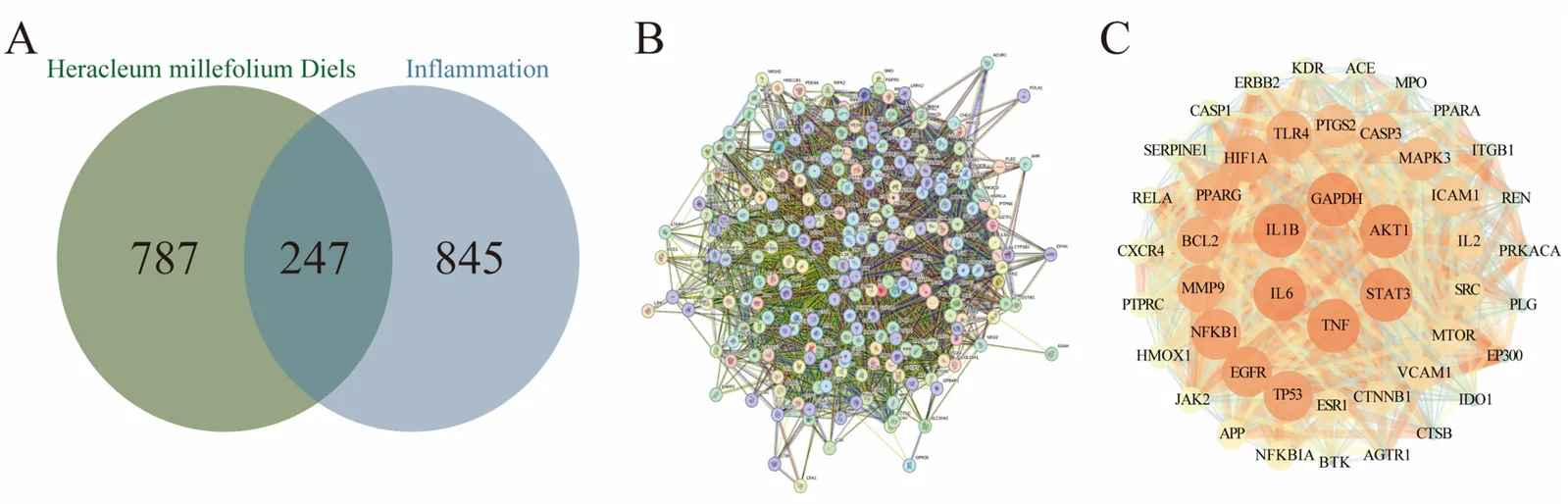
(**A**) Venn diagram of HMD and inflammatory target genes. (**B**) PPI network diagram of genes from the STRING database intersection. (**C**) Core target diagram constructed using Cytoscape software, in which node size and color are related to importance in the network.

A total of 1092 inflammation-related targets were collected from the DisGeNET, GeneCards, and OMIM databases. The predicted targets of the active constituents and inflammation-related targets were imported into the Venny 2.1.0 platform to generate a Venn diagram, which identified 247 overlapping targets (Figure 4A). These common targets were subsequently uploaded to the STRING database to construct a protein–protein interaction (PPI) network (Figure 4B). Cytoscape 3.10.1 software was then employed for network visualization and topological analysis. The CytoNCA plugin was used to calculate network topological parameters, and the mean values of Degree, BC, and CC were used as cutoff thresholds for core target screening. Targets with degree values >50.6316, betweenness centrality (BC) values >211.0769, and closeness centrality (CC) values >0.0022 were selected as core targets, resulting in a total of 47 key targets (Figure 4C). These targets may be associated with the potential anti-inflammatory activity of HMD.

#### 3.2.3. Construction of the Drug-Component-Core Target-Disease Network and Screening of Core Constituents

**Figure 5:**
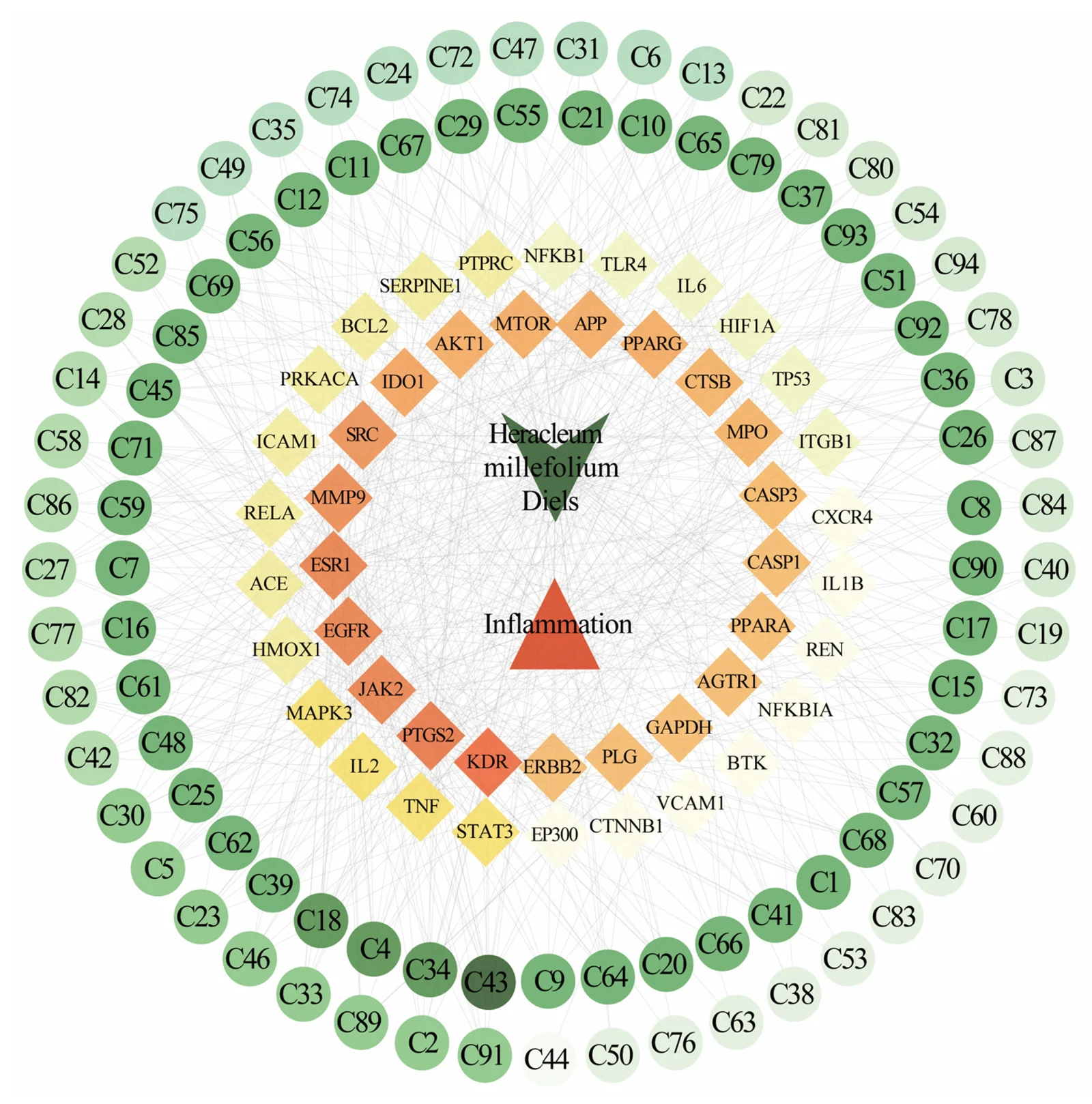
Drug-component-core target-disease network diagram.

A compound-component-core target-disease interaction network was constructed using Cytoscape 3.10.1 software (Figure 5). To prioritize candidate compounds for subsequent experimental evaluation, we ranked according to their degree centrality values within the network. Based on the distribution of degree values, a cut-off threshold of degree ≥10 was applied, retaining the top-ranked compounds with the highest connectivity to anti-inflammatory targets: velutin, artemitin, kaempferol, and naringenin. In addition, each target node was connected to multiple constituent nodes, suggesting that the potential anti-inflammatory activity of HMD may involve interactions among multiple compounds and targets.

#### 3.2.4. GO and KEGG Enrichment Analysis

**Figure 6:**
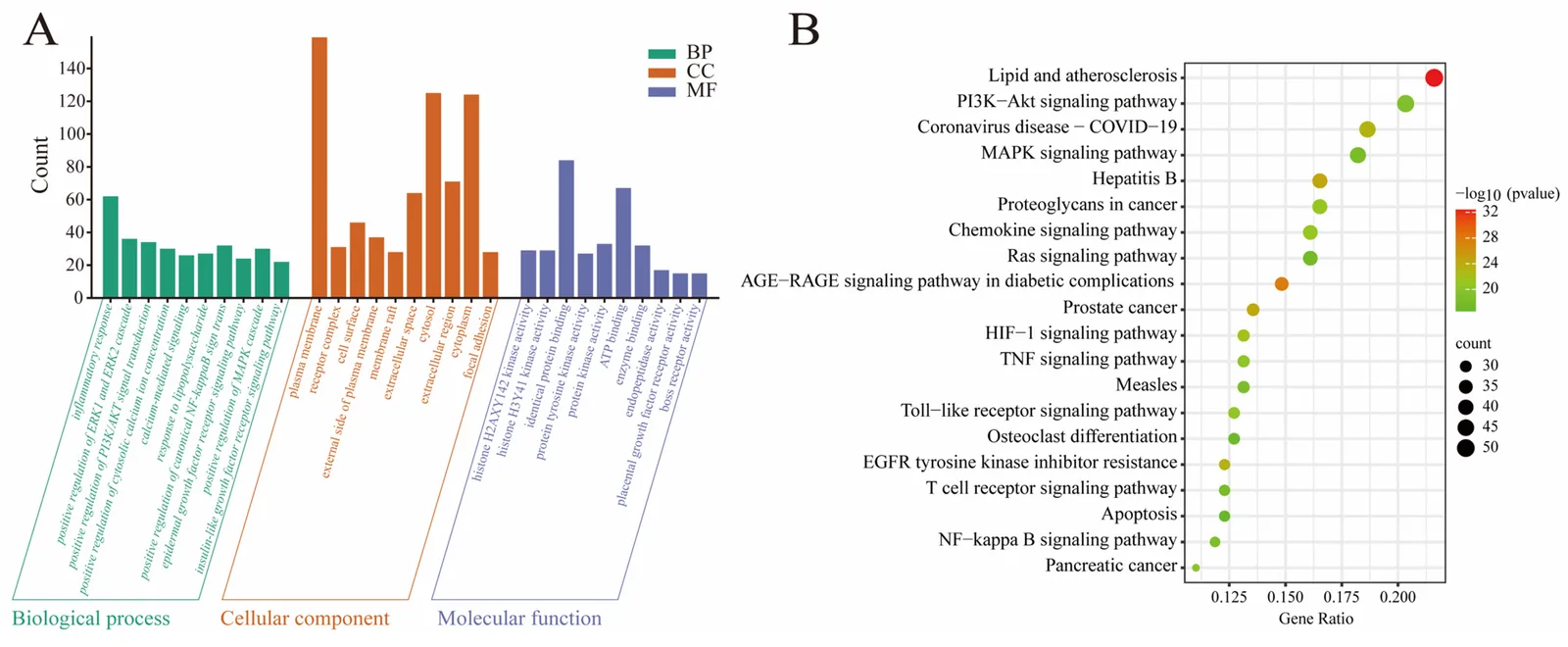
GO and KEGG enrichment analysis. (**A**) GO functional enrichment analysis bar chart. (**B**) Bubble chart showing the enrichment of 20 pathways according to KEGG analysis.

The predicted targets were imported into the DAVID database for Gene Ontology (GO) and Kyoto Encyclopedia of Genes and Genomes (KEGG) enrichment analyses. A total of 1455 GO terms were enriched, and the top 10 enriched terms in each category were selected for visualization (Figure 6A). Among these, the biological process (BP) category included 1084 enriched terms, with major terms related to inflammatory response, positive regulation of the ERK1/ERK2 cascade, and positive regulation of PI3K/AKT signaling. The cellular component (CC) category contained 120 enriched terms, including “plasma membrane”, “receptor complex”, and “cell surface”. The molecular function (MF) category comprised 251 enriched terms, such as “histone H2AXY142 kinase activity”, “histone H3Y41 kinase activity”, and “identical protein binding”.

KEGG pathway enrichment analysis identified 173 significantly enriched pathways associated with the predicted targets. The top 20 enriched pathways were selected for visualization according to ascending *p*-value order (Figure 6B). The results suggested that the anti-inflammatory effects of HMD may be associated with the regulation of multiple signaling pathways, particularly the lipid and atherosclerosis pathway, the AGE-RAGE signaling pathway in diabetic complications, and the hepatitis B pathway.

### 3.3. Molecular Docking Analysis

**Figure 7:**
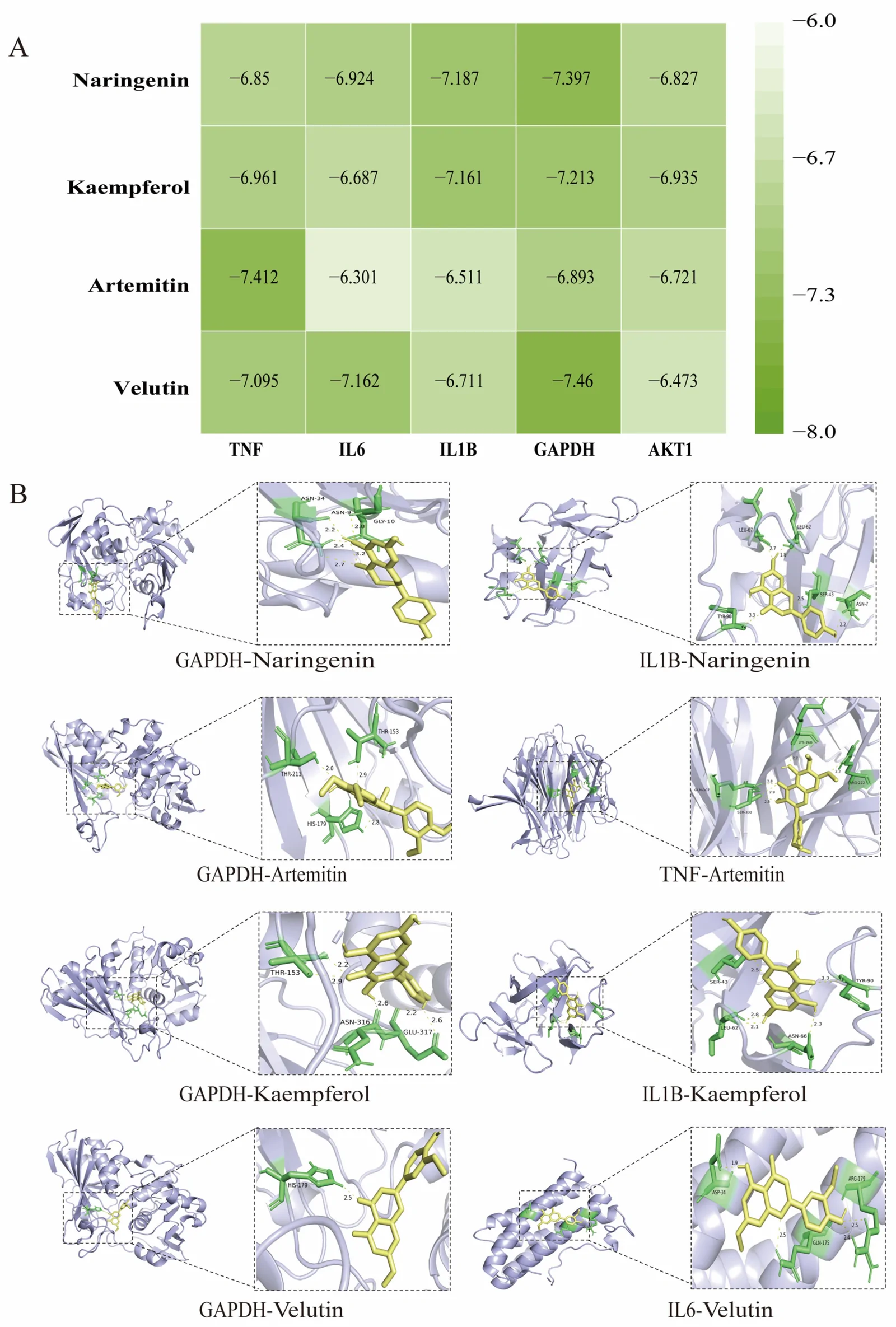
Molecular docking results. (**A**) Heat map of molecular docking binding energies between core targets and core components of HMD. (**B**) Visualization of docking between selected core targets and core components.

The five core targets identified from the network analysis, namely TNF, IL-6, IL-1β, Glyceraldehyde-3-Phosphate Dehydrogenase (GAPDH), and AKT Serine/Threonine Kinase 1(AKT1), were selected as receptor proteins, while the four principal active constituents identified from the compound-target-disease network, including velutin, artemitin, kaempferol, and naringenin, were selected as ligands. Molecular docking analysis was subsequently performed using AutoDock Vina. The docking results showed that all ligand-target complexes exhibited binding energies below −5.0 kcal/mol, suggesting potential binding interactions between the active constituents and core targets (Figure 7A). Among these complexes, eight exhibited binding energies lower than −7.0 kcal/mol, indicating relatively stronger predicted binding affinitie. To further investigate the ligand-target interactions, PyMOL software was employed for visualization analysis of the representative docking conformations with relatively high binding affinities (Figure 7B). For instance, velutin formed a hydrogen bond interaction with the HIS179 residue of GAPDH, with a binding energy of −7.46 kcal/mol. Artemitin formed multiple hydrogen bond interactions with the GLN307, SER330, LYS260, and ARG222 residues of TNF, exhibiting a binding energy of −7.41 kcal/mol. These results provide computational evidence supporting potential interactions between the selected compounds and inflammation-related targets.

### 3.4. Results of In Vitro Experiments

#### 3.4.1. Cytotoxicity Evaluation of the Selected Compounds

**Figure 8:**
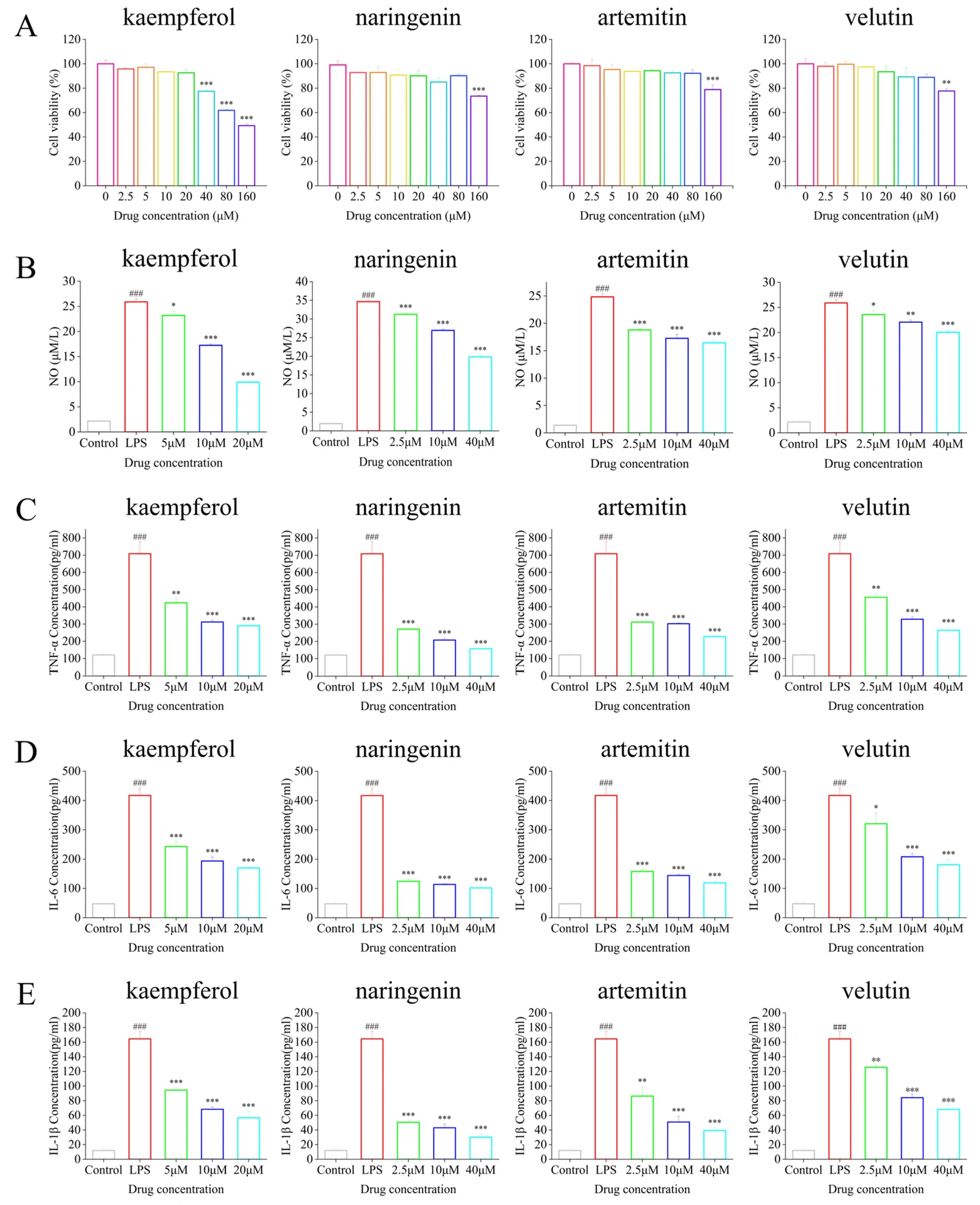
Anti-inflammatory effects of the four active components of HMD. (**A**) Cytotoxic effects of different concentrations of the four active components on RAW264.7 cells. ** *p* < 0.01,*** *p* < 0.001 vs. control group. (**B**–**E**) Effects of the four active components on the secretion of NO, TNF-α, IL-6, and IL-1β by LPS-stimulated RAW264.7 cells. ^###^ *p* < 0.001 vs. control group, * *p* < 0.05, ** *p* < 0.01, *** *p* < 0.001 vs. LPS group.

The cytotoxic effects of the selected compounds on RAW264.7 cells were evaluated, and the results are presented in Figure 8A. Naringenin, artemitin, and velutin exhibited no significant effects on cell viability at concentrations up to 80 μM compared with the control group, indicating no apparent cytotoxicity within this concentration range. However, significant reductions in cell viability were observed at 160 μM. In contrast, kaempferol showed no apparent cytotoxicity at concentrations up to 20 μM, whereas a significant decrease in cell viability was detected at concentrations of 40 μM and higher. Based on these findings, non-cytotoxic concentration ranges were selected for subsequent anti-inflammatory experiments.

#### 3.4.2. Anti-Inflammatory Effects of Active Constituents in LPS-Induced RAW264.7 Cells

Based on the cytotoxicity evaluation, naringenin, artemitin, and velutin were tested at concentrations of 2.5, 10, and 40 μM, while kaempferol was evaluated at concentrations of 5, 10, and 20 μM. As shown in Figure 8B, LPS stimulation significantly increased NO production compared with the control group. Treatment with kaempferol, naringenin, artemitin, and velutin markedly reduced NO levels in LPS-stimulated RAW264.7 cells. Moreover, all four compounds exhibited concentration-dependent inhibitory effects on NO production. As illustrated in Figure 8C-E, LPS stimulation significantly elevated the secretion levels of pro-inflammatory cytokines, including TNF-α, IL-6, and IL-1β. Compared with the LPS model group, treatment with the four compounds significantly suppressed the production of these cytokines. Similar to the NO inhibition results, a concentration-dependent anti-inflammatory trend was observed. These findings suggested that velutin, artemitin, kaempferol, and naringenin exerted significant anti-inflammatory activity in LPS-induced RAW264.7 macrophages.

## 4. Discussion

Inflammation is a critical physiological response to tissue injury and infection, while persistent inflammation may contribute to various chronic diseases [24]. Although HMD has been traditionally used for inflammatory disorders and exhibits multiple biological activities, its chemical basis and potential anti-inflammatory constituents remain insufficiently understood [5]. In the present study, IDA and SWATH MS/MS acquisition modes were compared, and IDA showed better performance in generating GNPS molecular networking features and compound annotations. Using the optimized strategy, UPLC-HRMS combined with database searching and seed-based molecular networking enabled the identification of 472 compounds at different confidence levels, spanning diverse chemical classes such as flavonoids, coumarins, organic acids, terpenoids, and glycerophospholipids, thereby providing a comprehensive overview of the chemical composition of HMD. Recent studies on other *Heracleum* species have provided important insights into the chemical diversity and characteristic metabolites of this genus [25]. Consistent with previous reports, flavonoids, coumarins, and terpenoids represented major annotated metabolite classes in HMD. Compared with coumarin-rich European *Heracleum* species, HMD showed a greater representation of flavonoid constituents [26], while the detection of glycerophospholipids and other polar metabolites further highlighted its distinct chemical features. These species-specific metabolic characteristics warrant further comparative and functional investigation.

Based on the compound-target-disease network analysis, four potential anti-inflammatory constituents, namely velutin, artemitin, kaempferol, and naringenin, were identified as potential key contributors to the anti-inflammatory activity of HMD. In addition, PPI network analysis demonstrated extensive interactions between the active constituents and inflammation-related target proteins. According to the topological parameters of the network, TNF, IL-6, IL-1β, GAPDH, and AKT1 were identified as the five core targets. Previous studies have demonstrated that TNF can suppress TGF-β signaling through epigenetic regulation and the RBP-J pathway, thereby amplifying pro-inflammatory responses [27]. Agam et al. reported that aspirin alleviates LPS-induced inflammatory responses by inhibiting the expression of IL-6 and TNF-α [28]. In addition, IL-1β, GAPDH, and AKT1 have also been widely recognized as important regulatory factors involved in inflammatory diseases [29,30,31]. These findings suggested that the potential anti-inflammatory activity of HMD may involve multiple constituents and targets rather than a single compound-target interaction.

GO and KEGG enrichment analyses suggested that the potential anti-inflammatory effects of HMD may be associated with the regulation of multiple inflammation-related signaling pathways, including ERK, PI3K/AKT, AGE-RAGE, and other inflammatory pathways. Among these pathways, the AGE-RAGE signaling pathway showed relatively high enrichment significance in the KEGG analysis and was therefore selected as a representative pathway for further discussion. However, this selection does not indicate that AGE-RAGE signaling functions independently of or is more important than other classical inflammatory pathways, such as NF-κB and MAPK signaling pathways. Instead, the AGE-RAGE pathway was discussed as a potential regulatory pathway predicted by network pharmacology within a broader inflammatory signaling network. Previous studies have shown that the MAPK/ERK signaling pathway plays a crucial role in rheumatoid arthritis progression by promoting the production of pro-inflammatory cytokines and modulating inflammatory responses [32]. Huang et al. demonstrated that cynaroside alleviated intestinal inflammatory injury by modulating the PI3K/AKT signaling pathway and maintaining intestinal barrier integrity [33]. Singh et al. further reported that inhibition of the PI3K/AKT signaling pathway or the TLR2 receptor effectively suppressed the inflammatory response induced by Desulfovibrio vulgaris [34]. In addition, AGE-RAGE activation has been reported to regulate downstream inflammatory cascades, including MAPK/NF-κB signaling, thereby contributing to inflammation, apoptosis, autophagy, and endoplasmic reticulum stress [35]. In addition, KEGG enrichment analysis also identified several classical inflammation-related pathways, including the HIF-1 signaling pathway [36]. Collectively, these enrichment results, together with the core targets identified from the PPI network, suggest that the potential anti-inflammatory activity of HMD may involve coordinated regulation of multiple inflammation-associated pathways rather than a single signaling pathway. Further experimental validation is required to confirm the involvement of these predicted pathways.

Molecular docking showed that all ligand-target complexes had binding energies below −5.0 kcal/mol, and several complexes exhibited binding energies lower than −7.0 kcal/mol, suggesting favorable predicted ligand-target interactions in silico. These findings supported the existence of potential interactions between the core constituents and inflammation-related targets at the molecular level, providing additional computational support for the predicted compound-target associations. Previous studies have also reported the anti-inflammatory activities of these compounds. Velutin suppresses NF-κB-mediated osteoclast differentiation and reduces HIF-1α expression [37]. Kaempferol has been reported to alleviate endoplasmic reticulum stress-associated inflammation and hyperglycemia, thereby exhibiting potential protective effects [38]. Artemitin effectively ameliorates airway inflammation, oxidative stress, and mitochondrial dysfunction in asthma through regulation of the ABCG2/RAB7A signaling axis and inhibition of PANoptosis [39]. Moreover, naringenin significantly inhibited ear edema in arachidonic acid- and phorbol ester-induced inflammatory models, suggesting its potential anti-inflammatory activity in experimental models [40]. Collectively, these findings support the reliability of the present screening results and suggest that these four constituents may represent potential bioactive components associated with the anti-inflammatory activity of HMD that warrant further investigation.

However, network pharmacology and molecular docking are essentially computational prediction approaches and cannot directly confirm anti-inflammatory activities of candidate compounds. Therefore, further biological validation through cellular experiments was necessary. In the present study, an LPS-stimulated RAW264.7 macrophage model was selected because it is a widely used and reliable in vitro model for evaluating the anti-inflammatory activities of natural products through the induction of inflammatory mediators. Macrophages are key innate immune cells that play essential roles in immune regulation, inflammatory responses, and tissue repair [41]. NO, TNF-α, IL-6, and IL-1β are important inflammatory mediators involved in the initiation and amplification of inflammatory responses [42,43,44,45]. The anti-inflammatory activities of the selected compounds were evaluated by measuring these inflammatory mediators in LPS-stimulated RAW264.7 cells. All four compounds significantly reduced the levels of NO, TNF-α, IL-6, and IL-1β, with dose-dependent inhibitory effects observed. These findings indicate that the selected compounds exhibit anti-inflammatory effects in this cellular model and suggest their potential contribution to the observed biological activity of HMD. However, the results obtained from a single cell model cannot fully represent the complexity of human inflammatory responses. Further validation using additional cellular models, such as THP-1-derived macrophages or primary macrophages, as well as appropriate in vivo inflammatory models, is required to further confirm the anti-inflammatory effects and elucidate their pharmacological relevance. Importantly, the present findings are also consistent with previous experimental studies demonstrating that diverse medicinal and edible plants possess significant anti-inflammatory activity in vivo. Similar reductions in inflammatory responses have been reported for *Cicer arietinum* L. and different cultivars of *Capsicum annuum* L. using established rodent models, suggesting that chemically diverse phytoconstituents may converge on common inflammatory pathways despite differences in botanical origin [46,47]. These observations further support the potential role of plant-derived metabolites as multi-target modulators of inflammation.

Taken together, this study established an integrated strategy combining UPLC-HRMS, database searching, seed-based molecular networking, network pharmacology, molecular docking, and cellular validation to characterize the chemical composition and investigate the potential anti-inflammatory activity of HMD. Nevertheless, several limitations should be acknowledged. Network pharmacology and molecular docking are predictive approaches that rely on available databases and computational algorithms; therefore, the predicted targets and pathways should be regarded as mechanistic hypotheses rather than definitive evidence of pathway regulation. Although the selected compounds showed anti-inflammatory effects in LPS-stimulated RAW264.7 macrophages, further validation of the underlying mechanisms is required. The involvement of key inflammatory regulators and pathways, including NF-κB, iNOS, COX-2, MAPKs, and AKT signaling, should be confirmed using approaches such as Western blotting, RT-qPCR, phospho-protein analysis, and appropriate in vivo models. In addition, the potential synergistic, additive, or antagonistic interactions among velutin, artemitin, kaempferol, and naringenin remain unexplored and warrant further investigation using combination models. Furthermore, future translational studies should evaluate the bioavailability, pharmacokinetic properties, metabolism, toxicity, herb-drug interactions, formulation strategies, and dose conversion of HMD-derived constituents. Future research should focus on identifying additional bioactive constituents, further validating their pharmacological mechanisms, and systematically evaluating their individual and combined biological effects.

## 5. Conclusions

This study employed UPLC-HRMS technology combined with database searching and “seed”-based molecular networking to systematically analyze the chemical composition of HMD. The results showed that a total of 472 components were identified or tentatively identified in HMD, including 386 identified through database searching and 86 identified using “seed”-based molecular networking, achieving high coverage identification. Based on this, combined with network pharmacology and molecular docking, potential anti-inflammatory targets and signaling pathways associated with HMD were predicted, suggesting that the anti-inflammatory activity of HMD may involve coordinated regulation of multiple targets and signaling pathways by diverse bioactive constituents. Finally, in vitro experiments demonstrated the anti-inflammatory effects of the selected compounds in LPS-stimulated RAW264.7 macrophages. This study not only fills the gap in the research on the anti-inflammatory mechanism of HMD but also provides a foundation for further investigation and potential development of HMD-derived bioactive constituents.

## Data Availability

All data are contained within the article and Supplementary Materials. Further inquiries can be directed to the corresponding author.

## Supplementary Materials

The following supporting information can be downloaded at: https://www.mdpi.com/article/10.3390/metabo16080573/s1, Figure S1: Comparison of IDA and SWATH acquisition methods in UPLC-HRMS. Table S1: Data-Dependent Acquisition parameters. Figure S2: Molecular network of HMD. (A) Positive ion mode. (B) Negative ion mode. Table S2: Detailed information of 472 compounds identified by searching databases and “seed”-based molecular networking. Table S3: Detailed information on 94 active ingredients screened by TCMSP and Swiss ADME. Table S4: The Molecular Docking Grid Boxes Parameters.

## Abbreviations

The following abbreviations are used in this manuscript:

AKT1	AKT Serine/Threonine Kinase 1
BP	Biological Process
CC	Cellular Component
DL	Drug-Likeness
ELISA	Enzyme Linked Immunosorbent Assay
FBMN	feature-based molecular networking
GAPDH	Glyceraldehyde-3-Phosphate Dehydrogenase
GNPS	Global Natural Products Social Molecular Networking
GO	Gene Ontology
IL-1β	Interleukin-1β
IL-6	Interleukin-6
KEGG	Kyoto Encyclopedia of Genes and Genomes
LPS	Lipopolysaccharide
MF	Molecular Function
OB	Oral Bioavailability
PBS	Phosphate-Buffered Saline
PPI	Protein–Protein Interaction
TNF-α	Tumour Necrosis Factor-α
UPLC-HRMS	Ultra-performance Liquid Chromatography–High-Resolution Mass Spectrometry
