# Supplementary material for: Chemical Profiling and Anti-Inflammatory Mechanisms of Heracleum millefolium Diels Revealed by Molecular Networking, Network Pharmacology, and Cellular Validation
Source: Metabolites. 2026 Aug 13;16(8):573. doi: 10.3390/metabo16080573 (PMC13515270; doi:10.3390/metabo16080573)
Supplement: Supplementary file 1 [file metabolites-16-00573-s001.zip › metabolites-4469398-supplementary.pdf]

Chemical Profiling and Anti-inflammatory Mechanisms of Heracleum millefolium Diels Revealed by Molecular Networking, Network Pharmacology, and Cellular Validation

Genhua Zhu [1], Xueqin Yin [1], Ciren Dunzhu [2], Xiang Zhou [1], Meijuan Shao [3], En Yuan [3], Zhihong Yan [3,*] and Xiaoyu Xie [3,*]

1 Key Laboratory of Modern Preparation of Traditional Chinese Medicine, Ministry of Education, Jiangxi University of Chinese Medicine, Nanchang 330100, China; 20020688@jxutcm.edu.cn (G.Z.); yinxueqin@jxutcm.edu.cn (X.Y.); 20121011@jxutcm.edu.cn (X.Z.)
2 Department of Traditional Chinese Medicine, University of Tibetan Medicine, Lhasa 850000, China; 2003001@ttmc.edu.cn
3 Department of Pharmacy, Jiangxi University of Chinese Medicine, Nanchang 330100, China;
shaomeijuan@jxutcm.edu.cn (M.S.); 20144031@jxutcm.edu.cn (E.Y.)
* Correspondence: 20060877@jxutcm.edu.cn (Z.Y.); xiexiaoyu@jxutcm.edu.cn (X.X.)

The support information directory

Figure S1: Comparison of IDA and SWATH acquisition methods in HPLC-HRMS.

Table S1: Data-Dependent Acquisition parameters.

Figure S2: Molecular network of HMD. (A) Positive ion mode. (B) Negative ion mode.

Table S2: Detailed information of 472 compounds identified by searching databases and "seed"-based molecular networking.

Table S3: Detailed information on 94 active ingredients screened by TCMSP and Swiss ADME.

Table S4: The Molecular Docking Grid Boxes Parameters

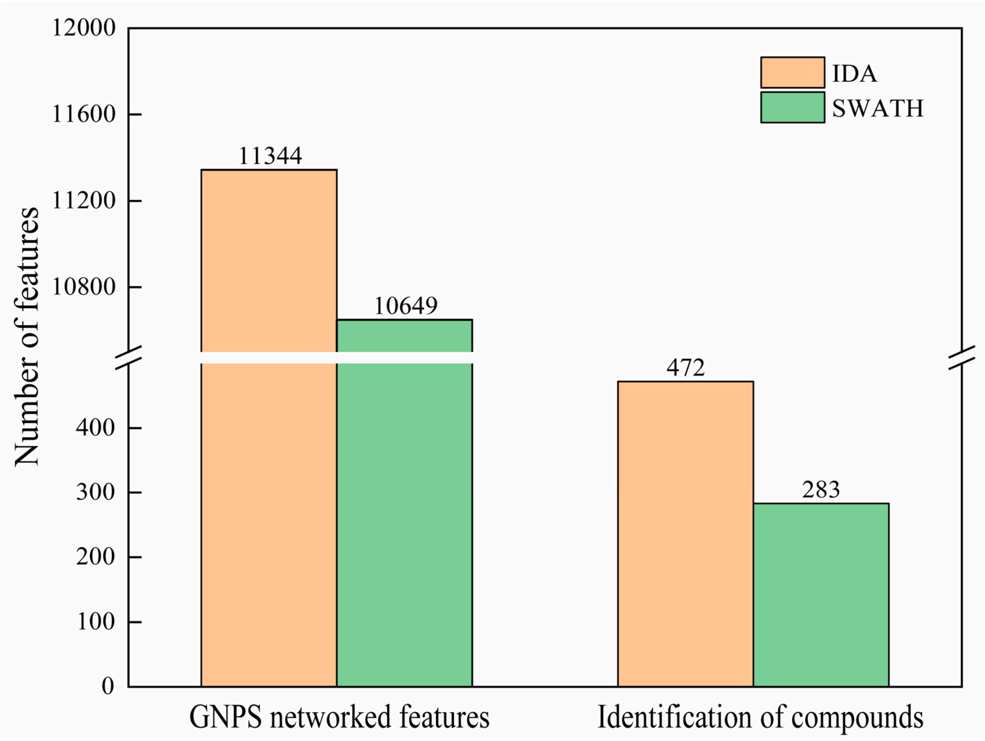

Figure S1. Comparison of IDA and SWATH acquisition methods in HPLC-HRMS.

Table S1 Data-Dependent Acquisition parameters.

Parameters	IDA(+)	IDA(-)	SWATH(+)	SWATH(-)
TOF-MS start mass	200	200	200	200
TOF-MS stop mass	1200	1200	1200	1200
TOF-MS accumulation time (sec)	0.15	0.15	0.15	0.15
DP (V)	80	-80	80	-80
TOF-MS/MS start mass	50	50	50	50
TOF-MS/MS stop mass	1200	1200	1200	1000
TOF-MS/MS accumulation time (sec)	0.05	0.05	0.05	0.05
CE (V)	35	-35	30	-35
CE spread (V)	15	15	15	15
TOP n	10	10	—	—
Intensity threshold exceeds (cps)	100	100	—	—
Dynamic background subtraction	Activated	Activated	—	—
Exclude former candidate ions	Activated	Activated	—	—
For (sec)	4	4	—	—
After (occurrence)	2	2	—	—
SWATH Minimum window width (Da)	—	—	3	3

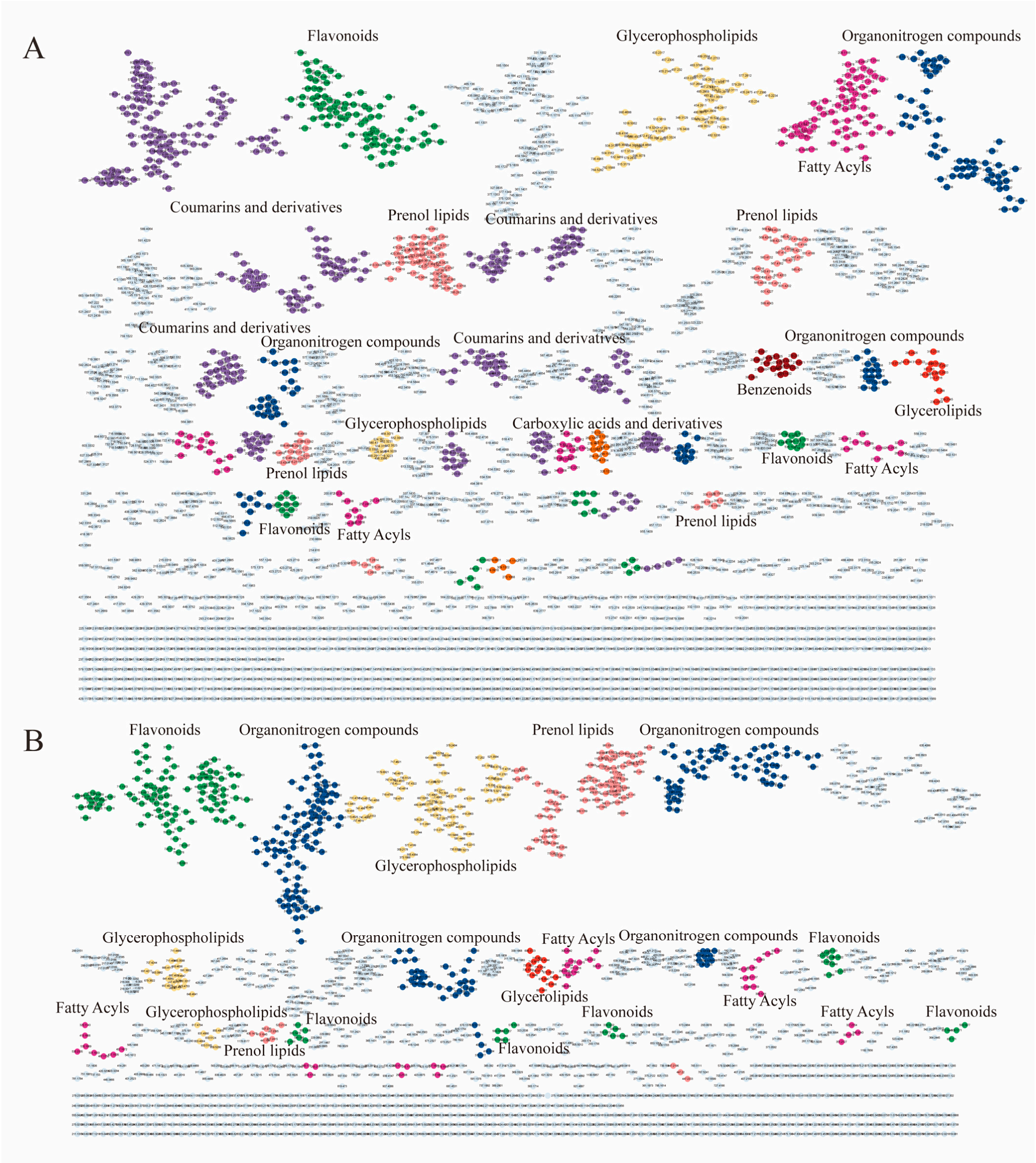

Figure S2. Molecular network of HMD. (A) Positive ion mode. (B) Negative ion. mode.

Table S2 Detailed information of 472 compounds identified by searching databases and "seed"-based molecular networking.

No	Database	t_R/min	m/z	Adduct type	Class	Total score	Name	Formula	MS/MS Fragment Ions	MSI Level	CID*
1	GNPS-MS1	10.21	679.1980	[M+H]+	Flavonoids and derivatives		3,18-Bis(4-Hydroxyphenyl)-10-[(4-Hydroxyphenyl)Methylidene]-4,19-Dioxahexacyclo[15.6.1.12,5.011,16.020,24.09,25]Pentacosa-1(24),5,7,9(25),11(16),12,14,20,22-Nonaene-7,12,14,22-Tetrol	C42H30O9	413.0833,309.0727	Level 3	72788797
2	Authentic standards-MS-DIAL	8.50	269.0419	[M-H]-	Flavonoids and derivatives	88.7	Apigenin	C15H10O5	151.0037,117.0361	Level 2	5280443
3[a]	Authentic standards-MS-DIAL	8.41	271.0582	[M-H]-	Flavonoids and derivatives	92.5	Naringenin	C15H12O5	177.0184,151.0039,119.0498,65.0023	Level 1	932
4	Authentic standards-MS-DIAL	10.47	285.0732	[M-H]-	Flavonoids and derivatives	90.3	Isosakuranetin	C16H14O5	243.0648,146.0095,136.0138,108.0195, 65.0027	Level 2	5118250
5[a]	Authentic standards-MS-DIAL	8.52	287.0541	[M+H]+	Flavonoids and derivatives	96.7	Kaempferol	C15H10O6	213.0543,165.0184,153.0183,137.0226, 121.0291	Level 1	5280863
6	Authentic standards-MS-DIAL	8.72	299.0555	[M-H]-	Flavonoids and derivatives	85.6	Tectorigenin	C16H12O6	284.0314,256.0358,227.0348	Level 2	5281811
7	Authentic standards-MS-DIAL	7.26	303.0502	[M+H]+	Flavonoids and derivatives	97.0	2-(3,4-Dihydroxyphenyl)-3,5,7-Trihydroxy-4H-Chromen-4-One	C15H10O7	257.0438,229.0507,201.0557,165.0186, 153.0205,137.025	Level 2	9839360

8	Authentic standards-MS-DIAL	9.46	361.0924	[M+H]+	Flavonoids and derivatives	90.0	5,7-Dihydroxy-2-(4-Hydroxy-3-Methoxyphenyl)-3,6-Dimethoxychromen-4-One	C18H16O8	345.0568,328.055,303.0479	Level 2	5464461
9	Authentic standards-MS-DIAL	11.74	375.1071	[M+H]+	Flavonoids and derivatives	92.1	Chrysosplenetin B	C19H18O8	345.0583,342.0719,317.0682,299.0533	Level 2	5281608
10[a]	Authentic standards-MS-DIAL	14.07	389.1226	[M+H]+	Flavonoids and derivatives	88.1	Artemitin	C20H20O8	373.0922,356.0884,331.081,313.0701	Level 1	5320351
11	Authentic standards-MS-DIAL	6.24	433.0790	[M-H]-	Flavonoids and derivatives	94.0	Quercetin-3-D-Xyloside	C20H18O11	300.0251,271.0237,243. 0285,178.9985	Level 2	5878729
12[a]	Authentic standards-MS-DIAL	6.41	447.0898	[M-H]-	Flavonoids and derivatives	92.7	Kaempferol-3-O-Glucoside	C21H20O11	284.0326,255.0303,227.0346	Level 1	5462193
13[a]	Authentic standards-MS-DIAL	4.89	449.1072	[M+H]+	Flavonoids and derivatives	94.3	Homoorientin	C21H20O11	413.0876,395.077，353.0668,329.0665,299.0549	Level 1	6426860
14	Authentic standards-MS-DIAL	8.04	463.1223	[M-H]-	Flavonoids and derivatives	79.0	4'-Methoxy-7-O-Beta-D-Glucopyranosyl-8,3'-Dihydroxyflavanone	C22H24O11	301.0695	Level 2	134750977
15	Authentic standards-MS-DIAL	5.44	465.1021	[M+H]+	Flavonoids and derivatives	91.6	Isoquercetin	C21H20O12	303.0548,85.0286	Level 2	5280804
16	Authentic standards-MS-DIAL	5.82	533.1262	[M-H]-	Flavonoids and derivatives	89.7	5,7-Dihydroxy-2-(4-Hydroxyphenyl)-6,8-Bis(3,4,5-Trihydroxyoxan-2-Yl)Chromen-4-One	C25H26O13	443.0966,413.0851,383.078,353.0654	Level 2	14427338

17	Authentic standards-MS-DIAL	5.92	535.1069	[M+H]+	Flavonoids and derivatives	87.8	3-[[6-[2-(3,4-Dihydroxyphenyl)-5-Hydroxy-4-Oxochromen-7-Yl]Oxy-3,4,5-Trihydroxyoxan-2-Yl]Methoxy]-3-Oxopropanoic Acid	C24H22O14	287.0569	Level 2	5858132
18	Authentic standards-MS-DIAL	5.11	563.1357	[M-H]-	Flavonoids and derivatives	91.9	8-[3,4-Dihydroxy-5-(Hydroxymethyl)Oxolan-2-Yl]-5,7-Dihydroxy-2-(4-Hydroxyphenyl)-6-[3,4,5-Trihydroxy-6-(Hydroxymethyl)Oxan-2-Yl]Chromen-4-One	C26H28O14	473.1071,443.0989,383.0763,353.0674	Level 2	10053626
19	Authentic standards-MS-DIAL	4.58	581.1498	[M+H]+	Flavonoids and derivatives	79.2	2-(3,4-Dihydroxyphenyl)-5,7-Dihydroxy-6-[3,4,5-Trihydroxy-6-(Hydroxymethyl)Oxan-2-Yl]-8-(3,4,5-Trihydroxyoxan-2-Yl)Chromen-4-One	C26H28O15	527.1216,425.0873,341.0673	Level 2	3542481
20	Authentic standards-MS-DIAL	8.72	593.1259	[M-H]-	Flavonoids and derivatives	89.8	[2-[5,7-Dihydroxy-2-(4-Hydroxyphenyl)-4-Oxochromen-3-Yl]Oxy-4,5-Dihydroxy-6-(Hydroxymethyl)Oxan-3-Yl] 3-(4-Hydroxyphenyl)Prop-2-Enoate	C30H26O13	307.0826,285.0397	Level 2	71341515
21	Authentic standards-MS-DIAL	6.66	593.1477	[M-H]-	Flavonoids and derivatives	92.1	Kaempferol-7-O-Neohesperidoside	C27H30O15	285.0382	Level 2	22178437
22	Authentic standards-MS-DIAL	5.71	595.1254	[M-H]-	Flavonoids and derivatives	89.0	Quercetin-3-O-Vicianoside	C26H28O16	300.0268,271.0254,178.9971	Level 2	13887800
23	Authentic standards-MS-DIAL	5.06	595.1651	[M+H]+	Flavonoids and derivatives	92.1	3-[3,5-Dihydroxy-6-(Hydroxymethyl)-4-(3,4,5-Trihydroxy-6-Methyloxan-2-Yl)Oxyoxan-2-Yl]Oxy-5,7-Dihydroxy-2-(4-Hydroxyphenyl)Chromen-4-One	C27H30O15	449.109,287.0535,85.0278	Level 2	74124649

24	Authentic standards-MS-DIAL	5.86	609.1433	[M-H]-	Flavonoids and derivatives	94.2	Quercetin-3-O-Rutinoside	C27H30O16	301.0392,271.0286,178.9985	Level 2	5293655
25[a]	Authentic standards-MS-DIAL	5.86	609.1805	[M+H]+	Flavonoids and derivatives	91.2	Diosmetin-7-O-Rutinoside	C28H32O15	463.1254,301.0741	Level 1	5281613
26	Authentic standards-MS-DIAL	4.80	611.1615	[M+H]+	Flavonoids and derivatives	90.5	Rutin	C27H30O16	465.1063,303.0514,129.0541	Level 2	54579866
27	Authentic standards-MS-DIAL	5.93	611.1968	[M+H]+	Flavonoids and derivatives	90.4	Hesperidin	C28H34O15	449.1438,303.0852,137.0592,85.0277	Level 2	10621
28	Authentic standards-MS-DIAL	4.07	627.1537	[M+H]+	Flavonoids and derivatives	89.7	Quercetin-3,4'-O-Di-Beta-Glucoside	C27H30O17	465.1046,303.0499,85.0278	Level 2	5320835
29	Authentic standards-MS-DIAL	5.09	639.1500	[M-H]-	Flavonoids and derivatives	86.8	Isorhamnetin-3-Glucoside-4'-Glucoside	C28H32O17	477.1018,315.0501	Level 2	5901757
30	Authentic standards-MS-DIAL	5.78	639.1901	[M+H]+	Flavonoids and derivatives	89.0	Demethoxycentaureidin 7-O-Rutinoside	C29H34O16	493.1348,331.0797	Level 2	20106119
31	Authentic standards-MS-DIAL	10.65	723.1671	[M-H]-	Flavonoids and derivatives	81.0	[(2S,3S,4S,5R,6S)-6-[5,7-Dihydroxy-2-(4-Hydroxyphenyl)-4-Oxochromen-3-Yl]Oxy-5-Hydroxy-4-[(E)-3-(4-Hydroxyphenyl)Prop-2-Enoyl]Oxy-2-Methyloxan-3-Yl] (E)-3-(4-Hydroxyphenyl)Prop-2-Enoate	C39H32O14	437.1221,285.0425,145.0287	Level 2	51136469

32	Authentic standards-MS-DIAL	5.06	741.2246	[M+H]+	Flavonoids and derivatives	94.4	3-[4,5-Dihydroxy-3-(3,4,5-Trihydroxy-6-Methyloxan-2-Yl)Oxy-6-[(3,4,5-Trihydroxy-6-Methyloxan-2-Yl)Oxymethyl]Oxan-2-Yl]Oxy-5,7-Dihydroxy-2-(4-Hydroxyphenyl)Chromen-4-One	C33H40O19	595.1684,449.1071,287.0603,85.0287	Level 2	5459192
33	Authentic standards-MS-DIAL	4.80	757.2182	[M+H]+	Flavonoids and derivatives	94.6	3-[6-[[(2R,3R,4R,5S,6S)-3,5-Dihydroxy-6-Methyl-4-[(2S,3R,4R,5R,6S)-3,4,5-Trihydroxy-6-Methyloxan-2-Yl]Oxyoxan-2-Yl]Oxymethyl]-3,4,5-Trihydroxyoxan-2-Yl]Oxy-2-(3,4-Dihydroxyphenyl)-5,7-Dihydroxychromen-4-One	C33H40O20	611.1695,465.1045,303.0601,85.0278	Level 2	74095088
34	Authentic standards-MS-DIAL	5.10	771.2355	[M+H]+	Flavonoids and derivatives	92.5	Xanthorhamnin	C34H42O20	625.1754,479.1203,317.0687,85.0278	Level 2	56995003
35	Authentic standards-MS-DIAL	4.59	773.2132	[M+H]+	Flavonoids and derivatives	86.3	2-(3,4-Dihydroxyphenyl)-5-Hydroxy-7-[3,4,5-Trihydroxy-6-(Hydroxymethyl)Oxan-2-Yl]Oxy-3-[3,4,5-Trihydroxy-6-[(3,4,5-Trihydroxy-6-Methyloxan-2-Yl)Oxymethyl]Oxan-2-Yl]Oxychromen-4-One	C33H40O21	611.1647,465.1024,303.0482,147.0664	Level 2	13942388
36	MassBank	12.00	301.0725	[M+H]+	Flavonoids and derivatives	76.6	Kaempferide	C16H12O6	286.0461,153.0177	Level 2	5281666
37[a]	MassBank	12.42	315.0858	[M+H]+	Flavonoids and derivatives	85.5	Velutin	C17H14O6	300.0624,272.0672	Level 1	5464381
38	MassBank	5.89	317.0657	[M+H]+	Flavonoids and derivatives	94.4	Isorhamnetin	C16H12O7	302.0422,285.0396,229.049,153.0179, 93.032	Level 2	5281654

39	MassBank	4.83	319.0425	[M+H]+	Flavonoids and derivatives	80.9	Myricetin	C15H10O8	301.0348,273.0516,153.0228,137.0215	Level 2	5281672
40	MassBank	6.92	431.0970	[M-H]-	Flavonoids and derivatives	96.0	Kaempferol-3-O-Alpha-L-Rhamnoside	C21H20O10	285.0431,255.0302,277.0348	Level 2	5316673
41	MassBank	6.04	463.0868	[M-2H]-	Flavonoids and derivatives	96.6	Delphinidin-3-O-Beta-Glucopyranoside	C21H21O12	300.0282,271.0283,255.03,227.0347	Level 2	443650
42	MassBank	6.02	479.1175	[M]+	Flavonoids and derivatives	92.9	Petunidin-3-O-Beta-Glucopyranoside	C22H23O12	317.0631,302.0421,274.0466,217.0536	Level 2	443651
43	MassBank	8.09	491.1135	[M-2H]-	Flavonoids and derivatives	80.0	Oenin	C23H25O12	329.0636,313.0331,299.0138	Level 2	443652
44	MassBank	5.62	505.0972	[M-H]-	Flavonoids and derivatives	95.1	Quercetin 3-(6-O-Acetyl-Beta-Glucoside)	C23H22O13	300.0267,271.0237,255.0287,178.9984	Level 2	10006384
45[a]	MassBank	6.21	593.1475	[M-H]-	Flavonoids and derivatives	96.7	Kaempferol-3-O-Rutinoside	C27H30O15	285.0493,255.03	Level 1	5318767
46[a]	MassBank	6.31	623.1588	[M-H]-	Flavonoids and derivatives	96.0	Isorhamnetin-3-O-Rutinoside	C28H32O16	315.0486,300.0304,271.0258	Level 1	5481663
47	MassBank	5.94	639.1520	[M-H]-	Flavonoids and derivatives	71.0	Isorhamnetin-3-Glucoside-4'-Glucoside	C28H32O17	315.0495,300.0283	Level 2	11802256
48	MassBank	5.26	757.2152	[M+H]+	Flavonoids and derivatives	85.6	Quercetin-3-O-Alpha-L-Rhamnopyranosyl(1-2)-Beta-D-Glucopyranoside-7-O-Alpha-L-Rhamnopyranoside	C33H40O20	611.1571,449.1111,303.0498,85.0269	Level 2	10327769
49	MassBank	5.45	951.1833	[2M+Na]+	Flavonoids and derivatives	80.7	Hyperoside	C21H20O12	487.0858,325.032	Level 2	5281643
50	ReSpect	6.39	435.0922	[M+H]+	Flavonoids and derivatives	84.9	Quercetin-3-Arabinoside	C20H18O11	303.0496,229.094,153.0155	Level 2	5481224
51	GNPS	5.26	303.0508	[M+H]+	Flavonoids and derivatives	91.5	5,7-Dihydroxy-2-(2,3,4-Trihydroxyphenyl)Chromen-4-One	C15H10O7	285.0396,257.0437,229.0491,153.0192,137.0238	Level 2	18471844

52	GNPS	6.84	477.1001	[M-H]-	Flavonoids and derivatives	77.9	5,7-Dihydroxy-2-[4-Hydroxy-3-[(2S,3R,4S,5S,6R)-3,4,5-Trihydroxy-6-(Hydroxymethyl)Oxan-2-Yl]Oxyphenyl]-3-Methoxychromen-4-One	C22H22O12	315.0498,299.0178,255.0287	Level 2	74978418
53	GNPS	4.02	581.1501	[M+H]+	Flavonoids and derivatives	76.8	5,7-Dihydroxy-2-(4-Hydroxyphenyl)-3-[(2S,3R,4S,5S,6R)-3,4,5-Trihydroxy-6-[[(2S,3R,4S,5S)-3,4,5-Trihydroxyoxan-2-Yl]Oxymethyl]Oxan-2-Yl]Oxychromen-4-One	C26H28O15	287.0555,85.0243	Level 2	14334890
54	GNPS	4.88	593.1458	[M-H]-	Flavonoids and derivatives	83.0	5,7-Dihydroxy-3-(4-Hydroxyphenyl)-6,8-Bis[3,4,5-Trihydroxy-6-(Hydroxymethyl)Oxan-2-Yl]Chromen-4-One	C27H30O15	503.1183,473.1005,383.0748,353.0659	Level 2	4484951
55	GNPS	5.32	625.1384	[M-H]-	Flavonoids and derivatives	89.7	3-[3,4-Dihydroxy-6-(Hydroxymethyl)-5-[3,4,5-Trihydroxy-6-(Hydroxymethyl)Oxan-2-Yl]Oxyoxan-2-Yl]Oxy-2-(3,4-Dihydroxyphenyl)-5,7-Dihydroxychromen-4-One	C27H30O17	300.0266,271.0252,178.997	Level 2	14048507
56	GNPS	4.99	755.1990	[M-H]-	Flavonoids and derivatives	84.5	5,7-Dihydroxy-2-[4-[(2S,3R,4S,5S,6R)-3,4,5-Trihydroxy-6-(Hydroxymethyl)Oxan-2-Yl]Oxyphenyl]-3-[(2S,3R,4S,5S,6R)-3,4,5-Trihydroxy-6-[[(2R,3R,4R,5R,6S)-3,4,5-Trihydroxy-6-Methyloxan-2-Yl]Oxymethyl]Oxan-2-Yl]Oxychromen-4-One	C33H40O20	593.1519,285.0401	Level 2	74978021
57	GNPS	5.10	773.2127	[M+H]+	Flavonoids and derivatives	75.6	Quercetin-3-O-Glc-1-3-Rham-1-6-Glucoside	C33H40O21	465.1021,303.0514	Level 2	44715854

58	GNPS	4.09	773.2132	[M+H]+	Flavonoids and derivatives	80.3	3-[(2S,3R,4S,5R,6S)-3,4-Dihydroxy-6-Methyl-5-[(2S,3R,4S,5S,6R)-3,4,5-Trihydroxy-6-(Hydroxymethyl)Oxan-2-Yl]Oxyoxan-2-Yl]Oxy-2-(3,4-Dihydroxyphenyl)-5-Hydroxy-7-[3,4,5-Trihydroxy-6-(Hydroxymethyl)Oxan-2-Yl]Oxychromen-4-One	C33H40O21	465.1109,303.0511,85.0278	Level 2	75111068
59	Fiehn HILIC	5.60	449.1070	[M+H]+	Flavonoids and derivatives	80.1	Quercetin-3-Ramnoside	C21H20O11	287.0552,97.0282,85.0277	Level 2	5353915
60	Fiehn HILIC	6.51	477.1021	[M-H]-	Flavonoids and derivatives	98.5	Isorhamnetin 3-Galactoside	C22H22O12	314.0451,271.0252,243.0315,151.0038	Level 2	13245586
61	RIKEN PlaSMA	6.84	447.0898	[M-H]-	Flavonoids and derivatives	73.3	Luteolin-7-O-Glucoside	C21H20O11	285.0382,151.0014	Level 2	5291488
62	RIKEN PlaSMA	5.52	447.0900	[M-H]-	Flavonoids and derivatives	83.6	Luteolin-8-C-Glucoside	C21H20O11	357.0596,327.0482,297.0387,133.0274	Level 2	5382105
63	RIKEN PlaSMA	5.60	595.1650	[M+H]+	Flavonoids and derivatives	90.4	Datiscetin-3-O-Rutinoside	C27H30O15	449.1172,287.0684,85.0286	Level 2	5883291
64	RIKEN PlaSMA	6.68	609.1771	[M-H]-	Flavonoids and derivatives	88.6	Hesperetin-7-O-Rutinoside	C28H34O15	325.0717,301.0716,242.058	Level 2	3594
65	RIKEN PlaSMA bio-MS/MS from plant tissues	4.07	465.1003	[M+H]+	Flavonoids and derivatives	86.3	Quercetin-4'-O-Glucoside	C21H20O12	303.0516,257.0406,201.0599,165.0148	Level 2	12442954
66	RIKEN PlaSMA bio-MS/MS from plant tissues	5.06	579.1303	[M-H]-	Flavonoids and derivatives	72.8	2-(3,4-Dihydroxyphenyl)-5,7-Dihydroxy-8-[3,4,5-Trihydroxy-6-(Hydroxymethyl)Oxan-2-Yl]-6-(3,4,5-Trihydroxyoxan-2-Yl)Chromen-4-One	C26H28O15	489.1023,399.0724,369.0592	Level 2	73802481

67	RIKEN PlaSMA bio-MS/MS from plant tissues	4.72	581.1480	[M-H]-	Flavonoids and derivatives	87.1	2-(3,4-Dihydroxyphenyl)-7-Hydroxy-5-[3,4,5-Trihydroxy-6-(Hydroxymethyl)Oxan-2-Yl]Oxy-8-(3,4,5-Trihydroxyoxan-2-Yl)-2,3-Dihydrochromen-4-One	C26H30O15	461.1082,371.076,239.0535,209.0449	Level 2	139292024
68	RIKEN PlaSMA bio-MS/MS from plant tissues	7.69	593.1237	[M-H]-	Flavonoids and derivatives	89.9	[6-[5,7-Dihydroxy-2-(4-Hydroxyphenyl)-4-Oxochromen-3-Yl]Oxy-3,4,5-Trihydroxyoxan-2-Yl]Methyl 3-(4-Hydroxyphenyl)Prop-2-Enoate	C30H26O13	447.1012,307.0809,225.0225	Level 2	57369598
69	RIKEN PlaSMA bio-MS/MS from plant tissues	5.08	623.1589	[M-H]-	Flavonoids and derivatives	94.8	5,7-Dihydroxy-2-(4-Hydroxy-3-Methoxyphenyl)-3-[3,4,5-Trihydroxy-6-[(3,4,5-Trihydroxy-6-Methyloxan-2-Yl)Oxymethyl]Oxan-2-Yl]Oxychromen-4-One	C28H32O16	315.0518,299.0181	Level 2	6223069
70	RIKEN PlaSMA bio-MS/MS from plant tissues	5.36	741.1838	[M-H]-	Flavonoids and derivatives	86.9	2-(3,4-Dihydroxyphenyl)-3-[4,5-Dihydroxy-6-[(3,4,5-Trihydroxy-6-Methyloxan-2-Yl)Oxymethyl]-3-(3,4,5-Trihydroxyoxan-2-Yl)Oxyoxan-2-Yl]Oxy-5,7-Dihydroxychromen-4-One	C32H38O20	591.1393,300.027, 178.9973	Level 2	5859848
71	RIKEN PlaSMA bio-MS/MS from plant tissues	4.71	771.1943	[M-H]-	Flavonoids and derivatives	87.4	3-[4,5-Dihydroxy-6-(Hydroxymethyl)-3-[3,4,5-Trihydroxy-6-(Hydroxymethyl)Oxan-2-Yl]Oxyoxan-2-Yl]Oxy-2-(3,4-Dihydroxyphenyl)-5-Hydroxy-7-(3,4,5-Trihydroxy-6-Methyloxan-2-Yl)Oxychromen-4-One	C33H40O21	609.1477,462.079,301.0345	Level 2	74978168
72	Fiehn/Vaniya natural	8.68	329.0646	[M-H]-	Flavonoids and derivatives	85.8	(2Z)-4,6-Dihydroxy-2-[(4-Hydroxy-3,5-Dimethoxyphenyl)Methylidene]-1-Benzofuran-3-One	C17H14O7	314.0416,299.0176,271.0236	Level 2	490065

	product library										
73	Fiehn/Vaniya natural product library	10.41	343.0790	[M-H]-	Flavonoids and derivatives	86.5	5,7-Dihydroxy-3,6-Dimethoxy-2-(4-Methoxyphenyl)Chromen-4-One	C18H16O7	328.057,313.0336,198.012,270.0147	Level 2	5281695
74	Fiehn/Vaniya natural product library	4.02	449.1076	[M+H]+	Flavonoids and derivatives	92.2	Luteolin 4'-O-Glucoside	C21H20O11	287.0555	Level 2	12304737
75	Fiehn/Vaniya natural product library	5.68	463.1259	[M+H]+	Flavonoids and derivatives	85.1	5,8-Dihydroxy-2-(4-Hydroxyphenyl)-7-Methoxy-3-[(2S,3R,4R,5R,6S)-3,4,5-Trihydroxy-6-Methyloxan-2-Yl]Oxychromen-4-One	C22H22O11	317.0651,85.0287, 71.0484	Level 2	85503083
76	Fiehn/Vaniya natural product library	5.61	549.0864	[M-H]-	Flavonoids and derivatives	93.2	Quercetin 3-O-Malonylglucoside	C24H22O15	505.0962,463.0863,300.027,271.024	Level 2	14730813
77	Fiehn/Vaniya natural product library	4.85	565.1550	[M+H]+	Flavonoids and derivatives	90.6	5,7-Dihydroxy-2-(4-Hydroxyphenyl)-8-[3,4,5-Trihydroxy-6-(Hydroxymethyl)Oxan-2-Yl]-6-(3,4,5-Trihydroxyoxan-2-Yl)Chromen-4-One	C26H28O14	547.1494,511.1226,427.106,409.0936, 379.0791,325.0692	Level 2	13644660
78	Fiehn/Vaniya natural product library	4.51	595.1650	[M+H]+	Flavonoids and derivatives	87.2	Apigenin 6,8-Digalactoside	C27H30O15	577.1547,559.1445,475.1234,457.1116, 379.0811	Level 2	3084407

79	Fiehn/Vaniya natural product library	6.62	607.1641	[M-H]-	Flavonoids and derivatives	89.2	7-[(2S,3R,4S,5S,6R)-4,5-Dihydroxy-6-(Hydroxymethyl)-3-[(2S,3R,4R,5R,6S)-3,4,5-Trihydroxy-6-Methyloxan-2-Yl]Oxyoxan-2-Yl]Oxy-5-Hydroxy-2-(4-Hydroxy-3-Methoxyphenyl)Chromen-4-One	C28H32O15	299.0565,284.0313	Level 2	74977715
80	Fiehn/Vaniya natural product library	5.35	641.1713	[M+H]+	Flavonoids and derivatives	88.4	3-[(2S,3R,4S,5S,6R)-4,5-Dihydroxy-6-(Hydroxymethyl)-3-[(2S,3R,4S,5S,6R)-3,4,5-Trihydroxy-6-(Hydroxymethyl)Oxan-2-Yl]Oxyoxan-2-Yl]Oxy-2-(3,4-Dihydroxyphenyl)-5-Hydroxy-7-Methoxychromen-4-One	C28H32O17	479.1158,317.0651	Level 2	71341751
81	Fiehn/Vaniya natural product library	5.65	739.2059	[M-H]-	Flavonoids and derivatives	91.7	7-[6-[[3,4-Dihydroxy-6-Methyl-5-[3,4,5-Trihydroxy-6-(Hydroxymethyl)Oxan-2-Yl]Oxyoxan-2-Yl]Oxymethyl]-3,4,5-Trihydroxyoxan-2-Yl]Oxy-5-Hydroxy-3-(4-Hydroxyphenyl)Chromen-4-One	C33H40O19	284.0351	Level 2	23955869
82	Fiehn/Vaniya natural product library	4.35	757.2178	[M+H]+	Flavonoids and derivatives	96.0	5,7-Dihydroxy-2-[4-[(2S,3R,4S,5S,6R)-3,4,5-Trihydroxy-6-(Hydroxymethyl)Oxan-2-Yl]Oxyphenyl]-3-[(2S,3R,4S,5S,6R)-3,4,5-Trihydroxy-6-[[(2R,3R,4R,5R,6S)-3,4,5-Trihydroxy-6-Methyloxan-2-Yl]Oxymethyl]Oxan-2-Yl]Oxychromen-4-One	C33H40O20	611.157,449.1069, 287.0568	Level 2	45359993

83	Fiehn/Vaniya natural product library	3.78	919.2694	[M+H]+	Flavonoids and derivatives	83.0	3-[(2S,3R,4S,5R,6R)-3-[(2S,3R,4R,5S,6S)-3,5-Dihydroxy-6-Methyl-4-[(2S,3R,4S,5S,6R)-3,4,5-Trihydroxy-6-(Hydroxymethyl)Oxan-2-Yl]Oxyoxan-2-Yl]Oxy-4,5-Dihydroxy-6-[[(2R,3R,4R,5R,6S)-3,4,5-Trihydroxy-6-Methyloxan-2-Yl]Oxymethyl]Oxan-2-Yl]Oxy-2-(3,4-Dihydroxyphenyl)-5,7-Dihydroxychromen-4-One	C39H50O25	773.213,611.163,465.103,303.0486	Level 2	85279218
84	GNPS-MS1	4.28	398.1096	[M+H]+	Flavonoids and derivatives		9-(1,1-Dioxothiolan-3-Yl)-3-Phenyl-8,10-Dihydropyrano[2,3-F][1,3]Benzoxazin-4-One	C21H19NO5S	219.0292,97.0273	Level 3	17601724
85	GNPS-MS1	6.75	440.1667	[M+H]+	Flavonoids and derivatives		2-[[2-[4-(4-Methoxyphenyl)-8-Methyl-2-Oxochromen-7-Yl]Oxyacetyl]Amino]Pentanoic Acid	C24H25NO7	309.0728,203.0337	Level 3	4357287
86	GNPS-MS1	4.67	461.0689	[M-H]-	Flavonoids and derivatives		1,8,9-Trihydroxy-3-[3,4,5-Trihydroxy-6-(Hydroxymethyl)Oxan-2-Yl]Oxy-[1]Benzofuro[3,2-C]Chromen-6-One	C21H18O12	298.0363,158.0880	Level 3	74029678
87	GNPS-MS1	0.80	467.0786	[M+H]+	Flavonoids and derivatives		Methyl 4-[[6-(4-Methoxyphenyl)Sulfonyloxy-3-Oxo-1-Benzofuran-2-Ylidene]Methyl]Benzoate	C24H18O8S	265.1590,135.079	Level 3	72185813
88	GNPS-MS1	5.34	468.1123	[M+H]+	Flavonoids and derivatives		5-Hydroxy-3-(4-Hydroxyphenyl)-10-(2-Oxo-1H-Quinolin-3-Yl)-9,10-Dihydropyrano[2,3-H]Chromene-4,8-Dione	C27H17NO7	203.0338,69.0333	Level 3	71827365
89	GNPS-MS1	4.93	484.1056	[M+H]+	Flavonoids and derivatives		2-(3,4-Dihydroxyphenyl)-3,5-Dihydroxy-10-Quinolin-6-Yl-9,10-	C27H17NO8	219.0291,173.0234,127.0391	Level 3	75491537

						Dihydropyrano[2,3-H]Chromene-4,8-Dione				
90	GNPS-MS1	4.54	487.1783	[M-H]-	Flavonoids and derivatives	Methyl 3-[7-Hydroxy-3-(4-Hydroxyphenyl)-4-Oxochromen-8-Yl]-3-[4-(2-Methylpropoxy)Phenyl]Propanoate	C29H28O7	361.0552,217.0135,59.0137	Level 3	71826701
91	GNPS-MS1	3.48	521.2136	[M+H]+	Flavonoids and derivatives	5,7,20-Trihydroxy-6-(3-Hydroxy-3-Methylbutyl)-17,17-Dimethyl-8-(3-Methylbut-2-Enyl)-10,12,18-Trioxapentacyclo[11.8.0.02,11.04,9.014,19]Henicosa-1(21),2(11),4,6,8,13,15,19-Octaen-3-One	C30H32O8	205.0975,188.0708,146.0603	Level 3	636550
92	GNPS-MS1	4.23	544.1642	[M+H]+	Flavonoids and derivatives	Methyl 3-(7-Methoxy-1-Methyl-2-Oxoquinolin-3-Yl)-3-(5,6,7-Trihydroxy-4-Oxo-2-Phenylchromen-8-Yl)Propanoate	C30H25NO9	365.0869,203.0354	Level 3	75491244
93	GNPS-MS1	6.39	544.2021	[M+H]+	Flavonoids and derivatives	5,7-Dihydroxy-8-[1-(7-Methoxy-1,3-Benzodioxol-5-Yl)-3-Oxo-3-Piperidin-1-Ylpropyl]-2-Phenylchromen-4-One	C31H29NO8	261.1118,189.0543	Level 3	110207919
94	GNPS-MS1	3.20	565.1180	[M+H]+	Flavonoids and derivatives	3-[[6-[2-(3,4-Dihydroxyphenyl)-5-Hydroxy-3-Methoxy-4-Oxochromen-7-Yl]Oxy-3,4,5-Trihydroxyoxan-2-Yl]Methoxy]-3-Oxopropanoic Acid	C25H24O15	403.0648,241.0097	Level 3	163005268
95	GNPS-MS1	17.30	566.2727	[M+H]+	Flavonoids and derivatives	9-[1-(Ethylamino)-4-Hydroxybutoxy]-3-(4-Hydroxy-3,5-Dimethoxyphenyl)-7-Methoxy-3,4,5,6-Tetrahydro-2H-Naphtho[2,1-F]Chromen-4-Ol	C32H39NO8	261.1115,243.1009,189.0541	Level 3	163031831

96	GNPS-MS1	10.58	569.1780	[M+H]+	Flavonoids and derivatives	5-Hydroxy-3-(4-Hydroxyphenyl)-7-Methoxy-2-[7-Methoxy-3-(4-Methoxyphenyl)-4-Oxo-2,3-Dihydrochromen-2-Yl]-2,3-Dihydrochromen-4-One	C33H28O9	309.0742,283.0951	Level 3	162863278
97	GNPS-MS1	9.01	577.1317	[M-H]-	Flavonoids and derivatives	[4,5-Dihydroxy-2-[5-Hydroxy-3-(4-Hydroxyphenyl)-4-Oxochromen-7-Yl]Oxy-6-(Hydroxymethyl)Oxan-3-Yl] 3-(4-Hydroxyphenyl)Prop-2-Enoate	C30H26O12	285.0396,63.5775	Level 3	162911492
98	GNPS-MS1	4.78	579.1310	[M+H]+	Flavonoids and derivatives	3-Oxo-3-[[3,4,5-Trihydroxy-6-[[5-Hydroxy-3-(6-Methoxy-1,3-Benzodioxol-5-Yl)-4-Oxo-2,3-Dihydrochromen-7-Yl]Oxy]Oxan-2-Yl]Methoxy]Propanoic Acid	C26H26O15	341.0973,203.0464	Level 3	74413691
99	GNPS-MS1	5.22	579.1663	[M+H]+	Flavonoids and derivatives	10-[2-[2-(2,3-Dihydro-1-Benzofuran-5-Yl)Ethoxy]Phenyl]-2-(3,4-Dihydroxyphenyl)-5-Hydroxy-9,10-Dihydropyrano[2,3-H]Chromene-4,8-Dione	C34H26O9	347.0951,155.1077	Level 3	71826369
100	GNPS-MS1	28.85	589.3115	[M+H]+	Flavonoids and derivatives	2-(3-Cyclohexyloxy-4-Hydroxy-5-Methoxyphenyl)-11-Methoxy-6-(3-Methylbutyl)-3,4,5,6-Tetrahydro-2H-Naphtho[2,1-F]Chromene-3,8-Diol	C36H44O7	387.2849,225.0140	Level 3	163098226
101	GNPS-MS1	21.57	595.1328	[M+H]+	Flavonoids and derivatives	[3-Acetyloxy-2-[5,7-Dihydroxy-2-(4-Hydroxy-3,5-Dimethoxyphenyl)-4-Oxochromen-3-Yl]Oxy-6-Hydroperoxy-5-Hydroxyoxan-4-Yl] Acetate	C26H26O16	526.0655,239.9799	Level 3	162961064

102	GNPS-MS1	9.60	595.1563	[M+H]+	Flavonoids and derivatives	7-[3-(2-Hydroxy-4-Methoxyphenyl)-6-Methoxy-4-Oxochromen-7-Yl]Oxy-6-Methoxy-3-(4-Methoxyphenyl)Chromen-4-One	C34H26O10	309.0759,93.0678	Level 3	11169313
103	GNPS-MS1	9.29	607.1420	[M-H]-	Flavonoids and derivatives	[3,4,5-Trihydroxy-6-[5-Hydroxy-2-(4-Hydroxy-3-Methoxyphenyl)-4-Oxochromen-7-Yl]Oxyoxan-2-Yl]Methyl 3-(4-Hydroxyphenyl)Prop-2-Enoate	C31H28O13	285.0398,126.4836	Level 3	163049991
104	GNPS-MS1	12.80	613.1682	[M+H]+	Flavonoids and derivatives	2-(3,4-Dihydroxyphenyl)-3,5-Dihydroxy-10-[3-Methoxy-2-[2-(4-Methoxyphenyl)Ethoxy]Phenyl]-9,10-Dihydropyrano[2,3-H]Chromene-4,8-Dione	C34H28O11	327.0844,167.1084	Level 3	75492576
105	GNPS-MS1	5.37	651.1883	[M+H]+	Flavonoids and derivatives	[2-[5,7-Dihydroxy-2-(4-Methoxyphenyl)-4-Oxo-6-[3,4,5-Trihydroxy-6-(Hydroxymethyl)Oxan-2-Yl]Chromen-8-Yl]-4,5-Dihydroxy-6-(Hydroxymethyl)Oxan-3-Yl] Acetate	C30H34O16	365.1052,108.0418	Level 3	163040001
106	GNPS-MS1	3.09	705.1630	[M-H]-	Flavonoids and derivatives	[5,13-Bis(3,4-Dihydroxyphenyl)-9,17,19,21-Tetrahydroxy-4,12,14-Trioxapentacyclo[11.7.1.02,11.03,8.015,20]Henicosa-2(11),3(8),9,15,17,19-Hexaen-6-Yl] 3-Phenylprop-2-Enoate	C39H30O13	353.0923,191.0628	Level 3	73810146
107	GNPS-MS1	4.70	727.1740	[M+H]+	Flavonoids and derivatives	4-[[6-[2-(3,4-Dihydroxyphenyl)-5-Hydroxy-4-Oxo-7-[3,4,5-Trihydroxy-6-(Hydroxymethyl)Oxan-2-Yl]Oxychromen-3-Yl]Oxy-3,4,5-Trihydroxyoxan-2-Yl]Methoxy]-3-Oxobutanoic Acid	C31H34O20	479.1179,317.0668,85.0287	Level 3	162914691

108	GNPS-MS1	10.26	739.1597	[M-H]-	Flavonoids and derivatives	[6-[5,7-Dihydroxy-2-(4-Hydroxyphenyl)-4-Oxochromen-3-Yl]Oxy-4,5-Dihydroxy-3-[3-(4-Hydroxyphenyl)Prop-2-Enoyloxy]Oxan-2-Yl]Methyl 3-(4-Hydroxyphenyl)Prop-2-Enoate	C39H32O15	453.1168,285.0393,161.0239	Level 3	163089772
109	GNPS-MS1	6.39	741.1842	[M+H]+	Flavonoids and derivatives	6-[6-[2-[3,4-Dihydroxy-5-(2-Hydroxyethyl)Phenyl]-5-Hydroxy-4-Oxochromen-7-Yl]Oxy-3,4-Dihydroxy-5-(3-Hydroxypropoxy)Oxane-2-Carbonyl]Oxy-3,4,5-Trihydroxyoxane-2-Carboxylic Acid	C32H36O20	493.1348,331.0815	Level 3	163056191
110	GNPS-MS1	5.38	753.1798	[M-H]-	Flavonoids and derivatives	[3,5-Dihydroxy-6-[5-Hydroxy-2-(4-Hydroxy-3-Methoxyphenyl)-4-Oxochromen-7-Yl]Oxy-4-[3-(4-Hydroxyphenyl)Prop-2-Enoyloxy]Oxan-2-Yl]Methyl 3-(4-Hydroxyphenyl)Prop-2-Enoate	C40H34O15	300.0355,59.013	Level 3	73802639
111	GNPS-MS1	4.58	859.2138	[M+H]+	Flavonoids and derivatives	3-[[6-[4,5-Dihydroxy-2-[5-Hydroxy-2-(4-Hydroxyphenyl)-4-Oxo-7-[3,4,5-Trihydroxy-6-(Hydroxymethyl)Oxan-2-Yl]Oxychromen-3-Yl]Oxy-6-(Hydroxymethyl)Oxan-3-Yl]Oxy-3,4,5-Trihydroxyoxan-2-Yl]Methoxy]-3-Oxopropanoic Acid	C36H42O24	713.1530,551.1046,303.05	Level 3	163058162

112	GNPS-MS1	4.84	859.2510	[M+H]+	Flavonoids and derivatives	7-[3,4-Dihydroxy-4-[[3,4,5-Trihydroxy-6-(Hydroxymethyl)Oxan-2-Yl]Oxymethyl]Oxolan-2-Yl]Oxy-3-[4-[3,4-Dihydroxy-4-[[3,4,5-Trihydroxy-6-(Hydroxymethyl)Oxan-2-Yl]Oxymethyl]Oxolan-2-Yl]Oxyphenyl]-5-Hydroxychromen-4-One	C37H46O23	551.1387,303.0498,85.0287	Level 3	162900670
113	GNPS-MS1	4.91	873.2289	[M+H]+	Flavonoids and derivatives	3-[[6-[3-[4,5-Dihydroxy-6-(Hydroxymethyl)-3-(3,4,5-Trihydroxy-6-Methyloxan-2-Yl)Oxyoxan-2-Yl]Oxy-5-Hydroxy-2-(4-Hydroxyphenyl)-4-Oxochromen-7-Yl]Oxy-3,4,5-Trihydroxyoxan-2-Yl]Methoxymethoxy]-3-Oxopropanoic Acid	C37H44O24	565.1183,317.0651,85.0287	Level 3	74978130
114	GNPS-MS1	4.16	949.2805	[M+H]+	Flavonoids and derivatives	5,7-Dihydroxy-3-[4-Hydroxy-6-(Hydroxymethyl)-3,5-Bis[(3,4,5-Trihydroxy-6-Methyloxan-2-Yl)Oxy]Oxan-2-Yl]Oxy-2-[4-Hydroxy-3-Methoxy-5-[3,4,5-Trihydroxy-6-(Hydroxymethyl)Oxan-2-Yl]Oxyphenyl]Chromen-4-One	C40H52O26	641.1706,479.1184,317.0655	Level 3	163012566
115	GNPS-MS1	5.82	991.2648	[M-H]-	Flavonoids and derivatives	[6-[4,5-Dihydroxy-2-[5-Hydroxy-2-(4-Hydroxy-3-Methoxyphenyl)-4-Oxo-7-(3,4,5-Trihydroxy-6-Methyloxan-2-Yl)Oxychromen-3-Yl]Oxy-6-(Hydroxymethyl)Oxan-3-Yl]Oxy-3,4,5-Trihydroxyoxan-2-Yl]Methyl 3-(4-	C45H52O25	609.1469,301.034	Level 3	78121662

						Hydroxy-3,5-Dimethoxyphenyl)Prop-2-Enoate				
116	GNPS-MS1	6.30	1035.2903	[M-H]-	Flavonoids and derivatives	[2-[3-[4,5-Dihydroxy-3-[3,4,5-Trihydroxy-6-(Hydroxymethyl)Oxan-2-Yl]Oxy-6-[(3,4,5-Trihydroxy-6-Methyloxan-2-Yl)Oxymethyl]Oxan-2-Yl]Oxy-5-Hydroxy-2-(4-Hydroxyphenyl)-4-Oxochromen-7-Yl]Oxy-4,5-Dihydroxy-6-Methyloxan-3-Yl] 2-Methoxybenzoate	C47H56O26	623.16,315.05	Level 3	85175906
117	GNPS-MS1	6.23	1047.2933	[M-H]-	Flavonoids and derivatives	[6-[2-(3,4-Dihydroxyphenyl)-5-Hydroxy-4-Oxo-7-(3,4,5-Trihydroxy-6-Methyloxan-2-Yl)Oxychromen-3-Yl]Oxy-4-Hydroxy-5-(3,4,5-Trihydroxy-6-Methyloxan-2-Yl)Oxy-2-[(3,4,5-Trihydroxy-6-Methyloxan-2-Yl)Oxymethyl]Oxan-3-Yl] 3-(4-Hydroxyphenyl)Prop-2-Enoate	C48H56O26	593.149,285.0382	Level 3	73817257
118	GNPS-MS1	5.93	1161.2891	[M-H]-	Flavonoids and derivatives	2-[3-[5-[6-[5,7-Dihydroxy-2-[3-[2-Hydroxy-5-(5-Hydroxy-7-Methoxy-6-Methyl-4-Oxochromen-2-Yl)Phenyl]-4-Methoxyphenyl]-4-Oxo-2,3-Dihydrochromen-6-Yl]-5,7-Dihydroxy-4-Oxo-2,3-Dihydrochromen-2-Yl]-2-Methoxyphenyl]-4-Hydroxyphenyl]-5-	C66H50O20	609.1472,260.1984	Level 3	73804420

							Hydroxy-7-Methoxy-6-Methylchromen-4-One				
119	GNPS	5.91	431.0988	[M-H]-	Flavonoids and derivatives	93.7	5,7-Dihydroxy-2-(4-Hydroxyphenyl)-3-(3,4,5-Trihydroxy-6-Methyloxan-2-Yl)Oxychromen-4-One	C21H20O10	227.0699,83.0493	Level 2	5835713
120	GNPS	6.60	449.1066	[M+H]+	Flavonoids and derivatives	93.2	3,5,7-Trihydroxy-2-[4-[3,4,5-Trihydroxy-6-(Hydroxymethyl)Oxan-2-Yl]Oxyphenyl]Chromen-4-One	C21H20O11	160.5478	Level 2	15926902
121	GNPS	6.86	463.0868	[M-H]-	Flavonoids and derivatives	98.5	2-(3,4-Dihydroxyphenyl)-5,7-Dihydroxy-3-[3,4,5-Trihydroxy-6-(Hydroxymethyl)Oxan-2-Yl]Oxychromen-4-One	C21H20O12	300.0267,271.0238,255.0287	Level 2	5378597
122	GNPS	18.26	593.1424	[M+H]+	Flavonoids and derivatives	84.7	5,7-Dihydroxy-2-(4-Hydroxyphenyl)-6-[(2R,3R,4R,6R)-3,4,5-Trihydroxy-6-(Hydroxymethyl)Oxan-2-Yl]-8-[(3R,4R,6R)-3,4,5-Trihydroxy-6-(Hydroxymethyl)Oxan-2-Yl]Chromen-4-One	C27H30O15	503.1083,383.0748,353.0659	Level 2	134729580
123	GNPS	4.69	609.1411	[M-H]-	Flavonoids and derivatives	95.9	2-(3,4-Dihydroxyphenyl)-5,7-Dihydroxy-3-[(2S,3R,4S,5S,6R)-3,4,5-Trihydroxy-6-[[(2R,3R,4R,5R,6S)-3,4,5-Trihydroxy-6-Methyloxan-2-Yl]Oxymethyl]Oxan-2-Yl]Oxychromen-4-One	C27H30O16	300.0388,179.0536	Level 2	5280805

124	GNPS	5.86	625.1319	[M-H]-	Flavonoids and derivatives	89.9	[(2S,3S,4R,5S,6R)-6-[2-(3,4-Dihydroxyphenyl)-5,7-Dihydroxy-4-Oxochromen-3-Yl]Oxy-3,4,5-Trihydroxyoxan-2-Yl]Methyl (E)-3-(3,4-Dihydroxyphenyl)Prop-2-Enoate	C30H26O15	255.0274,178.9973	Level 2	134737993
125	GNPS	4.57	711.1354	[M-H]-	Flavonoids and derivatives	75.4	3-[[6-[2-(3,4-Dihydroxyphenyl)-5-Hydroxy-4-Oxo-7-[3,4,5-Trihydroxy-6-(Hydroxymethyl)Oxan-2-Yl]Oxychromen-3-Yl]Oxy-3,4,5-Trihydroxyoxan-2-Yl]Methoxy]-3-Oxopropanoic Acid	C30H32O20	667.1495,462.0785	Level 2	74978238
126	GNPS	5.15	771.1920	[M-H]-	Flavonoids and derivatives	77.4	3-[4,5-Dihydroxy-6-(Hydroxymethyl)-3-(4,5,6-Trihydroxy-2-Methyloxan-3-Yl)Oxyoxan-2-Yl]Oxy-2-(3,4-Dihydroxyphenyl)-5-Hydroxy-7-[3,4,5-Trihydroxy-6-(Hydroxymethyl)Oxan-2-Yl]Oxychromen-4-One	C33H40O21	609.1477,462.0792	Level 2	133053381
127	GNPS-MS1	5.20	447.1254	[M+H]+	Flavonoids and derivatives		3-(4-Methoxyphenoxy)-7-[3,4,5-Trihydroxy-6-(Hydroxymethyl)Oxan-2-Yl]Oxychromen-4-One	C22H22O10	315.0491,203.0533	Level 3	5091525
128	Authentic standards-MS-DIAL	5.52	213.0765	[M+H]+	Benzene and substituted derivatives	79.2	Methyl 4-Hydroxy-3,5-Dimethoxybenzoate	C10H12O5	181.0477,154.0611,139.0377	Level 2	70164
129	Authentic standards-MS-DIAL	30.73	391.2826	[M+H]+	Benzene and substituted derivatives	95.4	Dioctyl Phthalate	C24H38O4	279.1594,167.0336,149.0228,71.0855	Level 2	8346
130	Authentic standards-MS-DIAL	11.60	259.0965	[M-H]-	Benzene and substituted derivatives	89.3	Peucenin	C15H16O4	204.0426,134.0361	Level 2	68477

131	Authentic standards-MS-DIAL	6.12	461.1408	[M+Na]+	Benzene and substituted derivatives	84.4	Monnieriside G	C21H26O10	229.0872,203.0322	Level 2	23899994
132	Authentic standards-MS-DIAL	3.20	327.1059	[M-H]-	Benzene and substituted derivatives	76.7	1-(4-Hydroxyphenyl)-3-[(2R,3R,4S,5S,6R)-3,4,5-Trihydroxy-6-(Hydroxymethyl)Oxan-2-Yl]Oxypropan-1-One	C15H20O8	147.0437,89.0235,59.0131	Level 2	85152469
133	Fiehn/Vaniya natural product library	6.34	427.1535	[M-H]-	Benzene and substituted derivatives	70.7	(2R,3S,4S,5R,6R)-2-[[(2R,3S,4S,5R)-3,4-Dihydroxy-5-(Hydroxymethyl)Oxolan-2-Yl]Oxymethyl]-6-[(E)-3-Phenylprop-2-Enoxy]Oxane-3,4,5-Triol	C20H28O10	161.0442,125.0223,89.0242,59.0138	Level 2	75130937
134	Authentic standards-MS-DIAL	5.38	599.1979	[M+H]+	Benzene and substituted derivatives	72.2	3-(4-Hydroxyphenyl)-1-[2,4,6-Trihydroxy-3,5-Bis[3,4,5-Trihydroxy-6-(Hydroxymethyl)Oxan-2-Yl]Phenyl]Propan-1-One	C27H34O15	563.1769,461.1454,383.1121	Level 2	74819290
135	BMDMS-NP	10.63	287.0903	[M+H]+	Benzene and substituted derivatives	79.1	Licochalcone B	C16H14O5	203.0421,147.0496,67.0583	Level 2	480788
136	Authentic standards-MS-DIAL	2.86	318.1544	[M+NH4]+	Benzene and substituted derivatives	85.4	(2S,3R,4S,5S,6R)-2-[4-(2-Hydroxyethyl)Phenoxy]-6-(Hydroxymethyl)Oxane-3,4,5-Triol	C14H20O7	145.0495,121.0646,85.0288	Level 2	14539961
137	Authentic standards-MS-DIAL	3.65	378.1761	[M+NH4]+	Benzene and substituted derivatives	77.4	(2R,3R,4S,5S,6R)-2-[3-Hydroxy-2-(4-Hydroxy-3-Methoxyphenyl)Propoxy]-6-(Hydroxymethyl)Oxane-3,4,5-Triol	C16H24O9	181.086,137.0594, 85.0269	Level 2	75130902
138	Authentic standards-MS-DIAL	4.51	521.2018	[M-H2O+H]+	Benzene and substituted derivatives	86.4	3-Hydroxy-1-(3-Hydroxy-4-Methoxyphenyl)-2-{4-[(1E)-3-Hydroxy-1-Propen-1-Yl]-2-Methoxyphenoxy}Propyl Beta-D-Glucopyranoside	C26H34O12	311.1266,163.0754,131.0491,103.0541	Level 2	56776174

139	GNPS	4.06	355.1025	[M+H]+	Benzene and substituted derivatives	94.6	(3R,5S)-4-[(E)-3-(3,4-Dihydroxyphenyl)Prop-2-Enoyl]Oxy-1,3,5-Trihydroxycyclohexane-1-Carboxylic Acid	C16H18O9	163.0479,145.0316,117.0374,89.0404	Level 2	58427569
140	RIKEN PlaSMA bio-MS/MS from plant tissues	5.16	401.1428	[M-H]-	Benzene and substituted derivatives	77.8	2-[[3,4-Dihydroxy-4-(Hydroxymethyl)Oxolan-2-Yl]Oxymethyl]-6-Phenylmethoxyoxane-3,4,5-Triol	C18H26O10	269.1011,161.0441,71.0135	Level 2	14079044
141	RIKEN PlaSMA bio-MS/MS from plant tissues	4.86	431.1545	[M-H]-	Benzene and substituted derivatives	77.1	2-[4,5-Dihydroxy-6-(Hydroxymethyl)-2-Phenylmethoxyoxan-3-Yl]Oxy-6-(Hydroxymethyl)Oxane-3,4,5-Triol	C19H28O11	269.1028,161.0467,101.0232	Level 2	73157713
142	Fiehn/Vaniya natural product library	5.12	269.1020	[M-H]-	Benzene and substituted derivatives	85.8	11,22-Dimethyl (6E,17E)-3,14-Dioxo-8,19-Bis({[(2S,3R,4S,5S,6R)-3,4,5-Trihydroxy-6-(Hydroxymethyl)Oxan-2-Yl]Oxy})-4,9,15,20-Tetraoxatricyclo	C13H18O6	113.0229,85.0298, 71.0128,59.013	Level 2	4984739
143	Fiehn/Vaniya natural product library	4.38	299.1116	[M-H]-	Benzene and substituted derivatives	95.1	Salidroside	C14H20O7	119.0516,101.0231,89.0241,71.0135,59.013	Level 2	159278
144	Fiehn/Vaniya natural product library	3.69	343.1397	[M-H]-	Benzene and substituted derivatives	77.0	2-[1-Hydroxy-1-(4-Methoxyphenyl)Propan-2-Yl]Oxy-6-(Hydroxymethyl)Oxane-3,4,5-Triol	C16H24O8	101.0232,71.0128, 59.0138	Level 2	45360332
145	Fiehn/Vaniya natural product library	3.86	365.1209	[M+Na]+	Benzene and substituted derivatives	87.4	Coniferin	C16H22O8	203.0534,185.0402,	Level 2	3496897

146	Fiehn/Vaniya natural product library	4.99	395.0945	[M+Na]+	Benzene and substituted derivatives	82.2	(2S,3S,4S,5R,6R)-6-(3-Benzoyloxy-2-Hydroxypropoxy)-3,4,5-Trihydroxyoxane-2-Carboxylic Acid	C16H20O10	261.0725,219.0626,162.998	Level 2	75130890
147	Fiehn/Vaniya natural product library	6.44	441.1735	[M-H]-	Benzene and substituted derivatives	81.5	Lusitanicoside	C21H30O10	133.0647,125.0244,71.0127,59.0137	Level 2	4484591
148	Fiehn/Vaniya natural product library	3.79	557.1829	[M+Na]+	Benzene and substituted derivatives	78.3	(2R,3S,4S,5R,6R)-2-(Hydroxymethyl)-6-[[(2R,3S,4S,5R,6S)-3,4,5-Trihydroxy-6-[4-[(E)-3-Hydroxyprop-1-Enyl]-2,6-Dimethoxyphenoxy]Oxan-2-Yl]Methoxy]Oxane-3,4,5-Triol	C23H34O14	347.0919,331.1031,245.0629	Level 2	72726494
149	GNPS-MS1	3.57	335.0755	[M-H]-	Benzene and substituted derivatives		1,3,4-Trihydroxy-5-(1-Oxoisochromen-4-Yl)Oxycyclohexane-1-Carboxylic Acid	C16H16O8	179.0352,135.0456,93.0347	Level 3	163074473
150	GNPS-MS1	4.80	382.1101	[M+H]+	Benzene and substituted derivatives		3-[[6-[Cyano(Phenyl)Methoxy]-3,4,5-Trihydroxyoxan-2-Yl]Methoxy]-3-Oxopropanoic Acid	C17H19NO9	203.0353,147.0450	Level 3	14259069
151	GNPS-MS1	5.51	392.1330	[M+H]+	Benzene and substituted derivatives		2-(4,8-Dimethoxyfuro[2,3-B]Quinolin-7-Yl)Oxy-6-Methyloxane-3,4,5-Triol	C19H21NO8	269.0823,203.0338,60.0442	Level 3	3518320
152	GNPS-MS1	5.19	535.1989	[M-H]-	Benzene and substituted derivatives		2-[2,6-Dimethoxy-4-[2-[3,4,5-Trihydroxy-6-(Hydroxymethyl)Oxan-2-Yl]Oxypropyl]Phenoxy]-6-(Hydroxymethyl)Oxane-3,4,5-Triol	C23H36O14	475.1813,329.123, 59.0137	Level 3	78385405
153	GNPS-MS1	5.16	543.1672	[M-H]-	Benzene and substituted derivatives		Methyl 4-[2-[2-(3,4-Dihydroxyphenyl)Ethoxy]-2-Oxoethyl]-3-(Hydroxymethyl)-2-[3,4,5-Trihydroxy-6-	C24H32O14	269.0995,141.0167,59.0137	Level 3	163101231

						(Hydroxymethyl)Oxan-2-Yl]Oxy-3,4-Dihydro-2H-Pyran-5-Carboxylate				
154	GNPS-MS1	5.20	555.1672	[M+H]+	Benzene and substituted derivatives	[3,4,5-Trihydroxy-6-[(7-Methoxy-2-Oxo-10-Phenyl-3-Oxatricyclo[7.3.1.05,13]Trideca-1(13),5,7,9,11-Pentaen-8-Yl)Oxy]Oxan-2-Yl]Methyl N-Carbamoylcarbamate	C27H26N2O11	395.0588,233.0265	Level 3	85302618
155	GNPS-MS1	12.65	709.2227	[M-H]-	Benzene and substituted derivatives	[2-[3,4-Dihydroxy-6-(Hydroxymethyl)-5-[3,4,5-Trihydroxy-6-(Hydroxymethyl)Oxan-2-Yl]Oxyoxan-2-Yl]Oxy-4-Hydroxy-2,5-Bis(Hydroxymethyl)Oxolan-3-Yl] 3-(4-Hydroxy-3,5-Dimethoxyphenyl)Prop-2-Enoate	C29H42O20	471.1774,193.0501	Level 3	85237657
156	GNPS-MS1	4.80	903.2773	[M+H]+	Benzene and substituted derivatives	1-[6-[[3,5-Dihydroxy-6-(Hydroxymethyl)-4-[3,4,5-Trihydroxy-6-[[3,4,5-Trihydroxy-6-(Hydroxymethyl)Oxan-2-Yl]Oxymethyl]Oxan-2-Yl]Oxyoxan-2-Yl]Oxymethyl]-3,4,5-Trihydroxyoxan-2-Yl]Oxy-8-Hydroxy-3-Methylanthracene-9,10-Dione	C39H50O24	611.1594,303.0497.85.0286	Level 3	14213508
157	GNPS-MS1	4.44	933.2844	[M+H]+	Benzene and substituted derivatives	1-[6-[[3,5-Dihydroxy-6-(Hydroxymethyl)-4-[3,4,5-Trihydroxy-6-[[3,4,5-Trihydroxy-6-(Hydroxymethyl)Oxan-2-Yl]Oxymethyl]Oxan-2-Yl]Oxyoxan-2-Yl]Oxymethyl]-3,4,5-Trihydroxyoxan-2-	C40H52O25	625.1752,479.118, 317.0652	Level 3	85084774

							Yl]Oxy-8-Hydroxy-3-Methoxy-6-Methylanthracene-9,10-Dione				
158	Authentic standards-MS-DIAL	14.83	205.1585	[M-H]-	Benzene and substituted derivatives	93.1	4-Octylphenol	C14H22O	189.1273	Level 2	15730
159	GNPS	5.50	343.1178	[M+H]+	Benzene and substituted derivatives	78.9	1,3-Cyclobutanedicarboxylic Acid, 2,4-Bis(4-Hydroxyphenyl)-, Monomethyl Ester	C19H18O6	325.1081,2565.0862,91.0548	Level 2	56776249
160	GNPS-MS1	3.09	517.1539	[M+H]+	Benzene and substituted derivatives		[6,7-Dihydroxy-7-(Hydroxymethyl)-1-[3,4,5-Trihydroxy-6-(Hydroxymethyl)Oxan-2-Yl]Oxy-4A,5,6,7A-Tetrahydro-1H-Cyclopenta[C]Pyran-5-Yl] 3,4-Dihydroxybenzoate	C22H28O14	325.0905,163.0392	Level 3	51136578
161	GNPS-MS1	4.84	547.1400	[M+H]+	Benzene and substituted derivatives		Methyl 4'-(4-Hydroxybenzoyl)-5'-Oxo-1-[3,4,5-Trihydroxy-6-(Hydroxymethyl)Oxan-2-Yl]Oxyspiro[4A,7A-Dihydro-1H-Cyclopenta[C]Pyran-7,2'-Furan]-4-Carboxylate	C26H26O13	379.081,203.0338	Level 3	73812729
162	Authentic standards-MS-DIAL	4.61	377.1455	[M+H]+	Benzene and substituted derivatives	87.6	(-)-Riboflavin	C17H20N4O6	243.086,198.0665, 172.0874	Level 2	1072

163	GNPS	4.64	269.1017	[M-H]-	Benzene and substituted derivatives	90.5	(2R,3S,4S,5R,6R)-2-(Hydroxymethyl)-6-Phenylmethoxyoxane-3,4,5-Triol	C13H18O6	101.0231，71.0127，59.0137	Level 2	188977
164	GNPS	10.13	293.1741	[M-H]-	Benzene and substituted derivatives	93.5	5-Hydroxy-1-(4-Hydroxy-3-Methoxyphenyl)Decan-3-One	C17H26O4	236.1035,221.1557,192.1188	Level 2	3473
165	GNPS	5.41	303.0133	[M+H]+	Benzene and substituted derivatives	88.5	6,7,13,14-Tetrahydroxy-2,9-Dioxatetracyclo[6.6.2.04,16.011,15]Hexadeca-1(15),4,6,8(16),11,13-Hexaene-3,10-Dione	C14H6O8	257.0452,229.049, 201.0542,137.0238	Level 2	5281855
166	Authentic standards-MS-DIAL	6.00	321.1533	[M+CH3COO]-	Carbohydrates and carbohydrate conjugates	84.7	(2R,3R,4S,5S,6R)-2-[(Z)-Hex-3-Enoxy]-6-(Hydroxymethyl)Oxane-3,4,5-Triol	C12H22O6	89.0232,59.0137	Level 2	73981610
167	Authentic standards-MS-DIAL	3.21	351.1289	[M+CH3COO]-	Carbohydrates and carbohydrate conjugates	79.8	(3R)-4,4-Dimethyl-3-[(2S,3R,4S,5S,6R)-3,4,5-Trihydroxy-6-(Hydroxymethyl)Oxan-2-Yl]Oxyoxolan-2-One	C12H20O8	113.024,59.0138	Level 2	14701560
168	Authentic standards-MS-DIAL	3.53	387.1643	[M-H]-	Carbohydrates and carbohydrate conjugates	81.0	12:4+3O Fatty Acyl Hexoside	C18H28O9	163.1139,59.0138	Level 2	4480898
169	GNPS	6.53	471.2197	[M+Na]+	Carbohydrates and carbohydrate conjugates	83.2	2-(3,7-Dimethylocta-1,6-Dien-3-Yloxy)-6-[(3,4,5-Trihydroxyoxan-2-Yl)Oxymethyl]Oxane-3,4,5-Triol	C21H36O10	335.0952,203.0602	Level 2	73410547
170	Fiehn/Vaniya natural product library	5.90	331.1714	[M-H]-	Carbohydrates and carbohydrate conjugates	86.0	(3R,4S,5S,6R)-2-(6-Hydroxy-2,6-Dimethylocta-2,7-Dienoxy)-6-(Hydroxymethyl)Oxane-3,4,5-Triol	C16H28O7	113.0249,101.0241,85.0288,71.0135, 59.013	Level 2	72970728
171	Authentic standards-MS-DIAL	0.73	239.0758	[M+CH3COO]-	Carbohydrates and carbohydrate conjugates	86.3	Cyclohexane-1,2,3,4,5,6-Hexol	C6H12O6	179.0552,87.0083, 59.0138	Level 2	892

172	Authentic standards-MS-DIAL	0.78	262.1289	[M+H]+	Carbohydrates and carbohydrate conjugates	70.4	Mycosporine Serinol	C11H19NO6	244.117,216.1249, 198.1134	Level 2	442866
173	Authentic standards-MS-DIAL	5.26	353.0847	[M-H]-	Carbohydrates and carbohydrate conjugates	86.6	Neochlorogenic Acid	C16H18O9	191.0555,179.0338,135.0455	Level 2	348159
174	Authentic standards-MS-DIAL	2.98	369.1153	[M+Na]+	Carbohydrates and carbohydrate conjugates	87.9	D-Glucopyranoside, (4-Hydroxy-3,5-Dimethoxyphenyl)Methyl	C15H22O9	207.063,185.0415	Level 2	51136354
175	Authentic standards-MS-DIAL	6.64	391.1958	[M+CH3COO]-	Carbohydrates and carbohydrate conjugates	88.6	2-(Hydroxymethyl)-6-[[(1R,4S)-2,2,4-Trimethyl-3-Oxabicyclo[2.2.2]Octan-5-Yl]Oxy]Oxane-3,4,5-Triol	C16H28O7	331.1721,161.0465,59.0159	Level 2	73815050
176	Authentic standards-MS-DIAL	0.82	401.1286	[M+CH3COO]-	Carbohydrates and carbohydrate conjugates	94.4	Trehalose	C12H22O11	341.1131,179.0579,119.0369,89.0253, 71.0145,59.0161	Level 2	1143
177	Authentic standards-MS-DIAL	4.31	480.1467	[M+Na]+	Carbohydrates and carbohydrate conjugates	86.1	(2S)-{[6-O-(Beta-D-Glucopyranosyl)-Beta-D-Glucopyranosyl]Oxy}(Phenyl)Acetonitrile	C20H27NO11	374.112,347.094	Level 2	441462
178	Authentic standards-MS-DIAL	1.24	503.1603	[M-H]-	Carbohydrates and carbohydrate conjugates	94.4	D-(+)-Raffinose	C18H32O16	221.0648,179.0555,89.0235,	Level 2	439242
179	Authentic standards-MS-DIAL	12.59	614.3313	[M-H]-	Carbohydrates and carbohydrate conjugates	84.5	[(2R)-2-Hydroxy-3-[Hydroxy-[2-[[3,4,5-Trihydroxy-6-(Hydroxymethyl)Oxan-2-Yl]Amino]Ethoxy]Phosphoryl]Oxypropyl] Hexadecanoate	C27H54NO12P	452.2769,255.2319	Level 2	139292086
180	GNPS	2.22	311.0723	[M+Na]+	Carbohydrates and carbohydrate conjugates	72.4	2-Methyl-3-[3,4,5-Trihydroxy-6-(Hydroxymethyl)Oxan-2-Yl]Oxypyran-4-One	C12H16O8	185.0416149.0208	Level 2	14774602

181	Fiehn HILIC	0.82	341.1089	[M-H]-	Carbohydrates and carbohydrate conjugates	99.1	Sucrose	C12H22O11	179.0582,119.036, 101.0245,89.031, 71.0163,59.0185	Level 2	1115
182	Fiehn/Vaniya natural product library	0.75	527.1573	[M+Na]+	Carbohydrates and carbohydrate conjugates	90.3	Melezitose	C18H32O16	365.1068,347.0968	Level 2	220003
183	Authentic standards-MS-DIAL	7.51	447.2200	[M-H]-	Carbohydrates and carbohydrate conjugates	79.2	2-(3,7-Dimethylocta-2,6-Dienoxy)-6-[(3,4,5-Trihydroxyoxan-2-Yl)Oxymethyl]Oxane-3,4,5-Triol	C21H36O10	315.18,161.0427, 101.024,	Level 2	73717656
184	Authentic standards-MS-DIAL	7.69	507.2395	[M+CH3COO]-	Carbohydrates and carbohydrate conjugates	70.9	(2R,3S,4S,5R,6R)-2-[[(2R,3R,4R)-3,4-Dihydroxy-4-(Hydroxymethyl)Oxolan-2-Yl]Oxymethyl]-6-[(2E)-3,7-Dimethylocta-2,6-Dienoxy]Oxane-3,4,5-Triol	C21H36O10	447.2234,161.0465,89.0241,59.0137	Level 2	73815004
185	Fiehn/Vaniya natural product library	5.89	489.1361	[M+Na]+	Carbohydrates and carbohydrate conjugates	82.4	Agnuside	C22H26O11	327.083,203.0519	Level 2	3512643
186	GNPS-MS1	3.21	455.1555	[M+H]+	Carbohydrates and carbohydrate conjugates		Methyl 4'-Ethyl-5'-Oxo-1-[3,4,5-Trihydroxy-6-(Hydroxymethyl)Oxan-2-Yl]Oxyspiro[4A,7A-Dihydro-1H-Cyclopenta[C]Pyran-7,2'-Furan]-4-Carboxylate	C21H26O11	317.0869	Level 3	14190700

187	GNPS-MS1	4.45	787.2287	[M+H]+	Carbohydrates and carbohydrate conjugates		[4,5-Dihydroxy-6-(Hydroxymethyl)-2-[[6-(Hydroxymethyl)-2-Oxo-3,9-Dioxatricyclo[5.3.1.04,11]Undeca-1(10),5-Dien-8-Yl]Oxy]Oxan-3-Yl] 7-(Acetyloxymethyl)-5-Hydroxy-1-[3,4,5-Trihydroxy-6-(Hydroxymethyl)Oxan-2-Yl]Oxy-1,4A,5,7A-Tetrahydrocyclopenta[C]Pyran-4-Carboxylate	C34H42O21	641.1805,479.127, 317.076	Level 3	163050216
188	Authentic standards-MS-DIAL	2.86	267.0714	[M-H]-	Carbohydrates and carbohydrate conjugates	93.3	Inosine	C10H12N4O5	135.0311,108.021,92.0254	Level 2	135398641
189	Authentic standards-MS-DIAL	1.11	268.1053	[M+H]+	Carbohydrates and carbohydrate conjugates	95.0	Adenosine	C10H13N5O4	136.0666,119.0339	Level 2	191
190	Authentic standards-MS-DIAL	2.91	282.0828	[M-H]-	Carbohydrates and carbohydrate conjugates	93.4	Guanosine	C10H13N5O5	150.0425,133.0153,108.02	Level 2	135398635
191	Authentic standards-MS-DIAL	0.78	332.1333	[M+H]+	Carbohydrates and carbohydrate conjugates	74.5	2-[[5-Hydroxy-4-(Hydroxymethyl)-6-Methylpyridin-3-Yl]Methoxy]-6-(Hydroxymethyl)Oxane-3,4,5-Triol	C14H21NO8	314.1229,152.0703,108.0806	Level 2	3321053
192	GNPS	4.31	455.1155	[M+Na]+	Carbohydrates and carbohydrate conjugates	91.2	2-Methyl-4-Oxo-4H-Pyran-3-Yl 6-O-(4-Carboxy-3-Hydroxy-3-Methylbutanoyl)-Beta-D-Glucopyranoside	C18H24O12	393.1138,329.0842,149.0197	Level 2	73880679
193	GNPS	3.84	341.1225	[M-H]-	Carbohydrates and carbohydrate conjugates	84.7	(3S,4R,5R)-2-(Hydroxymethyl)-3-[(2R,3R,4S,5S,6R)-3,4,5-Trihydroxy-6-(Hydroxymethyl)Oxan-2-Yl]Oxyoxane-2,4,5-Triol	C12H22O11	181.0932,85.2966	Level 2	54301978

194	GNPS	1.03	401.1280	[M-H]-	Carbohydrates and carbohydrate conjugates	97.4	(2R,3S,4S,5R,6R)-2-(Hydroxymethyl)-6-[(2R,3R,4S,5S,6R)-3,4,5-Trihydroxy-6-(Hydroxymethyl)Oxan-2-Yl]Oxyoxane-3,4,5-Triol	C12H22O11	89.0235,59.0132	Level 2	7427
195	GNPS	7.02	491.2092	[M-H]-	Carbohydrates and carbohydrate conjugates	73.1	[3,4,5-Trihydroxy-6-[[3,4,5-Trihydroxy-6-(Hydroxymethyl)Oxan-2-Yl]Oxymethyl]Oxan-2-Yl] 2,6,6-Trimethylcyclohexene-1-Carboxylate	C22H36O12	323.1047,167.1062	Level 2	45783214
196	GNPS	26.38	637.3010	[M+Na]+	Carbohydrates and carbohydrate conjugates	86.5	5-Amino-2-(Aminomethyl)-6-[4,6-Diamino-2-[4-[3-Amino-6-(Aminomethyl)-4,5-Dihydroxyoxan-2-Yl]Oxy-3-Hydroxy-5-(Hydroxymethyl)Oxolan-2-Yl]Oxy-3-Hydroxycyclohexyl]Oxyoxane-3,4-Diol	C23H46N6O13	581.2407,393.0832	Level 2	4454
197	GNPS	0.82	683.2249	[2M-H]-	Carbohydrates and carbohydrate conjugates	76.0	2-(Hydroxymethyl)-6-[[3,4,5-Trihydroxy-5-(Hydroxymethyl)Oxolan-2-Yl]Methoxy]Oxane-3,4,5-Triol	C12H22O11	161.047,113.0252	Level 2	3749604
198	Authentic standards-MS-DIAL	3.75	203.0817	[M-H]-	Carboxylic acids and derivatives	96.3	Tryptophan	C11H12N2O2	159.0905,142.0673,116.0516,74.0259	Level 2	1148
199	Authentic standards-MS-DIAL	2.66	220.1168	[M+H]+	Carboxylic acids and derivatives	97.0	Pantothenic Acid	C9H17NO5	202.1078,184.0963,124.0754,90.0551	Level 2	6613
200	Authentic standards-MS-DIAL	5.41	225.1480	[M+H]+	Carboxylic acids and derivatives	75.6	Methyl 2-[(1R,2R)-3-Oxo-2-[(Z)-Pent-2-Enyl]Cyclopentyl]Acetate	C13H20O3	207.1362,151.1122,93.0699	Level 2	5281929

201	Authentic standards-MS-DIAL	2.66	245.0931	[M-H]-	Carboxylic acids and derivatives	96.2	N-Acetyltryptophan	C13H14N2O3	203.0839,116.0494,98.0251,74.0242, 58.0296	Level 2	2002
202	Authentic standards-MS-DIAL	26.90	257.2467	[M+H]+	Carboxylic acids and derivatives	74.2	14-Methylpentadecanoic Acid	C16H32O2	89.0596,71.0855, 57.07	Level 2	36247
203	Authentic standards-MS-DIAL	6.88	261.0753	[M+Cl]-	Carboxylic acids and derivatives	92.9	Carnosine	C9H14N4O3	203.0348,189.0186,175.0404,161.0242	Level 2	9369
204	Authentic standards-MS-DIAL	23.47	279.2320	[M+H]+	Carboxylic acids and derivatives	91.0	(9Z,12Z,15Z)-Octadeca-9,12,15-Trienoic Acid	C18H30O2	173.1314,109.1005,95.0859,81.0704, 67.0574	Level 2	5280934
205	Authentic standards-MS-DIAL	25.41	281.2469	[M+H]+	Carboxylic acids and derivatives	90.0	Linoleic Acid	C18H32O2	263.2345,245.2247,133.101,81.0695, 69.0697,55.0536	Level 2	5280450
206	Authentic standards-MS-DIAL	27.53	283.2606	[M+H]+	Carboxylic acids and derivatives	87.9	(Z)-Octadec-9-Enoic Acid	C18H34O2	265.2532,247.241, 121.0997,69.0698	Level 2	445639
207	Authentic standards-MS-DIAL	18.80	295.2260	[M+H]+	Carboxylic acids and derivatives	76.3	13-Oxoode	C18H30O3	277.2177,179.1431,93.0698	Level 2	25791066
208	Authentic standards-MS-DIAL	11.45	295.2262	[M-H]-	Carboxylic acids and derivatives	95.9	9-Hode	C18H32O3	277.2159,171.1021,123.1174	Level 2	5282944
209	Authentic standards-MS-DIAL	15.42	303.2296	[M-H]-	Carboxylic acids and derivatives	86.9	Arachidonic Acid	C20H32O2	259.2393,231.2127,59.0121	Level 2	444899

210	Authentic standards-MS-DIAL	18.24	325.2355	[M+H]+	Carboxylic acids and derivatives	74.8	Allo-Protolichesterinic Acid	C19H32O4	233.1894,135.1157,67.0542	Level 2	71443326
211	Authentic standards-MS-DIAL	4.93	369.1186	[M+H]+	Carboxylic acids and derivatives	90.9	(3R,5R)-1,3,5-Trihydroxy-4-[(E)-3-(4-Hydroxy-3-Methoxyphenyl)Prop-2-Enoyl]Oxycyclohexane-1-Carboxylic Acid	C17H20O9	177.0559,145.0292,117.0342,89.0385	Level 2	10177048
212	Authentic standards-MS-DIAL	22.81	403.2309	[M+H]+	Carboxylic acids and derivatives	82.7	Acetyl Tributyl Citrate	C20H34O8	185.0811,157.0126,129.0183,68.9965	Level 2	6505
213	Authentic standards-MS-DIAL	10.48	409.2181	[M+Na]+	Carboxylic acids and derivatives	82.1	2-[5-[2-[2-[5-(2-Hydroxypropyl)Oxolan-2-Yl]Propanoyloxy]Propyl]Oxolan-2-Yl]Propanoic Acid	C20H34O7	225.1659,211.1326	Level 2	23815283
214	Authentic standards-MS-DIAL	3.86	413.1419	[M+H]+	Carboxylic acids and derivatives	81.3	N6-Threonylcarbamoyladenosine	C15H20N6O8	281.0989,162.0396,136.0608	Level 2	161466
215	Authentic standards-MS-DIAL	5.26	515.1176	[M-H]-	Carboxylic acids and derivatives	93.8	(1S,3R,4S,5R)-3,5-Bis({[(2E)-3-(3,4-Dihydroxyphenyl)Prop-2-Enoyl]Oxy})-1,4-Dihydroxycyclohexane-1-Carboxylic Acid	C25H24O12	353.0876,191.0555,179.0338,135.0443	Level 2	60150332
216	Authentic standards-MS-DIAL	5.96	539.1158	[M+Na]+	Carboxylic acids and derivatives	85.5	1,3-Dicaffeoylquinic Acid	C25H24O12	377.0854,359.072, 163.0377	Level 2	205954
217	Authentic standards-MS-DIAL	12.12	583.2532	[M+H]+	Carboxylic acids and derivatives	87.7	Biliverdin	C33H34N4O6	297.1228,253.135, 227.1277	Level 2	251
218	Authentic standards-MS-DIAL	27.44	593.2740	[M+H]+	Carboxylic acids and derivatives	88.2	Pheophorbide A	C35H36N4O5	533.2582,505.2293,460.2254	Level 2	167186

219	RIKEN PlaSMA bio-MS/MS from plant tissues	6.82	213.1484	[M-H]-	Carboxylic acids and derivatives	84.6	12-Hydroxydodec-3-Enoic Acid	C12H22O3	195.1377,183.1387	Level 2	54281924
220	RIKEN PlaSMA bio-MS/MS from plant tissues	1.06	274.0916	[M+H]+	Carboxylic acids and derivatives	82.6	5-Oxo-1-[[2,3,4-Trihydroxy-5-(Hydroxymethyl)Oxolan-2-Yl]Methyl]Pyrrolidine-2-Carboxylic Acid	C11H15NO7	238.0729,192.0647,130.0504,97.0283	Level 2	139292107
221	RIKEN PlaSMA bio-MS/MS from plant tissues	0.81	294.1550	[M+H]+	Carboxylic acids and derivatives	85.7	N-Fructosyl Isoleucine	C12H23NO7	276.1454,203.1405,132.1014,86.0966	Level 2	139292115
222	RIKEN PlaSMA bio-MS/MS from plant tissues	2.12	328.1382	[M+H]+	Carboxylic acids and derivatives	76.1	N-Fructosyl Phenylalanine	C15H21NO7	310.1302,292.1187,166.0867,120.0809	Level 2	85152457
223	RIKEN PlaSMA bio-MS/MS from plant tissues	3.65	337.0909	[M-H]-	Carboxylic acids and derivatives	85.7	Coumaroyl Quinic Acid	C16H18O8	191.0556,173.0457,93.0347	Level 2	53420248
224	RIKEN PlaSMA bio-MS/MS from plant tissues	4.59	367.1010	[M-H]-	Carboxylic acids and derivatives	85.7	Feruloyl Quinic Acid	C17H20O9	191.0558,134.0364,85.0298	Level 2	4635494
225	Fiehn/Vaniya natural product library	14.12	227.0696	[M+K]+	Carboxylic acids and derivatives	74.4	Nonanedioic Acid	C9H16O4	171.0424,128.0618	Level 2	2266

226	Fiehn/Vaniya natural product library	4.16	367.1030	[M-H]-	Carboxylic acids and derivatives	94.9	3-O-Feruloylquinic Acid	C17H20O9	191.0596,173.0457,134.0385,111.0449, 93.0347	Level 2	9799386
227	Fiehn/Vaniya natural product library	4.93	391.0992	[M+Na]+	Carboxylic acids and derivatives	94.7	Methyl Chlorogenate	C17H20O9	373.0884,215.0528,197.0403,177.0545, 149.0255	Level 2	73412063
228	Fiehn/Vaniya natural product library	6.53	432.1824	[M+NH4]+	Carboxylic acids and derivatives	72.2	3-Oxo-3-[[(2R,3S,4S,5R,6S)-3,4,5-Trihydroxy-6-(4-Hydroxy-5-Methyl-2-Propan-2-Ylphenoxy)Oxan-2-Yl]Methoxy]Propanoic Acid	C19H26O10	231.0482,127.0379,85.0268	Level 2	75061268
229	GNPS	6.77	275.2005	[M-H2O+H]+	Carboxylic acids and derivatives	84.7	(10E,12Z,15Z)-9-Oxooctadeca-10,12,15-Trienoic Acid	C18H28O3	257.2021,147.1172,133.1	Level 2	11380794
230	GNPS	8.97	315.2535	[M+H]+	Carboxylic acids and derivatives	87.8	Dibutyl Decanedioate	C18H34O4	279.2316,95.0859, 81.0687	Level 2	7986
231	GNPS	7.16	327.2186	[M-H]-	Carboxylic acids and derivatives	91.3	9,12,13-Trihydroxyoctadeca-10,15-Dienoic Acid	C18H32O5	211.1338,183.1384	Level 2	5312876
232	Authentic standards-MS-DIAL	18.02	279.2306	[M-2H2O+H]+	Carboxylic acids and derivatives	90.2	8-Hydroxy-8-(3-Octyloxiran-2-Yl)Octanoic Acid	C18H34O4	161.221,109.1006, 95.0859,81.0696, 67.055	Level 2	53851542
233	Authentic standards-MS-DIAL	15.63	293.2105	[M+H]+	Carboxylic acids and derivatives	85.7	8-[3-Oxo-2-[(E)-Pent-2-Enyl]Cyclopenten-1-Yl]Octanoic Acid	C18H28O3	275.2014,257.1866,119.0851,95.085	Level 2	591115
234	Authentic standards-MS-DIAL	10.50	293.2111	[M-H]-	Carboxylic acids and derivatives	93.5	13-Hotre	C18H30O3	75.2014,223.1345, 195.1375,59.0136	Level 2	10469728

235	Authentic standards-MS-DIAL	19.55	295.2260	[M+H]+	Carboxylic acids and derivatives	80.6	9-Oxooctadeca-10,12-Dienoic Acid	C18H30O3	277.2144,151.1119,67.0534	Level 2	193923
236	Authentic standards-MS-DIAL	13.45	297.2412	[M-H]-	Carboxylic acids and derivatives	92.5	8-(3-Octyloxiran-2-Yl)Octanoic Acid	C18H34O3	279.2309,155.107	Level 2	15868
237	Authentic standards-MS-DIAL	8.44	311.2216	[M-H]-	Carboxylic acids and derivatives	83.5	9-Hydroperoxyoctadeca-10,12-Dienoic Acid	C18H32O4	293.2121,171.1021	Level 2	1928
238	Authentic standards-MS-DIAL	13.04	313.2362	[M+H]+	Carboxylic acids and derivatives	72.6	(9Z,12E)-15,16-Dihydroxyoctadeca-9,12-Dienoic Acid	C18H32O4	149.133,133.1011,121.0998,81.0687	Level 2	54038189
239	Authentic standards-MS-DIAL	6.85	327.2154	[M-H]-	Carboxylic acids and derivatives	89.8	(10E,15Z)-9,12,13-Trihydroxyoctadeca-10,15-Dienoic Acid	C18H32O5	291.1972,229.1423,211.1326,171.1009	Level 2	72732143
240	Authentic standards-MS-DIAL	7.28	329.2319	[M-H]-	Carboxylic acids and derivatives	93.5	(Z)-5,8,11-Trihydroxyoctadec-9-Enoic Acid	C18H34O5	311.2226,229.1436,211.1353,171.1021	Level 2	74178128
241	Authentic standards-MS-DIAL	24.33	331.2830	[M+H]+	Carboxylic acids and derivatives	86.3	Ceratodictyol	C19H38O4	313.2733,239.2366,95.0839,85.0999	Level 2	139292023
242	MassBank	9.78	311.2215	[M-H]-	Carboxylic acids and derivatives	70.1	13-Hpode	C18H32O4	293.2119,211.1324,171.1033	Level 2	1928
243	GNPS	8.47	275.1989	[M-H2O+H]+	Carboxylic acids and derivatives	87.5	8-{(1S,5R)-4-Oxo-5-[(2Z)-2-Penten-1-Yl]-2-Cyclopenten-1-Yl}Octanoic Acid	C18H28O3	257.1895,133.1023,105.0695,91.0537	Level 2	188366
244	RIKEN PlaSMA bio-	8.97	265.1791	[M-H]-	Carboxylic acids and derivatives	81.2	6-(3-Oxo-2-Pent-2-Enylcyclopentyl)Hexanoic Acid	C16H26O3	247.1688,183.1027,155.1072	Level 2	54432406

	MS/MS from plant tissues										
245	RIKEN PlaSMA bio-MS/MS from plant tissues	11.75	293.2109	[M-H]-	Carboxylic acids and derivatives	81.8	8-(3-Octa-2,5-Dienyloxiran-2-Yl)Octanoic Acid	C18H30O3	275.1996,211.1337,183.1384	Level 2	111585
246	RIKEN PlaSMA bio-MS/MS from plant tissues	11.42	293.2110	[M-H]-	Carboxylic acids and derivatives	81.1	9-Hydroxyoctadeca-10,12,15-Trienoic Acid	C18H30O3	275.2015,235.1708,183.1412	Level 2	53394275
247	RIKEN PlaSMA bio-MS/MS from plant tissues	2.79	305.0664	[M-H]-	Carboxylic acids and derivatives	73.9	2-[3-Oxo-2-(5-Sulfooxypent-2-Enyl)Cyclopentyl]Acetic Acid	C12H18O7S	225.1128,96.959, 59.0131	Level 2	75075824
248	RIKEN PlaSMA bio-MS/MS from plant tissues	7.96	307.1896	[M-H]-	Carboxylic acids and derivatives	83.9	16-Hydroxy-9-Oxooctadeca-10,12,14-Trienoic Acid	C18H28O4	235.1317,185.1184,121.0657,65.0391	Level 2	57137439
249	RIKEN PlaSMA bio-MS/MS from plant tissues	7.59	309.2040	[M-H]-	Carboxylic acids and derivatives	79.0	9-Oxo-11-(3-Pentyloxiran-2-Yl)Undec-10-Enoic Acid	C18H30O4	291.1952,185.117,171.102,137.096	Level 2	53394018
250	RIKEN PlaSMA bio-MS/MS from plant tissues	8.66	309.2042	[M-H]-	Carboxylic acids and derivatives	77.9	8-[3-(3-Oxooct-1-Enyl)Oxiran-2-Yl]Octanoic Acid	C18H30O4	291.1937,209.1155,171.1021,99.0814	Level 2	74218382
251	RIKEN PlaSMA bio-	7.82	309.2043	[M-H]-	Carboxylic acids and derivatives	81.5	9-Hydroperoxyoctadeca-10,12,15-Trienoic Acid	C18H30O4	291.1956,251.1642,171.1022,137.0973	Level 2	25200982

	MS/MS from plant tissues										
252	RIKEN PlaSMA bio-MS/MS from plant tissues	9.46	313.2369	[M-H]-	Carboxylic acids and derivatives	75.5	12,13-Dihydroxyoctadec-9-Enoic Acid	C18H34O4	277.2159,183.1358,99.0804	Level 2	53394649
253	RIKEN PlaSMA bio-MS/MS from plant tissues	7.63	329.2317	[M-H]-	Carboxylic acids and derivatives	85.7	9,12,13-Trihydroxyoctadec-10-Enoic Acid	C18H34O5	199.1328,169.1224,99.0823	Level 2	153001
254	RIKEN PlaSMA bio-MS/MS from plant tissues	5.48	343.2102	[M-H]-	Carboxylic acids and derivatives	77.2	9-Hydroxy-11-[3-Hydroxy-5-(1-Hydroxypropyl)Oxolan-2-Yl]Undec-10-Enoic Acid	C18H32O6	201.1128,171.1012,135.0787	Level 2	145994450
255	Fiehn/Vaniya natural product library	12.49	295.2259	[M-H]-	Carboxylic acids and derivatives	88.6	9-Hydroxy-10,12-Octadecadienoic Acid	C18H32O3	277.2191,195.1374,183.1397,113.0956	Level 2	1927
256	Fiehn/Vaniya natural product library	26.92	334.3062	[M+H]+	Carboxylic acids and derivatives	70.1	(2E,4E,12Z)-N-(2-Methylpropyl)Octadeca-2,4,12-Trienamide	C22H39NO	109.1005,95.0849,67.0533	Level 2	71346377
257	Authentic standards-MS-DIAL	2.18	325.0911	[M-H]-	Carboxylic acids and derivatives	89.9	(E)-3-[4-[(2S,3R,4S,5S,6R)-3,4,5-Trihydroxy-6-(Hydroxymethyl)Oxan-2-Yl]Oxyphenyl]Prop-2-Enoic Acid	C15H18O8	163.0417,119.0509	Level 2	73208527
258	Authentic standards-MS-DIAL	2.75	343.0987	[M-H]-	Carboxylic acids and derivatives	76.2	3-[3-Hydroxy-2-[(2S,3R,4S,5S,6R)-3,4,5-Trihydroxy-6-(Hydroxymethyl)Oxan-2-Yl]Oxyphenyl]Propanoic Acid	C15H20O9	181.0499,137.0609	Level 2	100640816

259	Authentic standards-MS-DIAL	5.40	379.1004	[M+Na]+	Carboxylic acids and derivatives	87.6	2-Glucosyloxy-4-Methoxycinnamic Acid	C16H20O9	335.113,271.0421	Level 2	74135933
260	Authentic standards-MS-DIAL	5.40	391.0989	[M+Na]+	Carboxylic acids and derivatives	78.0	3-[6-[(2S,3R,4S,5S,6R)-3,4,5-Trihydroxy-6-(Hydroxymethyl)Oxan-2-Yl]Oxy-1-Benzofuran-5-Yl]Propanoic Acid	C17H20O9	229.046,185.0466	Level 2	21592256
261	RIKEN PlaSMA bio-MS/MS from plant tissues	5.03	327.1059	[M-H]-	Carboxylic acids and derivatives	82.0	Dihydrocoumaroyl Hexoside	C15H20O8	165.0556,121.067	Level 2	73981563
262	GNPS-MS1	5.81	481.0975	[M+H]+	Carboxylic acids and derivatives		5-Hydroxy-4-[2-Hydroxy-6-[3,4,5-Trihydroxy-6-(Hydroxymethyl)Oxan-2-Yl]Oxybenzoyl]Benzene-1,3-Dicarboxylic Acid	C21H20O13	223.0489,218.0222,109.02281	Level 3	162890385
263	GNPS-MS1	4.22	553.1514	[M+H]+	Carboxylic acids and derivatives		4-(Carboxymethyl)-5-[2-[3-(4-Hydroxyphenyl)Prop-2-Enoyloxy]Ethylidene]-6-[3,4,5-Trihydroxy-6-(Hydroxymethyl)Oxan-2-Yl]Oxy-4H-Pyran-3-Carboxylic Acid	C25H28O14	348.097,178.0551,	Level 3	163033066
264	GNPS-MS1	6.63	911.1881	[M+H]+	Carboxylic acids and derivatives		1-[3,4-Dihydroxy-6-(Hydroxymethyl)-5-[5-Hydroxy-7-(Methylsulfanylcarbonyloxymethyl)-1-[3,4,5-Trihydroxy-6-(Hydroxymethyl)Oxan-2-Yl]Oxy-1,4A,5,7A-Tetrahydrocyclopenta[C]Pyran-4-Carbonyl]Oxyoxan-2-Yl]Oxy-5-Hydroxy-7-(Methylsulfanylcarbonyloxymethyl)-	C36H46O23S2	765.1308,603.077, 303.0497	Level 3	73820754

							1,4A,5,7A-Tetrahydrocyclopenta[C]Pyran-4-Carboxylic Acid			
265	GNPS	2.37	301.0903	[M+Na]+	Carboxylic acids and derivatives	77.6	4-(Beta-D-Glucopyranosyloxy)-2-Methylenebutanoic Acid	C11H18O8	203.0522,185.047, 139.0378	Level 2	17800830
266	Fiehn/Vaniya a natural product library	5.96	445.2062	[M-H]-	Carboxylic acids and derivatives	82.2	(Z)-2,6-Dimethyl-7-(4-Methyl-5-Oxooxolan-2-Yl)-3-[[3,4,5-Trihydroxy-6-(Hydroxymethyl)Oxan-2-Yl]Oxymethyl]Hept-5-Enoic Acid	C21H34O10	119.0344,89.0232, 71.0135,59.0145	Level 2	75110943
267	GNPS	4.65	355.1020	[M-H]-	Carboxylic acids and derivatives	72.4	(Z)-3-[4-Methoxy-2-[(2S,3R,4S,5S,6R)-3,4,5-Trihydroxy-6-(Hydroxymethyl)Oxan-2-Yl]Oxyphenyl]Prop-2-Enoic Acid	C16H20O9	193.0613,59.0137	Level 2	24094157
268	GNPS	4.05	371.0976	[M-H]-	Carboxylic acids and derivatives	90.7	(2S,3S,4S,5R,6R)-6-(3-Benzoyloxy-2-Hydroxypropoxy)-3,4,5-Trihydroxyoxane-2-Carboxylic Acid	C16H20O10	197.0424,167.0341,125.0244	Level 2	45783079
269	GNPS	5.36	373.1846	[M-H]-	Carboxylic acids and derivatives	72.3	2-[2-[(Z)-Pent-2-Enyl]-3-[3,4,5-Trihydroxy-6-(Hydroxymethyl)Oxan-2-Yl]Oxycyclopentyl]Acetic Acid	C18H30O8	101.0241,59.0137	Level 2	45360381
270	GNPS	4.59	445.2047	[M-H]-	Carboxylic acids and derivatives	86.6	(Z)-2,6-Dimethyl-7-(4-Methyl-5-Oxooxolan-2-Yl)-3-[[3,4,5-Trihydroxy-6-(Hydroxymethyl)Oxan-2-Yl]Oxymethyl]Hept-5-Enoic Acid	C21H34O10	403.1938,241.1428	Level 2	45359728

271	GNPS	4.68	515.1383	[M-H]-	Carboxylic acids and derivatives	87.7	(1S,3R,4R,5R)-1,3-Bis[[(E)-3-(3,4-Dihydroxyphenyl)Prop-2-Enoyl]Oxy]-4,5-Dihydroxycyclohexane-1-Carboxylic Acid	C25H24O12	353.0862,191.0559	Level 2	6474640
272	Authentic standards-MS-DIAL	2.91	223.0598	[M-H]-	Cinnamic acids and derivatives	87.5	Sinapic Acid	C11H12O5	208.0365,193.0142,164.0478,149.024,121.0316,93.033	Level 2	637775
273	Authentic standards-MS-DIAL	4.06	337.0903	[M+H]+	Cinnamic acids and derivatives	70.4	(3R,4R,5R)-5-[(E)-3-(3,4-Dihydroxyphenyl)Prop-2-Enoyl]Oxy-3,4-Dihydroxycyclohexene-1-Carboxylic Acid	C16H16O8	163.0392,145.0268,117.0321,89.0386	Level 2	5281762
274	Authentic standards-MS-DIAL	12.64	439.1390	[M-H2O+H]+	Cinnamic acids and derivatives	73.8	6-O-[(2E)-3-(4-Hydroxyphenyl)-2-Propenoyl]-1-O-[(2E)-3-Phenyl-2-Propenoyl]-Beta-D-Glucopyranose	C24H24O9	275.094,147.0447,119.0497	Level 2	26302443
275	RIKEN PlaSMA bio-MS/MS from plant tissues	2.26	341.0864	[M-H]-	Cinnamic acids and derivatives	87.1	Caffeic Acid Hexoside	C15H18O9	179.0344,135.0448	Level 2	4484225
276	Authentic standards-MS-DIAL	17.40	203.0343	[M+H]+	Coumarins and derivatives	82.2	Bergaptol	C11H6O4	159.0443,147.0436,131.048,91.0547	Level 2	5280371
277	Authentic standards-MS-DIAL	11.53	203.0333	[M+H]+	Coumarins and derivatives	95.9	Xanthotoxol	C11H6O4	175.0377,147.0437,131.0491,91.0547	Level 2	65090
278	Authentic standards-MS-DIAL	13.05	203.0694	[M+H]+	Coumarins and derivatives	73.0	7-Hydroxy-2,3-Dihydrocyclopenta[C]Chromen-4(1H)-One	C12H10O3	175.0387,147.0446,91.0546	Level 2	5322196
279 [a]	Authentic standards-MS-DIAL	9.53	207.0652	[M+H]+	Coumarins and derivatives	95.0	Citropen	C11H10O4	192.0411,164.0464,149.0232,121.0647,91.0547，77.0385	Level 1	2775

280	Authentic standards-MS-DIAL	18.96	217.0487	[M+H]+	Coumarins and derivatives	94.4	5-Methoxypsoralen	$C_{12}H_8O_4$	202.0252,174.0315,146.0363,118.0403	Level 2	2355
281	Authentic standards-MS-DIAL	18.19	217.0489	[M+H]+	Coumarins and derivatives	93.4	Xanthotoxin	$C_{12}H_8O_4$	202.0252,174.0303,161.0591,118.0403	Level 2	4114
282	Authentic standards-MS-DIAL	5.34	227.0698	[M-H2O+H]+	Coumarins and derivatives	93.5	8-(2-Hydroxypropan-2-Yl)Furo[2,3-H]Chromen-2-One	$C_{14}H_{12}O_4$	199.0402,184.0522,171.0452,128.0619	Level 2	160600
283	Authentic standards-MS-DIAL	7.02	231.1012	[M+H]+	Coumarins and derivatives	83.7	Osthenol	$C_{14}H_{14}O_3$	175.0389,147.0436,91.0537	Level 2	5320318
284	Authentic standards-MS-DIAL	15.41	245.1169	[M+H]+	Coumarins and derivatives	93.8	Osthol	$C_{15}H_{16}O_3$	189.0636,161.0603,131.0569,103.062	Level 2	10228
285	Authentic standards-MS-DIAL	9.98	247.0606	[M+H]+	Coumarins and derivatives	93.7	Isopimpinellin	$C_{13}H_{10}O_5$	232.0401,217.0176,189.0194,161.0231	Level 2	68079
286	Authentic standards-MS-DIAL	7.09	247.0959	[M+H]+	Coumarins and derivatives	94.9	(2S)-2-(2-Hydroxypropan-2-Yl)-2,3-Dihydrofuro[3,2-G]Chromen-7-One	$C_{14}H_{14}O_4$	229.0845,175.0389,147.0436,119.0476,91.0528	Level 2	334704
287	Authentic standards-MS-DIAL	10.56	261.1111	[M+H]+	Coumarins and derivatives	94.8	8-(2-Hydroxy-3-Methylbut-3-Enyl)-7-Methoxychromen-2-One	$C_{15}H_{16}O_4$	243.1027,189.0583,131.0502,103.054	Level 2	13343540
288	Authentic standards-MS-DIAL	16.05	271.0945	[M+H]+	Coumarins and derivatives	92.3	4-(3-Methylbut-2-Enoxy)Furo[3,2-G]Chromen-7-One	$C_{16}H_{14}O_4$	203.0336,147.0447,131.049,91.0537	Level 2	68081

289	Authentic standards-MS-DIAL	16.56	287.0906	[M-H2O+H]+	Coumarins and derivatives	96.1	(9-Hydroxy-8,8-Dimethyl-2-Oxo-9,10-Dihydropyrano[2,3-F]Chromen-10-Yl) Acetate	C16H16O6	245.0809,227.0701,175.0376,128.0618	Level 2	5320813
290	Authentic standards-MS-DIAL	15.31	301.1068	[M+H]+	Coumarins and derivatives	94.1	4-Methoxy-9-(3-Methylbut-2-Enoxy)Furo[3,2-G]Chromen-7-One	C17H16O5	233.0517,218.0278,162.0319	Level 2	98608
291	Authentic standards-MS-DIAL	6.87	322.1289	[M+NH4]+	Coumarins and derivatives	94.1	9-(2,3-Dihydroxy-3-Methylbutoxy)Furo[3,2-G]Chromen-7-One	C16H16O6	287.0913,269.0834,203.0349,147.0435	Level 2	328236
292	Authentic standards-MS-DIAL	21.75	327.1223	[M-H2O+H]+	Coumarins and derivatives	86.2	Isolaserpitin	C19H20O6	227.0699,83.0493, 55.0536	Level 2	511783
293	Authentic standards-MS-DIAL	15.97	329.1372	[M+H]+	Coumarins and derivatives	94.9	2-(7-Oxo-2,3-Dihydrofuro[3,2-G]Chromen-2-Yl)Propan-2-Yl (Z)-2-Methylbut-2-Enoate	C19H20O5	247.0951,229.0861,213.054,83.0485	Level 2	21670035
294	Authentic standards-MS-DIAL	10.54	339.0828	[M+Na]+	Coumarins and derivatives	90.9	Byakangelicol	C17H16O6	254.0182,239.0018	Level 2	616062
295	Authentic standards-MS-DIAL	3.71	341.0879	[M+H]+	Coumarins and derivatives	88.9	Esculin	C15H16O9	179.0332,133.0279,123.0437	Level 2	5351506
296	Authentic standards-MS-DIAL	14.12	345.1301	[M+H]+	Coumarins and derivatives	82.9	Lasepitin	C19H20O6	227.0701,83.0485, 55.0544	Level 2	511782
297	Authentic standards-MS-DIAL	16.74	351.1201	[M+Na]+	Coumarins and derivatives	87.4	Columbianadin	C19H20O5	251.0702,123.0403	Level 2	53399217

298	Authentic standards-MS-DIAL	16.56	404.1706	[M+NH4]+	Coumarins and derivatives	94.3	(9-Acetyloxy-8,8-Dimethyl-2-Oxo-9,10-Dihydropyrano[2,3-F]Chromen-10-Yl) (Z)-2-Methylbut-2-Enoate	C21H22O7	287.0898,245.0809,227.0701,175.0389	Level 2	511787
299	Authentic standards-MS-DIAL	12.51	409.1244	[M+Na]+	Coumarins and derivatives	95.6	[3-Hydroxy-2-Methyl-4-(7-Oxofuro[3,2-G]Chromen-9-Yl)Oxybutan-2-Yl] (Z)-2-Methylbut-2-Enoate	C21H22O7	309.0721,225.0142,207.1003,185.1169	Level 2	24039335
300	Authentic standards-MS-DIAL	6.03	423.1282	[M-H]-	Coumarins and derivatives	74.6	Rutarin	C20H24O10	243.0666,119.0344,89.025,59.0144	Level 2	5131188
301	Authentic standards-MS-DIAL	5.34	442.1702	[M+NH4]+	Coumarins and derivatives	73.5	2-(2-Hydroxypropan-2-Yl)-3-[(2R,3R,4S,5S,6R)-3,4,5-Trihydroxy-6-(Hydroxymethyl)Oxan-2-Yl]Oxy-2,3-Dihydrofuro[3,2-G]Chromen-7-One	C20H24O10	263.0922,187.0412,85.0278	Level 2	45359770
302	Authentic standards-MS-DIAL	20.14	449.1559	[M+Na]+	Coumarins and derivatives	96.4	[8,8-Dimethyl-9-(3-Methylbut-2-Enoyloxy)-2-Oxo-9,10-Dihydropyrano[2,3-F]Chromen-10-Yl] 3-Methylbut-2-Enoate	C24H26O7	349.1059,327.1231,251.0666,227.0697	Level 2	455809
303	Authentic standards-MS-DIAL	21.41	451.1727	[M+Na]+	Coumarins and derivatives	98.0	[8,8-Dimethyl-10-(3-Methylbut-2-Enoyloxy)-2-Oxo-9,10-Dihydropyrano[2,3-F]Chromen-9-Yl] 3-Methylbutanoate	C24H28O7	351.1211,245.0805,227.0697,175.0385, 57.07	Level 2	51136480
304	Authentic standards-MS-DIAL	22.71	453.1878	[M+Na]+	Coumarins and derivatives	95.3	[(9R,10R)-8,8-Dimethyl-9-(3-Methylbutanoyloxy)-2-Oxo-9,10-Dihydropyrano[2,3-F]Chromen-10-Yl] 3-Methylbutanoate	C24H30O7	351.1214,245.0807,175.0387,57.07	Level 2	10477794

305	Authentic standards-MS-DIAL	5.72	483.1486	[M+CH3COO]-	Coumarins and derivatives	82.5	(9R,10R)-10-Hydroxy-8,8-Dimethyl-9-[(2S,3R,4S,5S,6R)-3,4,5-Trihydroxy-6-(Hydroxymethyl)Oxan-2-Yl]Oxy-9,10-Dihydropyrano[2,3-F]Chromen-2-One	C20H24O10	423.1296,243.0667,59.0137	Level 2	23540036
306	Authentic standards-MS-DIAL	3.53	515.1365	[M-H]-	Coumarins and derivatives	80.3	Coumarin + 1O + 1Meo, O-Hex-Hex	C22H28O14	353.0864,191.0558	Level 2	73797138
307	GNPS	16.74	229.0853	[M+H]+	Coumarins and derivatives	93.7	8-Prop-1-En-2-Yl-8,9-Dihydrofuro[2,3-H]Chromen-2-One	C14H12O3	,213.0566187.045, 175.044,159.0455, 131.0512,91.057	Level 2	5318874
308	GNPS	5.86	247.0959	[M+H]+	Coumarins and derivatives	93.4	8-(2-Hydroxypropan-2-Yl)-8,9-Dihydrofuro[2,3-H]Chromen-2-One	C14H14O4	229.0861,175.0403,147.0449,91.0538	Level 2	150888
309	GNPS	6.70	296.1482	[M+NH4]+	Coumarins and derivatives	93.4	8-(2,3-Dihydroxy-3-Methylbutyl)-7-Methoxychromen-2-One	C15H18O5	261.1136,243.1029,189.0558,131.0492, 103.0541	Level 2	5070783
310	GNPS	9.59	304.1173	[M+NH4]+	Coumarins and derivatives	91.0	9-[(Z)-4-Hydroxy-3-Methylbut-2-Enoxy]Furo[3,2-G]Chromen-7-One	C16H14O5	269.08,203.0333, 173.0231,145.0277	Level 2	75069509
311	GNPS	16.74	329.1372	[M+H]+	Coumarins and derivatives	93.5	Ncgc00095690-03!(8,8-Dimethyl-2-Oxo-9,10-Dihydropyrano[2,3-H]Chromen-9-Yl) (Z)-2-Methylbut-2-Enoate	C19H20O5	229.0904,187.0437,175.0376,131.049	Level 2	511786
312	GNPS	11.61	334.1256	[M+NH4]+	Coumarins and derivatives	84.9	4-(2-Hydroxy-3-Methylbut-3-Enoxy)-9-Methoxyfuro[3,2-G]Chromen-7-One	C17H16O6	299.0924,231.0288,203.0336,175.0389	Level 2	56776189
313	GNPS	4.81	369.0806	[M-H]-	Coumarins and derivatives	83.8	7-Hydroxy-6-Methoxy-8-[(2S,3R,4S,5S,6R)-3,4,5-Trihydroxy-6-(Hydroxymethyl)Oxan-2-Yl]Oxychromen-2-One	C16H18O10	207.0294,192.0062	Level 2	5909448
314	GNPS	22.71	448.2318	[M+NH4]+	Coumarins and derivatives	92.0	[(9R,10R)-8,8-Dimethyl-9-(3-Methylbutanoyloxy)-2-Oxo-9,10-Dihydropyrano[2,3-F]Chromen-10-Yl] 3-Methylbutanoate	C24H30O7	329.1379,245.0804,227.0711,175.0385	Level 2	455807

315	GNPS	6.00	484.1805	[M+NH4]+	Coumarins and derivatives	71.7	4-[2-Hydroxy-3-Methyl-3-[(2S,3R,4S,5S,6R)-3,4,5-Trihydroxy-6-(Hydroxymethyl)Oxan-2-Yl]Oxybutoxy]Furo[3,2-G]Chromen-7-One	C22H26O11	305.1037,287.0931,203.035,85.0647	Level 2	74096942
316	RIKEN PlaSMA bio-MS/MS from plant tissues	4.85	399.0906	[M+FA-H]-	Coumarins and derivatives	78.2	6-Methoxy-7-[3,4,5-Trihydroxy-6-(Hydroxymethyl)Oxan-2-Yl]Oxychromen-2-One	C16H18O9	191.0339,176.0089	Level 2	346340
317	Fiehn/Vaniya natural product library	6.80	229.0856	[M+H]+	Coumarins and derivatives	89.6	Xanthyletin	C14H12O3	214.0608,213.0554,211.0645	Level 2	65188
318	Fiehn/Vaniya natural product library	16.50	245.1155	[M+H]+	Coumarins and derivatives	85.9	Suberosin	C15H16O3	187.0397,177.0544,69.069	Level 2	68486
319	Fiehn/Vaniya natural product library	12.68	261.1110	[M+H]+	Coumarins and derivatives	89.8	6-Methoxy-7-(3-Methylbut-2-Enoxy)Chromen-2-One	C15H16O4	193.0504,178.026, 150.0309,133.0277	Level 2	1764914
320	Fiehn/Vaniya natural product library	3.85	325.0912	[M+H]+	Coumarins and derivatives	91.2	Skimmin	C15H16O8	163.0417,119.0488,107.0489,91.0548	Level 2	320361
321	Fiehn/Vaniya natural	5.20	361.0905	[M+Na]+	Coumarins and derivatives	85.2	Gerberinside	C16H18O8	199.0373,185.0467	Level 2	14427391

	product library										
322	Fiehn/Vaniya natural product library	5.37	365.0851	[M+K]+	Coumarins and derivatives	82.4	7-[(E)-3-Methyl-4-(4-Methyl-5-Oxo-2H-Furan-2-Yl)But-2-Enoxy]Chromen-2-One	C19H18O5	203.0338,147.0437	Level 2	337783
323	GNPS-MS1	6.70	201.0552	[M+H]+	Coumarins and derivatives		3-Methylfuro[3,2-G]Chromen-7-One	C12H8O3	173.0608,115.0547,91.0538	Level 3	608273
324	GNPS-MS1	4.04	465.0632	[M-H]-	Coumarins and derivatives		2-(1,3-Benzodioxol-5-Yl)-3-(4-Hydroxy-2-Oxochromen-3-Yl)Furo[3,2-C]Chromen-4-One	C27H14O8	421.0764,216.0059,59.0138	Level 3	127252711
325	GNPS-MS1	6.87	591.1857	[M+H]+	Coumarins and derivatives		9-[2-Hydroxy-3-[3-Hydroxy-2-Methyl-4-(7-Oxofuro[3,2-G]Chromen-9-Yl)Oxybutan-2-Yl]Oxy-3-Methylbutoxy]Furo[3,2-G]Chromen-7-One	C32H30O11	287.0915,269.0804,203.0336	Level 3	85090798
326	GNPS-MS1	2.72	368.0969	[M+H]+	Coumarins and derivatives		4-Oxo-8-[3,4,5-Trihydroxy-6-(Hydroxymethyl)Oxan-2-Yl]Oxy-3H-Quinoline-2-Carboxylic Acid	C16H17NO9	206.0447,160.0387	Level 3	163100530
327	GNPS-MS1	6.33	426.1523	[M+H]+	Coumarins and derivatives		2-(7,8-Dimethoxy-4-Methyl-2-Oxochromen-3-Yl)-N-[2-(4-Methoxyphenyl)-2-Oxoethyl]Acetamide	C23H23NO7	309.0745,225.0161	Level 3	71755053
328	Fiehn/Vaniya natural product library	19.06	299.1625	[M+H]+	Coumarins and derivatives	88.9	Ostruthin	C19H22O3	243.1086,231.1017,175.0388	Level 2	5357655
329	GNPS	4.62	263.0902	[M-H]-	Coumarins and derivatives	89.8	6-(2,3-Dihydroxy-3-Methylbutyl)-7-Hydroxychromen-2-One	C14H16O5	175.0392,59.0130	Level 2	5374451

330	GNPS	5.91	423.1264	[M-H]-	Coumarins and derivatives	73.5	(2R)-9-Hydroxy-2-[2-[(2S,3R,4S,5S,6R)-3,4,5-Trihydroxy-6-(Hydroxymethyl)Oxan-2-Yl]Oxypropan-2-Yl]-2,3-Dihydrofuro[3,2-G]Chromen-7-One	C20H24O10	285.0496,227.0338	Level 2	185756
331	GNPS	16.36	573.1764	[M+H]+	Coumarins and derivatives	83.5	9-[2-[3-Hydroxy-2-Methyl-4-(7-Oxofuro[3,2-G]Chromen-9-Yl)Oxybutan-2-Yl]Oxy-3-Methylbut-3-Enoxy]Furo[3,2-G]Chromen-7-One	C32H28O10	269.0804,203.0336	Level 2	10030966
332	Authentic standards-MS-DIAL	16.94	351.2525	[M+Na]+	Glycerolipids	71.9	1,2-Dihydroxyheptadec-16-En-4-Yl Acetate	C19H36O4	259.2083,105.0684	Level 2	45360076
333	Authentic standards-MS-DIAL	21.39	370.2935	[M+NH4]+	Glycerolipids	86.4	Glyceryl Linolenate_Major	C21H36O4	353.2657,261.2206,243.2092,67.0533	Level 2	86742
334	Authentic standards-MS-DIAL	21.26	285.2426	[M-H2O+H]+	Glycerolipids	95.0	2,3-Dihydroxypropyl Tetradecanoate	C17H34O4	229.2132,95.0848,71.0838,57.0693	Level 2	79050
335	Authentic standards-MS-DIAL	28.15	359.3164	[M+H]+	Glycerolipids	84.9	2,3-Dihydroxypropyl Octadecanoate	C21H42O4	341.305,267.2678, 109.1004,57.0692	Level 2	24699
336	Authentic standards-MS-DIAL	11.86	513.3044	[M+H]+	Glycerolipids	74.6	Monogalactosylmonoacylglycerol	C27H44O9	421.2542,351.2521,259.2053,133.1	Level 2	139291989
337	Authentic standards-MS-DIAL	16.45	537.3011	[M+Na]+	Glycerolipids	85.9	2-Hydroxy-3-{[3,4,5-Trihydroxy-6-(Hydroxymethyl)Oxan-2-Yl]Oxy}Propyl (9Z,12Z,15Z)-Octadeca-9,12,15-Trienoate	C27H46O9	375.2529,259.0774,185.0425	Level 2	72984398
338	Fiehn HILIC	24.87	331.2837	[M+H]+	Glycerolipids	82.1	2-Palmitoylglycerol	C19H38O4	313.2718,95.085,71.0864,57.0701	Level 2	123409

339	RIKEN PlaSMA bio-MS/MS from plant tissues	11.79	675.3553	[M-H]-	Glycerolipids	82.9	[2-Hydroxy-3-[3,4,5-Trihydroxy-6-[[3,4,5-Trihydroxy-6-(Hydroxymethyl)Oxan-2-Yl]Oxymethyl]Oxan-2-Yl]Oxypropyl] Octadeca-9,12,15-Trienoate	C33H56O14	415.1428,397.1321,277.2159	Level 2	73034882
340	Fiehn/Vaniya natural product library	26.06	357.2972	[M+H]+	Glycerolipids	90.0	2,3-Dihydroxypropyl Octadec-9-Enoate	C21H40O4	339.2859,265.2515,247.2425,121.1008,95.0859,69.0689	Level 2	33022
341	GNPS-MS1	25.64	589.4792	[M+H]+	Glycerolipids		(3-Hydroxy-2-Tetradec-9-Enoyloxypropyl) Icosa-5,8,11-Trienoate	C37H64O5	313.2718,259.2037,119.1464	Level 3	76040717
342	GNPS-MS1	26.84	591.4963	[M+H]+	Glycerolipids		(1-Hydroxy-3-Tetradecanoyloxypropan-2-Yl) Icosa-8,11,14-Trienoate	C37H66O5	313.2714,161.2206,71.0846	Level 3	76040448
343	GNPS-MS1	10.39	647.3255	[M-H]-	Glycerolipids		[2-Hydroxy-3-[3,4,5-Trihydroxy-6-[[3,4,5-Trihydroxy-6-(Hydroxymethyl)Oxan-2-Yl]Oxymethyl]Oxan-2-Yl]Oxypropyl] Hexadeca-7,10,13-Trienoate	C31H52O14	397.1343,249.1833	Level 3	162823028
344	GNPS	7.84	531.2797	[M+HCOO]-	Glycerolipids	73.2	[1-Hydroxy-3-[3,4,5-Trihydroxy-6-(Hydroxymethyl)Oxan-2-Yl]Oxypropan-2-Yl] Hexadeca-7,10,13-Trienoate	C25H42O9	285.2796,250.1738	Level 2	145994451
345	GNPS	24.53	613.4753	[M+H]+	Glycerolipids	70.8	[(2S)-3-Hydroxy-2-[(6Z,9Z,12Z,15Z)-Octadeca-6,9,12,15-Tetraenoyl]Oxypropyl] (9Z,12Z)-Octadeca-9,12-Dienoate	C39H64O5	475.2592,335.2544	Level 2	53478106
346	Authentic standards-MS-DIAL	0.72	258.1108	[M]+	Glycerophospholipids	96.5	Sn-Glycero-3-Phosphocholine	[C8H21NO6P]+	184.0735,125.0005,104.1074,86.0975	Level 2	823

347	Authentic standards-MS-DIAL	17.21	454.2909	[M+H]+	Glycerophospholipids	90.4	1-Palmitoyl-2-Hydroxy-Sn-Glycero-3-Phosphoethanolamine	C21H44NO7P	436.283,393.2392, 313.2736,282.2779	Level 2	9547069
348	Authentic standards-MS-DIAL	16.31	478.2913	[M+H]+	Glycerophospholipids	83.1	Lpe 18:2	C23H44NO7P	460.2825,337.2727,95.085	Level 2	75958059
349	Authentic standards-MS-DIAL	13.55	478.2918	[M-H]-	Glycerophospholipids	83.3	Lpe 18:1	C23H46NO7P	281.2467,195.7811	Level 2	74413339
350	Authentic standards-MS-DIAL	15.94	482.3232	[M+H]+	Glycerophospholipids	89.3	1-Pentadecanoyl-2-Hydroxy-Sn-Glycero-3-Phosphocholine	C23H48NO7P	464.3114,184.0732,104.1073	Level 2	24779458
351	Authentic standards-MS-DIAL	17.40	496.3386	[M+H]+	Glycerophospholipids	92.0	Lpc 16:0	C24H50NO7P	313.2735,184.0746,104.1073	Level 2	86554
352	Authentic standards-MS-DIAL	16.48	520.3386	[M+H]+	Glycerophospholipids	91.4	Lpc 18:2	C26H50NO7P	184.0733,104.1073	Level 2	25200694
353	Authentic standards-MS-DIAL	18.16	522.3525	[M+H]+	Glycerophospholipids	88.7	Lpc 18:1	C26H52NO7P	339.295,184.0734, 104.1074	Level 2	3082003
354	Authentic standards-MS-DIAL	20.47	524.3693	[M+H]+	Glycerophospholipids	96.4	Lysopc(0:0/18:0)	C26H54NO7P	341.3037,184.0719,104.1072	Level 2	24779491
355	Authentic standards-MS-DIAL	17.60	673.4753	[M-H]-	Glycerophospholipids	83.8	[1-Hexadecanoyloxy-3-Phosphonooxypropan-2-Yl]Octadec-9-Enoate	C37H71O8P	391.2207,281.2463,255.2327,152.9966	Level 2	656793

356	Authentic standards-MS-DIAL	18.05	714.5016	[M-H]-	Glycerophospholipids	77.7	Pe 34:2	C39H74NO8P	279.2291,255.2315	Level 2	6426727
357	Authentic standards-MS-DIAL	18.20	717.4619	[M-H]-	Glycerophospholipids	73.0	Pg 32:2	C38H71O10P	481.2559,253.2139	Level 2	11607301
358	Authentic standards-MS-DIAL	18.46	719.4789	[M-H]-	Glycerophospholipids	84.0	Pg 32:1	C38H73O10P	483.2686,255.2328	Level 2	138220866
359	Authentic standards-MS-DIAL	18.80	721.4967	[M-H]-	Glycerophospholipids	87.2	Pg 32:0	C38H75O10P	391.2224,255.2326	Level 2	65144
360	Authentic standards-MS-DIAL	17.57	738.5024	[M-H]-	Glycerophospholipids	84.3	Pe 36:4	C41H74NO8P	458.2639,279.2322	Level 2	188345
361	Authentic standards-MS-DIAL	18.34	743.4815	[M-H]-	Glycerophospholipids	82.6	Pg 34:3	C40H73O10P	279.2436,253.2205	Level 2	138239342
362	Authentic standards-MS-DIAL	18.84	745.4975	[M-H]-	Glycerophospholipids	88.9	Pg 34:2	C40H75O10P	279.2381,255.2386152.9963	Level 2	138121875
363	Authentic standards-MS-DIAL	18.17	769.4969	[M-H]-	Glycerophospholipids	89.2	Pg 36:4	C42H75O10P	507.2652,279.232	Level 2	49792064
364	Authentic standards-MS-DIAL	18.13	791.4893	[M-H]-	Glycerophospholipids	83.9	Pg 38:7	C44H73O10P	529.2947,279.23	Level 2	138147428

365	Authentic standards-MS-DIAL	17.85	807.4908	[M-H]-	Glycerophospholipids	72.8	Pi 32:1	C41H77O13P	553.3762,241.0077	Level 2	138241787
366	Authentic standards-MS-DIAL	18.61	809.5097	[M-H]-	Glycerophospholipids	82.5	Pi 32:0	C41H79O13P	553.2794,391.2207,255.2312	Level 2	14185941
367	Authentic standards-MS-DIAL	15.91	847.4920	[M-H]-	Glycerophospholipids	86.1	Pi(16:1/13-Hode)	C43H77O14P	293.2131,241.011	Level 2	134777058
368	Authentic standards-MS-DIAL	17.18	855.4955	[M-H]-	Glycerophospholipids	83.5	Pi 36:5	C45H77O13P	241.0112	Level 2	138154651
369	Authentic standards-MS-DIAL	17.64	857.5109	[M-H]-	Glycerophospholipids	84.6	Pi 36:4	C45H79O13P	241.0125,152.9955	Level 2	138284476
370	Authentic standards-MS-DIAL	18.12	859.5286	[M-H]-	Glycerophospholipids	91.1	Pi 36:3	C45H81O13P	579.2913,417.2385,241.0107	Level 2	138215541
371	Authentic standards-MS-DIAL	17.21	877.5322	[M-H]-	Glycerophospholipids	71.9	Pi(18:0/9-Hode)	C45H83O14P	603.3741,295.225	Level 2	134738657
372	RIKEN PlaSMA bio-MS/MS from plant tissues	13.14	452.2756	[M-H]-	Glycerophospholipids	89.9	Lpe 16:0	C21H44NO7P	255.2334,214.0469,78.9588	Level 2	89229
373	RIKEN PlaSMA bio-	11.58	518.3212	[M+H]+	Glycerophospholipids	71.9	Lpc 18:3	C26H48NO7P	184.072,104.1063, 86.0947	Level 2	25200367

	MS/MS from plant tissues										
374	GNPS-MS1	16.32	500.2717	[M+H]+	Glycerophospholipids		[1-[2-Aminoethoxy(Hydroxy)Phosphoryl]Oxy-3-Hydroxypropan-2-Yl] Icosa-5,8,11,14,17-Pentaenoate	C25H42NO7P	457.2321,337.2724,195.0036	Level 3	76043315
375	GNPS-MS1	12.74	504.3057	[M-H]-	Glycerophospholipids		[3-[2-Aminoethoxy(Hydroxy)Phosphoryl]Oxy-2-Hydroxypropyl] Icosa-11,14-Dienoate	C25H48NO7P	279.2308,123.1569	Level 3	75958068
376	GNPS-MS1	28.55	712.4860	[M+H]+	Glycerophospholipids		[3-[2-Aminoethoxy(Hydroxy)Phosphoryl]Oxy-2-Tetradec-9-Enoyloxypropyl] Icosa-8,11,14-Trienoate	C39H70NO8P	571.4690,315.2292,97.0646	Level 3	75957593
377	GNPS	11.39	571.2849	[M-H]-	Glycerophospholipids	94.6	[(2R)-2-Hydroxy-3-[Hydroxy-[(2R,3R,5S,6R)-2,3,4,5,6-Pentahydroxycyclohexyl]Oxyphosphoryl] Oxypropyl] Hexadecanoate	C25H49O12P	255.2320,241.0116,152.9959	Level 2	71296207
378	GNPS	17.86	833.5198	[M-H]-	Glycerophospholipids	95.7	[1-Hexadecanoyloxy-3-[Hydroxy-(2,3,4,5,6-Pentahydroxycyclohexyl)Oxyphosphoryl] Oxypropan-2-Yl] (9Z,12Z)-Octadeca-9,12-Dienoate	C43H79O13P	279.2381,255.2385	Level 2	138130059
379	GNPS	23.28	835.5304	[M-H]-	Glycerophospholipids	74.2	[1-Hexadecanoyloxy-3-[Hydroxy-(2,3,4,5,6-Pentahydroxycyclohexyl)Oxyphosphoryl] Oxypropan-2-Yl] (Z)-Octadec-9-Enoate	C43H81O13P	553.2791,255.2312	Level 2	138234040

380	Authentic standards-MS-DIAL	8.85	357.1318	[M-H]-	Lignans and derivatives	85.4	Matairesinol	C20H22O6	342.1096,137.0592,211.0826,83.0131	Level 2	345761
381	Authentic standards-MS-DIAL	8.31	357.1321	[M-H]-	Lignans and derivatives	86.3	Pinoresinol	C20H22O6	342.1063,151.0398,136.0161	Level 2	234817
382	Authentic standards-MS-DIAL	7.17	361.1636	[M-H]-	Lignans and derivatives	87.4	Secoisolariciresinol	C20H26O6	346.1379,165.0553,122.0358	Level 2	586372
383	Authentic standards-MS-DIAL	6.56	579.2047	[M-H]-	Lignans and derivatives	93.3	Acanthoside B	C28H36O13	417.154,402.1314,181.0507,166.0261	Level 2	4486984
384	GNPS	5.58	357.1309	[M-H]-	Lignans and derivatives	94.0	(3R,4R)-3,4-Bis[(4-Hydroxy-3-Methoxyphenyl)Methyl]Oxolan-2-One	C20H22O6	135.0311,92.0258	Level 2	119205
385	GNPS	8.09	417.1489	[M-H]-	Lignans and derivatives	71.6	4-[(3Ar,6S,6Ar)-6-(4-Hydroxy-3,5-Dimethoxyphenyl)-1,3,3A,4,6,6A-Hexahydrofuro[3,4-C]Furan-3-Yl]-2,6-Dimethoxyphenol	C22H26O8	387.1086,181.0507,59.0137	Level 2	332426
386	GNPS	7.02	519.1844	[M-H]-	Lignans and derivatives	88.9	(3R,4R)-4-[(4-Hydroxy-3-Methoxyphenyl)Methyl]-3-[[3-Methoxy-4-[(2S,3R,4S,5S,6R)-3,4,5-Trihydroxy-6-(Hydroxymethyl)Oxan-2-Yl]Oxyphenyl]Methyl]Oxolan-2-One	C26H32O11	357.1334,83.0132	Level 2	486612
387	GNPS	6.13	521.1979	[M-H]-	Lignans and derivatives	81.8	(2S,3R,4R,5S,6R)-2-[4-[(2S,3R,4R)-4-[(4-Hydroxy-3-Methoxyphenyl)Methyl]-3-(Hydroxymethyl)Oxolan-2-Yl]-2-Methoxyphenoxy]-6-(Hydroxymethyl)Oxane-3,4,5-Triol	C26H34O11	295.1688,179.0574	Level 2	163074664

388	Authentic standards-MS-DIAL	22.63	293.2455	[M+H]+	Steroids and steroid derivatives	72.4	Androstane-3,17-Diol	C19H32O2	275.2005,109.1005,95.0839,81.0695	Level 2	11301
389	GNPS	33.80	425.3746	[M-H2O+H]+	Steroids and steroid derivatives	75.0	15-(5-Hydroxy-6-Methylhept-6-En-2-Yl)-7,7,12,16-Tetramethylpentacyclo[9.7.0.01,3.03,8.012,16]Octadecan-6-Ol	C30H50O2	407.3652,177.1634,95.0837	Level 2	14313590
390	Fiehn HILIC	12.72	275.2004	[M+H]+	Steroids and steroid derivatives	81.7	Nandrolone	C18H26O2	257.1895,199.1463,173.1328,119.085	Level 2	220503
391	RIKEN PlaSMA bio-MS/MS from plant tissues	22.13	387.2529	[M+H]+	Steroids and steroid derivatives	73.8	Bufalin	C24H34O4	369.2379,351.2333	Level 2	2464
392	Fiehn/Vaniya natural product library	15.94	315.2307	[M+H]+	Steroids and steroid derivatives	84.9	Progesterone	C21H30O2	297.2172,109.0637,97.0648	Level 2	4920
393	GNPS	12.74	339.1982	[M-H]-	Steroids and steroid derivatives	91.7	(9S,14S)-10,13-Dimethylspiro[2,8,9,11,12,14,15,16-Octahydro-1H-Cyclopenta[A]Phenanthrene-17,5'-Oxolane]-2',3-Dione	C22H28O3	183.0110,170.0039	Level 2	24728626
394	GNPS	13.71	249.1845	[M-H2O+H]+	Terpenes and derivatives	71.2	3-Hydroxy-1-[(1R,2R,4Ar,6S,8As)-1,2,4A,5,6,7,8,8A-Octahydro-2-Hydroxy-1,2,6-Trimethyl-1-Naphthalenyl]-1-Propanone	C16H26O3	213.1638,119.0849,93.0697,79.0544	Level 2	73837707

395	Fiehn/Vaniya natural product library	5.75	251.1641	[M+H]+	Terpenes and derivatives	75.6	8-Hydroxy-11-(Hydroxymethyl)-1,5,11-Trimethyltricyclo[6.2.1.0?,?]Undec-2-En-9-One	C15H22O3	233.1535,147.1161,91.0528	Level 2	57509414
396	GNPS	15.03	415.2113	[M-H2O+H]+	Terpenes and derivatives	82.3	(12-Acetyloxy-5,11,15,15-Tetramethyl-6-Oxo-2,7-Dioxapentacyclo[8.8.0.01,3.04,8.011,16]Octadec-4-En-14-Yl) Acetate	C24H32O7	119.085,91.0537	Level 2	138376535
397	Authentic standards-MS-DIAL	20.76	205.1949	[M-H2O+H]+	Terpenes and derivatives	87.8	3,7,11-Trimethyldodeca-2,6,10-Trien-1-Ol	C15H26O	149.1317,121.1019,93.0697,81.0695,55.0543	Level 2	3327
398	Authentic standards-MS-DIAL	11.75	219.1734	[M-2H2O+H]+	Terpenes and derivatives	87.5	5-Hydroxyculmorin	C15H26O3	201.1638,175.1503,145.101,105.0694	Level 2	122706895
399	Authentic standards-MS-DIAL	17.19	219.1735	[M+H]+	Terpenes and derivatives	79.7	Nootkatone	C15H22O	201.1641,131.0851,67.0527	Level 2	1268142
400	Authentic standards-MS-DIAL	6.41	247.1311	[M-H]-	Terpenes and derivatives	84.5	(3Ar,5Ar,9Br)-3A-Hydroxy-5A,9-Dimethyl-3-Methylidene-4,5,6,7,8,9B-Hexahydrobenzo[G][1]Benzofuran-2-One	C15H20O3	203.1423,163.1112,149.0966	Level 2	13995523
401	Authentic standards-MS-DIAL	20.87	273.2560	[M-H2O+H]+	Terpenes and derivatives	78.4	(Z)-5-(5,5,8A-Trimethyl-2-Methylidene-3,4,4A,6,7,8-Hexahydro-1H-Naphthalen-1-Yl)-3-Methylpent-2-En-1-Ol	C20H34O	203.1786,81.0704	Level 2	75528899
402	Authentic standards-MS-DIAL	4.38	391.1959	[M+CH3COO]-	Terpenes and derivatives	80.9	2-(Hydroxymethyl)-6-(6-Hydroxy-6-Methyl-3-Propan-2-Ylcyclohex-3-En-1-Yl)Oxyoxane-3,4,5-Triol	C16H28O7	101.0251,71.0135, 59.0137	Level 2	56776223

403	Authentic standards-MS-DIAL	14.45	415.2119	[M+Na]+	Terpenes and derivatives	84.4	(1S,2R,4Ar,8Ar)-1-Acetoxy-7-Isopropylidene-1,4A-Dimethyl-6-Oxodecahydro-2-Naphthalenyl 2,3-Dimethyl-2-Oxiranecarboxylate	C22H32O6	119.085,91.0546	Level 2	14414238
404	Authentic standards-MS-DIAL	20.03	425.3774	[M+H]+	Terpenes and derivatives	87.7	17(21)-Hopen-6-One	C30H48O	407.3641,217.1934,109.1005	Level 2	138756179
405	Authentic standards-MS-DIAL	27.14	441.3702	[M+H]+	Terpenes and derivatives	82.9	22-Hydoxy-2-Hopen-1-One	C30H48O2	423.3603,191.1796,135.1144	Level 2	138756182
406	Authentic standards-MS-DIAL	30.08	551.4221	[M+H]+	Terpenes and derivatives	80.0	Celaxanthin	C40H54O	345.2563,175.1488	Level 2	89049130
407	Authentic standards-MS-DIAL	24.40	565.4008	[M+H]+	Terpenes and derivatives	75.5	3-Hydroxy-4-Ketotorulene	C40H52O2	473.338,221.1528	Level 2	87443980
408	Authentic standards-MS-DIAL	30.07	567.4159	[M+H]+	Terpenes and derivatives	81.9	3-Hydroxy-4-Keto-Gamma-Carotene	C40H54O2	549.4084,133.1009	Level 2	87398669
409	Authentic standards-MS-DIAL	31.49	567.4160	[M+H]+	Terpenes and derivatives	77.7	3-Hydroxyechinenone	C40H54O2	549.4016,145.1008	Level 2	10120578
410	Authentic standards-MS-DIAL	24.86	583.4119	[M+H]+	Terpenes and derivatives	79.3	Diadinochrome	C40H54O3	565.402,283.2056, 221.1516	Level 2	101176786
411	Authentic standards-MS-DIAL	30.07	585.4273	[M+H]+	Terpenes and derivatives	78.0	Antheraxanthin	C40H56O3	567.4188,167.1057	Level 2	5281223

412	Authentic standards-MS-DIAL	11.00	943.5247	[M+H]+	Terpenes and derivatives	77.5	Soyasaponin Bb	C48H78O18	797.4589,599.395,441.3724,141.016	Level 2	122097
413	MassBank	30.08	568.4225	[M]+	Terpenes and derivatives	80.1	Tunaxanthin	C40H56O2	550.4131,476.3644,145.1009	Level 2	6384258
414	GNPS	7.82	219.1735	[M-H2O+H]+	Terpenes and derivatives	82.1	6-Hydroxy-3,8-Dimethyl-5-Propan-2-Yl-4A,5,6,7,8,8A-Hexahydro-1H-Naphthalen-2-One	C15H24O2	219.1739,119.085	Level 2	74494816
415	GNPS	8.63	231.1385	[M-H2O+H]+	Terpenes and derivatives	80.5	(9E)-3,6,10-Trimethyl-7,8,11,11A-Tetrahydrocyclodeca[B]Furan-2,5(4H,6H)-Dione	C15H20O3	213.1253,185.1252145.1002,128.0617	Level 2	74975743
416	GNPS	11.54	253.1803	[M+H]+	Terpenes and derivatives	90.7	2-(8-Hydroxy-4A,8-Dimethyl-1,2,3,4,5,6,7,8A-Octahydronaphthalen-2-Yl)Prop-2-Enoic Acid	C15H24O3	189.0342,163,0569	Level 2	496073
417	GNPS	3.89	265.1433	[M-H2O+H]+	Terpenes and derivatives	77.4	3-(8-Hydroxy-6,8-Dimethyl-3-Methylidene-2-Oxo-3A,4,5,6,7,8A-Hexahydrocyclohepta[B]Furan-7-Yl)Propanoic Acid	C15H22O5	201.1268175.0769, 119.0853,91.054	Level 2	75110934
418	GNPS	26.39	307.2614	[M-H2O+H]+	Terpenes and derivatives	75.0	5-(2-Hydroxy-2,5,5,8A-Tetramethyl-3,4,4A,6,7,8-Hexahydro-1H-Naphthalen-1-Yl)-3-Methylpentanoic Acid	C20H36O3	137.1314,123.1162,109.1005	Level 2	3430304
419	GNPS	30.89	351.2668	[M-H2O+H]+	Terpenes and derivatives	80.4	8-(Hydroxymethyl)-1,4-Dimethyl-12-(6-Methylhepta-3,5-Dien-2-Yl)Tricyclo[9.3.0.03,7]Tetradeca-4,8-Dien-6-One	C25H36O2	295.1996,157.1017,105.0683	Level 2	76022791
420	GNPS	4.71	385.1468	[M+Na]+	Terpenes and derivatives	85.6	3-Hydroxy-2-[3-Methyl-2-[[3,4,5-Trihydroxy-6-(Hydroxymethyl)Oxan-2-	C16H26O9	205.1185,161.0207	Level 2	51136579

							Yl]Oxymethyl]Cyclopent-2-En-1-Yl]Propanoic Acid				
421	GNPS	31.39	409.3822	[M-H2O+H]+	Terpenes and derivatives	76.6	2-(2-Hydroxy-2-(3-(Trifluoromethyl)Phenyl)-4-Morpholinyl)Ethyl Acetate	C30H50O	123.1173,95.0849, 81.0695	Level 2	225689
422	GNPS	24.04	439.3562	[M-H2O+H]+	Terpenes and derivatives	83.3	Ursolic Acid	C30H48O3	393.3515,247.1671,191.1797,149.1305	Level 2	220774
423	Fiehn/Vaniya natural product library	7.44	219.1724	[M+H]+	Terpenes and derivatives	72.9	Muurolladie-3-One	C15H22O	147.1163,107.0854	Level 2	3989419
424	Fiehn/Vaniya natural product library	5.76	265.1411	[M-H]-	Terpenes and derivatives	73.9	(4Ar,5S)-9,9A-Dihydroxy-3,4A,5-Trimethyl-5,6,7,8,8A,9-Hexahydro-4H-Benzo[F][1]Benzofuran-2-One	C15H22O4	247.1324,221.1532,203.1426	Level 2	75110996
425	Fiehn/Vaniya natural product library	19.53	305.2463	[M+H]+	Terpenes and derivatives	76.0	(4S,4Ar,8As)-4-[(3R)-3-Hydroxy-3-Methylpent-4-Enyl]-3,4A,8,8-Tetramethyl-5,6,7,8A-Tetrahydro-4H-Naphthalen-1-One	C20H32O2	121.0997,79.0544	Level 2	24721562
426	Fiehn/Vaniya natural product library	3.73	359.1336	[M-H]-	Terpenes and derivatives	75.0	7-Methyl-1-[(2S,3R,4S,5S,6R)-3,4,5-Trihydroxy-6-(Hydroxymethyl)Oxan-2-Yl]Oxy-1,4A,5,6,7,7A-Hexahydrocyclopenta[C]Pyran-4-Carboxylic Acid	C16H24O9	197.0809,153.0919,59.0138	Level 2	4490284

427	GNPS-MS1	2.91	385.1121	[M-H]-	Terpenes and derivatives	Methyl 7-Formyl-1-[3,4,5-Trihydroxy-6-(Hydroxymethyl)Oxan-2-Yl]Oxy-1,4A,5,7A-Tetrahydrocyclopenta[C]Pyran-4-Carboxylate	C17H22O10	223.0608,208.0365,164.0478	Level 3	14635618
428	GNPS-MS1	26.91	397.3811	[M+H]+	Terpenes and derivatives	4,6A,6B,8A,11,11,14B-Heptamethyl-2,3,4,4A,5,6,7,8,9,10,12,12A,14,14A-Tetradecahydro-1H-Picene	C29H48	227.125	Level 3	85447087
429	GNPS-MS1	5.85	403.1331	[M-H]-	Terpenes and derivatives	Methyl 7-(Furan-3-Yl)-15-Hydroxy-9-Methyl-5,14-Dioxo-6,13-Dioxatetracyclo[10.2.2.01,10.04,9]Hexadecane-15-Carboxylate	C21H24O8	179.0707,59.0138	Level 3	163008097
430	GNPS-MS1	25.41	413.3375	[M+H]+	Terpenes and derivatives	8-Hydroxy-3A,5B-Dimethyl-3-(6-Methyl-5-Methylideneheptan-2-Yl)-1,2,3,4,5,5A,6,7,8,9,10A,10B-Dodecahydrocyclopenta[A]Fluorene-10-Carbaldehyde	C28H44O2	105.0673	Level 3	56680719
431	GNPS-MS1	28.16	413.3764	[M+H]+	Terpenes and derivatives	4,4,6A,6B,11,11,14B-Heptamethyl-2,3,4A,5,6,7,8,8A,9,10,12,12A,14,14A-Tetradecahydro-1H-Picen-3-Ol	C29H48O	225.1655,117.0699	Level 3	78020094
432	GNPS-MS1	26.29	415.3522	[M+H]+	Terpenes and derivatives	4,4,6B,8A,11,11-Hexamethyl-1,2,3,4A,5,6,6A,7,8,9,10,12,12A,14,14A,14B-Hexadecahydropicene-3,8-Diol	C28H46O2	283.2383,137.1325,95.0849	Level 3	163067314
433	GNPS-MS1	26.91	423.3558	[M+H]+	Terpenes and derivatives	4,4,6A,6B,8A,11,11,14B-Octamethyl-2,4A,5,6,7,8,9,10,12,12A-Decahydro-1H-Picen-3-One	C30H46O	303.2248,165.012	Level 3	13918533
434	GNPS-MS1	25.21	427.3558	[M+H]+	Terpenes and derivatives	3A,6,10A,12A-Tetramethyl-1-(6-Methylheptan-2-Yl)-2,3,5,5A,6,9,10,12-	C29H46O2	191.1785,69.0324	Level 3	78292126

							Octahydro-1H-Indeno[5,4-G][2]Benzoxepin-8-One				
435	GNPS-MS1	27.97	427.3938	[M+H]+	Terpenes and derivatives		4,4A,6A,6B,8A,11,11,14A-Octamethyl-2,4,5,6,6A,7,8,9,10,12,12A,13,14,14B-Tetradecahydro-1H-Picen-3-One	C30H50O	409.3802,191.1783,81.0695	Level 3	163096703
436	GNPS-MS1	26.26	429.3702	[M+H]+	Terpenes and derivatives		2-Hydroxy-4,4A,6B,8A,11,11,14A-Heptamethyl-1,2,4,5,6,6A,6A,7,8,9,10,12,12A,13,14,14B-Hexadecahydropicen-3-One	C29H48O2	411.3615,191.1783,67.0533	Level 3	163087411
437	GNPS-MS1	24.42	567.3273	[M+H]+	Terpenes and derivatives		[3-[1-Hydroxy-2-[2-Hydroxy-2-(Hydroxymethyl)-4B,8,8,10A-Tetramethyl-1,3,4,4A,5,6,7,8A,9,10-Decahydrophenanthren-1-Yl]Ethyl]-5-Oxo-2H-Furan-2-Yl] 3-Phenylprop-2-Enoate	C34H46O7	221.15828,67.0533	Level 3	85228224
438	GNPS-MS1	22.61	605.3054	[M+H]+	Terpenes and derivatives		5,7-Dihydroxy-2-[8-Hydroxy-4-[(3-Hydroxy-5,5,8A-Trimethyl-2-Methylidene-3,4,4A,6,7,8-Hexahydro-1H-Naphthalen-1-Yl)Methyl]-7-Methoxy-3-Methyl-5-Oxo-1,4,8,8A-Tetrahydronaphthalen-4A-Yl]Chromen-4-One	C36H44O8	403.2817,225.0143	Level 3	75068915
439	Authentic standards-MS-DIAL	23.97	259.2044	[M+H]+	Terpenes and derivatives	74.2	6-Acetyl-1,1,2,4,4,7-Hexamethyltetralin	C18H26O	175.1478,119.085	Level 2	89440
440	GNPS	8.40	203.1799	[M-H2O+H]+	Terpenes and derivatives	82.6	1-Methyl-4-Methylidene-7-Propan-2-Yl-2,3,3A,5,6,8A-Hexahydroazulen-1-Ol	C15H24O	175.1467,147.1175,105.0696	Level 2	73102746

441	Authentic standards-MS-DIAL	3.52	298.0960	[M+H]+	Others	90.4	5'-S-Methylthioadenosine	C11H15N5O3S	136.063,119.0359	Level 2	439176
442	Authentic standards-MS-DIAL	4.28	225.1496	[M+H]+	Others	79.8	2-(6-Methyl-7-Oxooctyl)-2H-Furan-5-One	C13H20O3	189.1299,151.1109,119.0841,77.0382	Level 2	11075054
443	Authentic standards-MS-DIAL	9.26	213.1478	[M-H2O+H]+	Others	75.3	5-(Hydroxymethyl)-3-(1-Hydroxy-5-Methylhexyl)Oxolan-2-One	C12H22O4	149.133,107.0852, 81.0696	Level 2	57509302
444	Authentic standards-MS-DIAL	24.67	282.2784	[M+H]+	Others	74.9	Oleamide	C18H35NO	247.2426,135.1167,83.085	Level 2	1930
445	Fiehn HILIC	30.43	338.3412	[M+H]+	Others	96.6	Erucamide	C22H43NO	321.3158,303.3069,135.1167,83.0859, 69.0714	Level 2	8214
446	Authentic standards-MS-DIAL	20.14	327.1220	[M-H2O+H]+	Others	90.5	11-Methyl-2,7-Dimethylidene-6,12-Dioxo-5,14-Dioxatricyclo[9.2.1.0?,?]Tetradec-1(13)-En-9-Yl 2-Methylprop-2-Enoate	C19H20O6	227.0699,175.0375,83.0493,55.0544	Level 2	14807501
447	GNPS	8.69	263.1626	[M-H2O+H]+	Others	75.6	4-Hydroxy-1-Methoxy-6,6,9A-Trimethyl-4,5,5A,6,7,8,9,9A-Octahydronaphtho[1,2-C]Furan-3(1H)-One	C16H24O4	245.1514,135.0792,91.0546	Level 2	45783042
448	Authentic standards-MS-DIAL	10.79	230.2470	[M+H]+	Others	89.5	N,N-Dimethyldodecylamine N-Oxide	C14H31NO	212.237,71.0856,57.0693	Level 2	15433
449	Authentic standards-MS-DIAL	10.70	274.2730	[M+H]+	Others	97.5	Lauryldiethanolamine	C16H35NO2	256.2633,230.2476,88.0757,70.0647	Level 2	352309

450	Authentic standards-MS-DIAL	13.21	302.3048	[M+H]+	Others	93.9	2,2'-(Tetradecylimino)Diethanol	C18H39NO2	284.295,106.0855, 88.0755	Level 2	12942137
451	Authentic standards-MS-DIAL	12.51	316.2836	[M+H]+	Others	85.5	Dehydrophytosphingosine	C18H37NO3	298.2758,280.2642,60.0441	Level 2	25203815
452	Authentic standards-MS-DIAL	13.37	318.2987	[M+H]+	Others	94.3	Phytosphingosine	C18H39NO3	300.2886,282.278, 60.0442	Level 2	248575
453	Authentic standards-MS-DIAL	23.34	326.3035	[M+H]+	Others	82.1	Oleoyl Ethanolamide	C20H39NO2	309.2738,62.0595	Level 2	5283454
454	Authentic standards-MS-DIAL	18.66	233.1319	[M-2H2O+H]+	Others	81.5	3,6-Diphenylcyclohexane-1,2-Diol	C18H20O2	191.0877,117.0691,91.0537	Level 2	14685115
455	GNPS-MS1	6.13	328.1174	[M+H]+	Others		3-(3-Acetylanilino)-6,7-Dimethoxy-3H-2-Benzofuran-1-One	C18H17NO5	269.0803,203.0335,84.0796	Level 3	3394829
456	GNPS-MS1	8.72	329.0999	[M+H]+	Others		3-[2-(4-Hydroxyphenyl)-2-Oxoethyl]-6,7-Dimethoxy-3H-2-Benzofuran-1-One	C18H16O6	269.0804,201.0179,173.0232	Level 3	2863812
457	GNPS-MS1	7.94	331.0800	[M+H]+	Others		2-[(4,6-Dihydroxy-7-Methyl-3-Oxo-1H-2-Benzofuran-5-Yl)Oxy]-6-Hydroxy-4-Methylbenzaldehyde	C17H14O7	204.0347,81.0688	Level 3	76764468
458	GNPS-MS1	6.76	403.1015	[M+H]+	Others		17-Hydroxy-4-(Hydroxymethyl)-5,13-Dimethoxy-7,12-Dimethyl-2,10,16-Trioxatetracyclo[9.7.0.03,8.014,18]Octadeca-1(18),3(8),4,6,11,13-Hexaene-9,15-Dione	C20H18O9	269.0804,201.0179,67.0534	Level 3	162876621

459	Authentic standards-MS-DIAL	20.16	279.1585	[M+H]+	Others	92.8	Di-N-Butyl Phthalate	C16H22O4	149.031,121.0306, 93.0337,65.0383	Level 2	3026
460	Authentic standards-MS-DIAL	22.11	339.1567	[M+H]+	Others	83.6	Bergamottin	C21H22O4	203.0333,147.0446	Level 2	119057
461	Authentic standards-MS-DIAL	21.21	339.1583	[M+H]+	Others	90.0	9-{[(2E)-3,7-Dimethyl-2,6-Octadienyl]Oxy}-7H-Furo[3,2-G]Chromen-7-One	C21H22O4	203.0333,175.0399,137.1337,95.0849	Level 2	155658
462	Authentic standards-MS-DIAL	14.99	377.1353	[M+Na]+	Others	79.2	Notopterol	C21H22O5	325.0145,176.1141	Level 2	3086274
463	GNPS-MS1	7.33	313.1059	[M+H]+	Others		6,7-Dihydroxy-1,6-Dimethyl-8,9-Dihydro-7H-Naphtho[1,2-G][1]Benzofuran-10,11-Dione	C18H16O5	177.0546,145.0292,117.0332,89.0386	Level 3	14376773
464	GNPS-MS1	5.72	406.1478	[M+H]+	Others		4-Hydroxy-6-Methyl-3-[2-(4-Propan-2-Ylphenyl)-2,3-Dihydro-1,5-Benzothiazepin-4-Yl]Pyran-2-One	C24H23NO3S	269.0806,201.0181,74.0602	Level 3	135400391
465	GNPS-MS1	7.15	452.1707	[M+H]+	Others		4-[1-[4-Hydroxy-1-[2-(4-Hydroxyphenyl)Ethyl]-6-Methyl-2-Oxopyridin-3-Yl]-3-Methoxy-3-Oxopropyl]Benzoic Acid	C25H25NO7	452.1717,269.0807,120.0808	Level 3	102564695
466	GNPS-MS1	20.87	475.2825	[M+H]+	Others		4'-Ethenyl-4',11-Dimethyl-8-(6-Methylhept-5-En-2-Yl)-2-Phenylspiro[3-Oxatricyclo[5.4.0.02,6]Undec-10-Ene-4,3'-Oxolane]-2',5-Dione	C31H38O4	273.2585,203.0334	Level 3	72980936
467	GNPS-MS1	4.96	535.2335	[M-H]-	Others		(1-Acetyloxy-8-Benzoyloxy-3-Hydroxy-6,8A-Dimethyl-3-Propan-2-Yl-	C31H36O8	475.1836,331.1741,59.0137	Level 3	73000259

							1,2,3A,4,5,8-Hexahydroazulen-4-Yl) 4-Hydroxybenzoate				
468	GNPS-MS1	22.61	547.3033	[M+H]+	Others		3-(3,7-Dimethylocta-2,6-Dienyl)-2-[6-Hydroxy-8-Methoxy-2-Methyl-2-(4-Methylpent-3-Enyl)Chromen-5-Yl]-5-Methoxycyclohexa-2,5-Diene-1,4-Dione	C34H42O6	269.0798,109.0994	Level 3	76387853
469	MassBank	6.80	336.1227	[M]+	Others	90.2	Berberine	C20H18NO4	306.077,292.095, 278.0818	Level 2	2353
470	Authentic standards-MS-DIAL	19.42	286.2147	[M+H]+	Others	82.1	1-Methyl-2-Nonylquinolin-4-One	C19H27NO	172.1105,130.0647	Level 2	13967189
471	GNPS	11.37	311.1683	[M-H]-	Others	84.0	(3E,5S,8R,11E,13S,16R)-5,13-Dihydroxy-8,16-Dimethyl-1,9-Dioxacyclohexadeca-3,11-Diene-2,10-Dione	C16H24O6	119.0483,91.0548	Level 2	10935870
472	GNPS	12.07	325.1830	[M-H]-	Others	94.0	(5-Ethyl-1-Azabicyclo[2.2.2]Octan-2-Yl)-(6-Methoxyquinolin-4-Yl)Methanol	C20H26N2O2	197.0202,184.0122	Level 2	3649

[a] represents Compound were verified by the standard, *CID represents Compound CID from Pubchem .

Table S3 Detailed information on 94 active ingredients screened by TCMSP and Swiss ADME

No	Database	t_R/min	m/z	Adduct type	Class	Total score	Name	Formula	MS/MS Fragment Ions	MSI Level	CID
C1	Authentic standards-MS-DIAL	21.41	451.1727	[M+Na]+	Coumarins and derivatives	98.0	[8,8-Dimethyl-10-(3-Methylbut-2-Enoyloxy)-2-Oxo-9,10-Dihydropyrano[2,3-F]Chromen-9-Yl] 3-Methylbutanoate	C24H28O7	351.1211,245.0805,227.0697,175.0385,57.07	Level 2	51136480
C2	Authentic standards-MS-DIAL	10.70	274.2730	[M+H]+	Others	97.5	Lauryldiethanolamine	C16H35NO2	256.2633,230.2476,88.0757,70.0647	Level 2	352309
C3	Authentic standards-MS-DIAL	7.26	303.0502	[M+H]+	Flavonoids and derivatives	97.0	2-(3,4-Dihydroxyphenyl)-3,5,7-Trihydroxy-4H-Chromen-4-One	C15H10O7	257.0438,229.0507,201.0557,165.0186,153.0205,137.025	Level 2	9839360
C4	Authentic standards-MS-DIAL	8.52	287.0541	[M+H]+	Flavonoids and derivatives	96.7	Kaempferol	C15H10O6	213.0543,165.0184,153.0183,137.0226,121.0291	Level 1	5280863
C5	Authentic standards-MS-DIAL	20.14	449.1559	[M+Na]+	Coumarins and derivatives	96.4	[8,8-Dimethyl-9-(3-Methylbut-2-Enoyloxy)-2-Oxo-9,10-Dihydropyrano[2,3-F]Chromen-10-Yl] 3-Methylbut-2-Enoate	C24H26O7	349.1059,327.1231,251.0666,227.0697	Level 2	455809
C6	Authentic standards-MS-DIAL	3.75	203.0817	[M-H]-	Carboxylic acids and derivatives	96.3	Tryptophan	C11H12N2O2	159.0905,142.0673,116.0516,74.0259	Level 2	1148
C7	Authentic standards-MS-DIAL	2.66	245.0931	[M-H]-	Carboxylic acids and derivatives	96.2	N-Acetyltryptophan	C13H14N2O3	203.0839,116.0494,98.0251,74.0242,58.0296	Level 2	2002

C8	Authentic standards-MS-DIAL	22.71	453.1878	[M+Na]+	Coumarins and derivatives	95.3	[(9R,10R)-8,8-Dimethyl-9-(3-Methylbutanoyloxy)-2-Oxo-9,10-Dihydropyrano[2,3-F]Chromen-10-Yl] 3-Methylbutanoate	C24H30O7	351.1214,245.0807,175.0387,57.07	Level 2	10477794
C9	Authentic standards-MS-DIAL	15.97	329.1372	[M+H]+	Coumarins and derivatives	94.9	2-(7-Oxo-2,3-Dihydrofuro[3,2-G]Chromen-2-Yl)Propan-2-Yl (Z)-2-Methylbut-2-Enoate	C19H20O5	247.0951,229.0861,213.054,83.0485	Level 2	21670035
C10	Authentic standards-MS-DIAL	10.56	261.1111	[M+H]+	Coumarins and derivatives	94.8	8-(2-Hydroxy-3-Methylbut-3-Enyl)-7-Methoxychromen-2-One	C15H16O4	243.1027,189.0583,131.0502,103.054	Level 2	13343540
C11	MassBank	5.89	317.0657	[M+H]+	Flavonoids and derivatives	94.4	Isorhamnetin	C16H12O7	302.0422,285.0396,229.049,153.0179,93.032	Level 2	5281654
C12	Authentic standards-MS-DIAL	16.56	404.1706	[M+NH4]+	Coumarins and derivatives	94.3	(9-Acetyloxy-8,8-Dimethyl-2-Oxo-9,10-Dihydropyrano[2,3-F]Chromen-10-Yl) (Z)-2-Methylbut-2-Enoate	C21H22O7	287.0898,245.0809,227.0701,175.0389	Level 2	511787
C13	Authentic standards-MS-DIAL	15.31	301.1068	[M+H]+	Coumarins and derivatives	94.1	4-Methoxy-9-(3-Methylbut-2-Enoxy)Furo[3,2-G]Chromen-7-One	C17H16O5	233.0517,218.0278,162.0319	Level 2	98608
C14	Authentic standards-MS-DIAL	9.98	247.0606	[M+H]+	Coumarins and derivatives	93.7	Isopimpinellin	C13H10O5	232.0401,217.0176,189.0194,161.0231	Level 2	68079
C15	GNPS	16.74	229.0853	[M+H]+	Coumarins and derivatives	93.7	8-Prop-1-En-2-Yl-8,9-Dihydrofuro[2,3-H]Chromen-2-One	C14H12O3	,213.0566187.045,175.044,159.0455,131.0512,91.057	Level 2	5318874
C16	GNPS	16.74	329.1372	[M+H]+	Coumarins and derivatives	93.5	Ncgc00095690-03!(8,8-Dimethyl-2-Oxo-9,10-Dihydropyrano[2,3-H]Chromen-9-Yl) (Z)-2-Methylbut-2-Enoate	C19H20O5	229.0904,187.0437,175.0376,131.049	Level 2	511786

C17	Authentic standards-MS-DIAL	14.83	205.1585	[M-H]-	Benzene and substituted derivatives	93.1	4-Octylphenol	C14H22O	189.1273	Level 2	15730
C18	Authentic standards-MS-DIAL	8.41	271.0582	[M-H]-	Flavonoids and derivatives	92.5	Naringenin	C15H12O5	177.0184,151.0039,119.0498,65.0023	Level 1	932
C19	Authentic standards-MS-DIAL	16.05	271.0945	[M+H]+	Coumarins and derivatives	92.3	4-(3-Methylbut-2-Enoxy)Furo[3,2-G]Chromen-7-One	C16H14O4	203.0336,147.0447,131.049,91.0537	Level 2	68081
C20	GNPS	22.71	448.2318	[M+NH4]+	Coumarins and derivatives	92.0	[(9R,10R)-8,8-Dimethyl-9-(3-Methylbutanoyloxy)-2-Oxo-9,10-Dihydropyrano[2,3-F]Chromen-10-Yl] 3-Methylbutanoate	C24H30O7	329.1379,245.0804,227.0711,175.0385	Level 2	455807
C21	GNPS	5.26	303.0508	[M+H]+	Flavonoids and derivatives	91.5	5,7-Dihydroxy-2-(2,3,4-Trihydroxyphenyl)Chromen-4-One	C15H10O7	285.0396,257.0437,229.0491,153.0192, 137.0238	Level 2	18471844
C22	Fiehn/Vaniya natural product library	3.85	325.0912	[M+H]+	Coumarins and derivatives	91.2	Skimmin	C15H16O8	163.0417,119.0488,107.0489,91.0548	Level 2	320361
C23	Authentic standards-MS-DIAL	10.54	339.0828	[M+Na]+	Coumarins and derivatives	90.9	Byakangelicol	C17H16O6	254.0182,239.0018	Level 2	616062
C24	GNPS	11.54	253.1803	[M+H]+	Terpenes and derivatives	90.7	2-(8-Hydroxy-4A,8-Dimethyl-1,2,3,4,5,6,7,8A-Octahydronaphthalen-2-Yl)Prop-2-Enoic Acid	C15H24O3	189.0342,163,0569	Level 2	496073
C25	Authentic standards-MS-DIAL	20.14	327.1220	[M-H2O+H]+	Others	90.5	11-Methyl-2,7-Dimethylidene-6,12-Dioxo-5,14-	C19H20O6	227.0699,175.0375,83.0493,55.0544	Level 2	14807501

							Dioxatricyclo[9.2.1.0?,?]Tetradec-1(13)-En-9-Yl 2-Methylprop-2-Enoate				
C26	Authentic standards-MS-DIAL	10.47	285.0732	[M-H]-	Flavonoids and derivatives	90.3	Isosakuranetin	C16H14O5	243.0648,146.0095,136.0138,108.0195,65.0027	Level 2	5118250
C27	MassBank	6.80	336.1227	[M]+	Others	90.2	Berberine	C20H18NO4	306.077,292.095,278.0818	Level 2	2353
C28	Authentic standards-MS-DIAL	21.21	339.1583	[M+H]+	Others	90.0	9-{[(2E)-3,7-Dimethyl-2,6-Octadienyl]Oxy}-7H-Furo[3,2-G]Chromen-7-One	C21H22O4	203.0333,175.0399,137.1337,95.0849	Level 2	155658
C29	Authentic standards-MS-DIAL	9.46	361.0924	[M+H]+	Flavonoids and derivatives	90.0	5,7-Dihydroxy-2-(4-Hydroxy-3-Methoxyphenyl)-3,6-Dimethoxychromen-4-One	C18H16O8	345.0568,328.055,303.0479	Level 2	5464461
C30	Fiehn/Vaniya natural product library	12.68	261.1110	[M+H]+	Coumarins and derivatives	89.8	6-Methoxy-7-(3-Methylbut-2-Enoxy)Chromen-2-One	C15H16O4	193.0504,178.026,150.0309,133.0277	Level 2	1764914
C31	Fiehn/Vaniya natural product library	6.80	229.0856	[M+H]+	Coumarins and derivatives	89.6	Xanthyletin	C14H12O3	214.0608,213.0554,211.0645	Level 2	65188
C32	Authentic standards-MS-DIAL	11.60	259.0965	[M-H]-	Benzene and substituted derivatives	89.3	Peucenin	C15H16O4	204.0426,134.0361	Level 2	68477
C33	Fiehn/Vaniya natural product library	19.06	299.1625	[M+H]+	Coumarins and derivatives	88.9	Ostruthin	C19H22O3	243.1086,231.1017,175.0388	Level 2	5357655

C34	Authentic standards-MS-DIAL	14.07	389.1226	[M+H]+	Flavonoids and derivatives	88.1	Artemitin	C20H20O8	373.0922,356.0884,331.0810，313.0701	Level 1	5320351
C35	Authentic standards-MS-DIAL	11.75	219.1734	[M-2H2O+H]+	Terpenes and derivatives	87.5	5-Hydroxyculmorin	C15H26O3	201.1638,175.1503,145.101,105.0694	Level 2	122706895
C36	Authentic standards-MS-DIAL	15.42	303.2296	[M-H]-	Carboxylic acids and derivatives	86.9	Arachidonic Acid	C20H32O2	259.2393,231.2127,59.0121	Level 2	444899
C37	Fiehn/Vaniya natural product library	10.41	343.0790	[M-H]-	Flavonoids and derivatives	86.5	5,7-Dihydroxy-3,6-Dimethoxy-2-(4-Methoxyphenyl)Chromen-4-One	C18H16O7	328.057,313.0336,198.012,270.0147	Level 2	5281695
C38	Authentic standards-MS-DIAL	8.31	357.1321	[M-H]-	Lignans and derivatives	86.3	Pinoresinol	C20H22O6	342.1063,151.0398,136.0161	Level 2	234817
C39	Authentic standards-MS-DIAL	21.75	327.1223	[M-H2O+H]+	Coumarins and derivatives	86.2	Isolaserpitin	C19H20O6	227.0699,83.0493,55.0536	Level 2	511783
C40	Fiehn/Vaniya natural product library	5.90	331.1714	[M-H]-	Carbohydrates and carbohydrate conjugates	86.0	(3R,4S,5S,6R)-2-(6-Hydroxy-2,6-Dimethylocta-2,7-Dienoxy)-6-(Hydroxymethyl)Oxane-3,4,5-Triol	C16H28O7	113.0249,101.0241,85.0288,71.0135,59.013	Level 2	72970728
C41	Fiehn/Vaniya natural product library	8.68	329.0646	[M-H]-	Flavonoids and derivatives	85.8	(2Z)-4,6-Dihydroxy-2-[(4-Hydroxy-3,5-Dimethoxyphenyl)Methylidene]-1-Benzofuran-3-One	C17H14O7	314.0416,299.0176,271.0236	Level 2	490065

C42	Authentic standards-MS-DIAL	8.72	299.0555	[M-H]-	Flavonoids and derivatives	85.6	Tectorigenin	C16H12O6	284.0314,256.0358,227.0348	Level 2	5281811
C43	MassBank	12.42	315.0858	[M+H]+	Flavonoids and derivatives	85.5	Velutin	C17H14O6	300.0624,272.0672	Level 1	5464381
C44	Authentic standards-MS-DIAL	2.86	318.1544	[M+NH4]+	Benzene and substituted derivatives	85.4	(2S,3R,4S,5S,6R)-2-[4-(2-Hydroxyethyl)Phenoxy]-6-(Hydroxymethyl)Oxane-3,4,5-Triol	C14H20O7	145.0495,121.0646,85.0288	Level 2	14539961
C45	Authentic standards-MS-DIAL	8.85	357.1318	[M-H]-	Lignans and derivatives	85.4	Matairesinol	C20H22O6	342.1096,137.0592,211.0826,83.0131	Level 2	345761
C46	Fiehn/Vaniya natural product library	5.20	361.0905	[M+Na]+	Coumarins and derivatives	85.2	Gerberinside	C16H18O8	199.0373,185.0467	Level 2	14427391
C47	Authentic standards-MS-DIAL	6.41	247.1311	[M-H]-	Terpenes and derivatives	84.5	(3Ar,5Ar,9Br)-3A-Hydroxy-5A,9-Dimethyl-3-Methylidene-4,5,6,7,8,9B-Hexahydrobenzo[G][1]Benzofuran-2-One	C15H20O3	203.1423,163.1112,149.0966	Level 2	13995523
C48	Authentic standards-MS-DIAL	14.45	415.2119	[M+Na]+	Terpenes and derivatives	84.4	(1S,2R,4Ar,8Ar)-1-Acetoxy-7-Isopropylidene-1,4A-Dimethyl-6-Oxodecahydro-2-Naphthalenyl 2,3-Dimethyl-2-Oxiranecarboxylate	C22H32O6	119.085,91.0546	Level 2	14414238
C49	Authentic standards-MS-DIAL	7.02	231.1012	[M+H]+	Coumarins and derivatives	83.7	Osthenol	C14H14O3	175.0389,147.0436,91.0537	Level 2	5320318
C50	Authentic standards-MS-DIAL	22.11	339.1567	[M+H]+	Others	83.6	Bergamottin	C21H22O4	203.0333,147.0446	Level 2	119057

C51	Authentic standards-MS-DIAL	14.12	345.1301	[M+H]+	Coumarins and derivatives	82.9	Lasepitin	C19H20O6	227.0701,83.0485,55.0544	Level 2	511782
C52	Fiehn/Vaniya natural product library	5.37	365.0851	[M+K]+	Coumarins and derivatives	82.4	7-[(E)-3-Methyl-4-(4-Methyl-5-Oxo-2H-Furan-2-Yl)But-2-Enoxy]Chromen-2-One	C19H18O5	203.0338,147.0437	Level 2	337783
C53	GNPS	15.03	415.2113	[M-H2O+H]+	Terpenes and derivatives	82.3	(12-Acetyloxy-5,11,15,15-Tetramethyl-6-Oxo-2,7-Dioxapentacyclo[8.8.0.01,3.04,8.011,16]Octadec-4-En-14-Yl) Acetate	C24H32O7	119.085,91.0537	Level 2	138376535
C54	Authentic standards-MS-DIAL	19.42	286.2147	[M+H]+	Others	82.1	1-Methyl-2-Nonylquinolin-4-One	C19H27NO	172.1105,130.0647	Level 2	13967189
C55	GNPS	7.82	219.1735	[M-H2O+H]+	Terpenes and derivatives	82.1	6-Hydroxy-3,8-Dimethyl-5-Propan-2-Yl-4A,5,6,7,8,8A-Hexahydro-1H-Naphthalen-2-One	C15H24O2	219.1739,119.085	Level 2	74494816
C56	Fiehn HILIC	12.72	275.2004	[M+H]+	Steroids and steroid derivatives	81.7	Nandrolone	C18H26O2	257.1895,199.1463,173.1328,119.085	Level 2	220503
C57	Authentic standards-MS-DIAL	18.66	233.1319	[M-2H2O+H]+	Others	81.5	3,6-Diphenylcyclohexane-1,2-Diol	C18H20O2	191.0877,117.0691,91.0537	Level 2	14685115
C58	Authentic standards-MS-DIAL	4.38	391.1959	[M+CH3COO]-	Terpenes and derivatives	80.9	2-(Hydroxymethyl)-6-(6-Hydroxy-6-Methyl-3-Propan-2-Ylcyclohex-3-En-1-Yl)Oxyoxane-3,4,5-Triol	C16H28O7	101.0251,71.0135,59.0137	Level 2	56776223
C59	Authentic standards-MS-DIAL	4.28	225.1496	[M+H]+	Others	79.8	2-(6-Methyl-7-Oxooctyl)-2H-Furan-5-One	C13H20O3	189.1299,151.1109,119.0841,77.0382	Level 2	11075054

C60	Authentic standards-MS-DIAL	5.52	213.0765	[M+H]+	Benzene and substituted derivatives	79.2	Methyl 4-Hydroxy-3,5-Dimethoxybenzoate	C10H12O5	181.0477,154.0611,139.0377	Level 2	70164
C61	BMDMS-NP	10.63	287.0903	[M+H]+	Benzene and substituted derivatives	79.1	Licochalcone B	C16H14O5	203.0421,147.0496,67.0583	Level 2	480788
C62	GNPS	5.50	343.1178	[M+H]+	Benzene and substituted derivatives	78.9	1,3-Cyclobutanedicarboxylic Acid, 2,4-Bis(4-Hydroxyphenyl)-, Monomethyl Ester	C19H18O6	325.1081,2565.0862,91.0548	Level 2	56776249
C63	Authentic standards-MS-DIAL	20.87	273.2560	[M-H2O+H]+	Terpenes and derivatives	78.4	(Z)-5-(5,5,8A-Trimethyl-2-Methylidene-3,4,4A,6,7,8-Hexahydro-1H-Naphthalen-1-Yl)-3-Methylpent-2-En-1-Ol	C20H34O	203.1786,81.0704	Level 2	75528899
C64	GNPS	3.89	265.1433	[M-H2O+H]+	Terpenes and derivatives	77.4	3-(8-Hydroxy-6,8-Dimethyl-3-Methylidene-2-Oxo-3A,4,5,6,7,8A-Hexahydrocyclohepta[B]Furan-7-Yl)Propanoic Acid	C15H22O5	201.1268,175.0769,119.0853,91.054	Level 2	75110934
C65	MassBank	12.00	301.0725	[M+H]+	Flavonoids and derivatives	76.6	Kaempferide	C16H12O6	286.0461,153.0177	Level 2	5281666
C66	Fiehn/Vaniya natural product library	19.53	305.2463	[M+H]+	Terpenes and derivatives	76.0	(4S,4Ar,8As)-4-[(3R)-3-Hydroxy-3-Methylpent-4-Enyl]-3,4A,8,8-Tetramethyl-5,6,7,8A-Tetrahydro-4H-Naphthalen-1-One	C20H32O2	121.0997,79.0544	Level 2	24721562
C67	Authentic standards-MS-DIAL	5.41	225.1480	[M+H]+	Carboxylic acids and derivatives	75.6	Methyl 2-[(1R,2R)-3-Oxo-2-[(Z)-Pent-2-Enyl]Cyclopentyl]Acetate	C13H20O3	207.1362,151.1122,93.0699	Level 2	5281929
C68	GNPS	8.69	263.1626	[M-H2O+H]+	Others	75.6	4-Hydroxy-1-Methoxy-6,6,9A-Trimethyl-4,5,5A,6,7,8,9,9A-Octahydronaphtho[1,2-C]Furan-3(1H)-One	C16H24O4	245.1514,135.0792,91.0546	Level 2	45783042

C69	GNPS	26.39	307.2614	[M-H2O+H]+	Terpenes and derivatives	75.0	5-(2-Hydroxy-2,5,5,8A-Tetramethyl-3,4,4A,6,7,8-Hexahydro-1H-Naphthalen-1-Yl)-3-Methylpentanoic Acid	C20H36O3	137.1314,123.1162,109.1005	Level 2	3430304
C70	Authentic standards-MS-DIAL	23.97	259.2044	[M+H]+	Terpenes and derivatives	74.2	6-Acetyl-1,1,2,4,4,7-Hexamethyltetralin	C18H26O	175.1478,119.085	Level 2	89440
C71	RIKEN PlaSMA bio-MS/MS from plant tissues	2.79	305.0664	[M-H]-	Carboxylic acids and derivatives	73.9	2-[3-Oxo-2-(5-Sulfooxypent-2-Enyl)Cyclopentyl]Acetic Acid	C12H18O7S	225.1128,96.959,59.0131	Level 2	75075824
C72	Fiehn/Vaniya natural product library	5.76	265.1411	[M-H]-	Terpenes and derivatives	73.9	(4Ar,5S)-9,9A-Dihydroxy-3,4A,5-Trimethyl-5,6,7,8,8A,9-Hexahydro-4H-Benzo[F][1]Benzofuran-2-One	C15H22O4	247.1324,221.1532,203.1426	Level 2	75110996
C73	RIKEN PlaSMA bio-MS/MS from plant tissues	22.13	387.2529	[M+H]+	Steroids and steroid derivatives	73.8	Bufalin	C24H34O4	369.2379,351.2333	Level 2	2464
C74	Authentic standards-MS-DIAL	13.05	203.0694	[M+H]+	Coumarins and derivatives	73.0	7-Hydroxy-2,3-Dihydrocyclopenta[C]Chromen-4(1H)-One	C12H10O3	175.0387,147.0446,91.0546	Level 2	5322196
C75	Fiehn/Vaniya natural product library	7.44	219.1724	[M+H]+	Terpenes and derivatives	72.9	Muurolladie-3-One	C15H22O	147.1163,107.0854	Level 2	3989419
C76	Authentic standards-MS-DIAL	22.63	293.2455	[M+H]+	Steroids and steroid derivatives	72.4	Androstane-3,17-Diol	C19H32O2	275.2005,109.1005,95.0839,81.0695	Level 2	11301

C77	GNPS	13.71	249.1845	[M-H2O+H]+	Terpenes and derivatives	71.2	3-Hydroxy-1-[(1R,2R,4Ar,6S,8As)-1,2,4A,5,6,7,8,8A-Octahydro-2-Hydroxy-1,2,6-Trimethyl-1-Naphthalenyl]-1-Propanone	C16H26O3	213.1638,119.0849,93.0697,79.0544	Level 2	73837707
C78	GNPS	8.40	203.1799	[M-H2O+H]+	Terpenes and derivatives	82.6	1-Methyl-4-Methylidene-7-Propan-2-Yl-2,3,3A,5,6,8A-Hexahydroazulen-1-Ol	C15H24O	175.1467,147.1175,105.0696	Level 2	73102746
C79	GNPS	5.41	303.0133	[M+H]+	Benzene and substituted derivatives	88.5	6,7,13,14-Tetrahydroxy-2,9-Dioxatetracyclo[6.6.2.04,16.011,15]Hexadeca-1(15),4,6,8(16),11,13-Hexaene-3,10-Dione	C14H6O8	257.0452,229.049,201.0542,137.0238	Level 2	5281855
C80	GNPS	12.07	325.1830	[M-H]-	Others	94.0	(5-Ethyl-1-Azabicyclo[2.2.2]Octan-2-Yl)-(6-Methoxyquinolin-4-Yl)Methanol	C20H26N2O2	197.0202,184.0122	Level 2	3649
C81	GNPS	12.74	339.1982	[M-H]-	Steroids and steroid derivatives	91.7	(9S,14S)-10,13-Dimethylspiro[2,8,9,11,12,14,15,16-Octahydro-1H-Cyclopenta[A]Phenanthrene-17,5'-Oxolane]-2',3-Dione	C22H28O3	183.0110,170.0039	Level 2	24728626
C82	GNPS	5.36	373.1846	[M-H]-	Carboxylic acids and derivatives	72.3	2-[2-[(Z)-Pent-2-Enyl]-3-[3,4,5-Trihydroxy-6-(Hydroxymethyl)Oxan-2-Yl]Oxycyclopentyl]Acetic Acid	C18H30O8	101.0241,59.0137	Level 2	45360381
C83	GNPS	8.09	417.1489	[M-H]-	Lignans and derivatives	71.6	4-[(3Ar,6S,6Ar)-6-(4-Hydroxy-3,5-Dimethoxyphenyl)-1,3,3A,4,6,6A-Hexahydrofuro[3,4-C]Furan-3-Yl]-2,6-Dimethoxyphenol	C22H26O8	387.1086,181.0507,59.0137	Level 2	332426
C84	GNPS-MS1	6.70	201.0552	[M+H]+	Coumarins and derivatives		3-Methylfuro[3,2-G]Chromen-7-One	C12H8O3	173.0608,115.0547,91.0538	Level 3	608273
C85	GNPS-MS1	6.13	328.1174	[M+H]+	Others		3-(3-Acetylanilino)-6,7-Dimethoxy-3H-2-Benzofuran-1-One	C18H17NO5	269.0803,203.0335,84.0796	Level 3	3394829

C86	GNPS-MS1	8.72	329.0999	[M+H]+	Others	3-[2-(4-Hydroxyphenyl)-2-Oxoethyl]-6,7-Dimethoxy-3H-2-Benzofuran-1-One	C18H16O6	269.0804,201.0179,173.0232	Level 3	2863812
C87	GNPS-MS1	7.94	331.0800	[M+H]+	Others	2-[(4,6-Dihydroxy-7-Methyl-3-Oxo-1H-2-Benzofuran-5-Yl)Oxy]-6-Hydroxy-4-Methylbenzaldehyde	C17H14O7	204.0347,81.0688	Level 3	76764468
C88	GNPS-MS1	3.57	335.0755	[M-H]-	Benzene and substituted derivatives	1,3,4-Trihydroxy-5-(1-Oxoisochromen-4-Yl)Oxycyclohexane-1-Carboxylic Acid	C16H16O8	179.0352,135.0456,93.0347	Level 3	163074473
C89	GNPS-MS1	5.51	392.1330	[M+H]+	Benzene and substituted derivatives	2-(4,8-Dimethoxyfuro[2,3-B]Quinolin-7-Yl)Oxy-6-Methyloxane-3,4,5-Triol	C19H21NO8	269.0823,203.0338,60.0442	Level 3	3518320
C90	GNPS-MS1	4.28	398.1096	[M+H]+	Flavonoids and derivatives	9-(1,1-Dioxothiolan-3-Yl)-3-Phenyl-8,10-Dihydropyrano[2,3-F][1,3]Benzoxazin-4-One	C21H19NO5S	219.0292,97.0273	Level 3	17601724
C91	GNPS-MS1	6.33	426.1523	[M+H]+	Coumarins and derivatives	2-(7,8-Dimethoxy-4-Methyl-2-Oxochromen-3-Yl)-N-[2-(4-Methoxyphenyl)-2-Oxoethyl]Acetamide	C23H23NO7	309.0745,225.0161	Level 3	71755053
C92	GNPS-MS1	6.75	440.1667	[M+H]+	Flavonoids and derivatives	2-[[2-[4-(4-Methoxyphenyl)-8-Methyl-2-Oxochromen-7-Yl]Oxyacetyl]Amino]Pentanoic Acid	C24H25NO7	309.0728,203.0337	Level 3	4357287
C93	GNPS-MS1	7.15	452.1707	[M+H]+	Others	4-[1-[4-Hydroxy-1-[2-(4-Hydroxyphenyl)Ethyl]-6-Methyl-2-Oxopyridin-3-Yl]-3-Methoxy-3-Oxopropyl]Benzoic Acid	C25H25NO7	452.1717,269.0807,120.0808	Level 3	102564695
C94	GNPS-MS1	0.80	467.0786	[M+H]+	Flavonoids and derivatives	Methyl 4-[[6-(4-Methoxyphenyl)Sulfonyloxy-3-Oxo-1-Benzofuran-2-Ylidene]Methyl]Benzoate	C24H18O8S	265.1590,135.079	Level 3	72185813

Table S4 The Molecular Docking Grid Boxes Parameters

Target Protein	Grid Box Center (X, Y, Z)	Grid Box Dimensions (X×Y×Z, Å^3)	Grid Spacing (Å)
TNF	0.178, 32.124, 53.949	50×46×50	1.00
IL-6	2.669, -19.932, 11.213	40×44×40	1.00
IL-1β	22.392, 1.593, 74.743	40×40×40	1.00
GAPDH	113.841, 86.016, 111.47	48×52×48	1.00
AKT1	23.107, 0.636, 8.705	40×40×40	1.00
